# Bioinspired architected catalyst for efficient water purification: Bridging reactivity, reusability, and catalytic component utilization

**DOI:** 10.1126/sciadv.aeb0624

**Published:** 2026-08-05

**Authors:** Xirui Zhou, Songyan Zhang, Hanzheng Xing, Ni Yan, Xia Huang, Xuan Zhang, Xiaoyuan Zhang, Xiaoyan Li

**Affiliations:** ^1^ Mechano-X Institute, Tsinghua University, Beijing 100084, China.; ^2^ State Key Laboratory of Regional Environment and Sustainability, School of Environment, Tsinghua University, Beijing 100084, China.; ^3^ Applied Mechanics Laboratory, Department of Engineering Mechanics, Tsinghua University, Beijing 100084, China.

## Abstract

Achieving highly efficient water purification remains a formidable challenge due to intricate coupling among catalytic efficiency, mechanical robustness, and mass transport. Here, we create a bioinspired architected catalyst featuring microscale shell-based frameworks with atomically dispersed Mn-N_4_ sites, fabricated by digital light processing and ultrasonic decoration. Their synergy—where the starfish- and bone-inspired architecture minimizes stress concentration for reusability and enhances pollutant-site contact for mass transport, while the Mn-N_4_ sites facilitate •OH-mediated oxidation—collectively amplifies overall performance beyond the contributions of either component alone. Compared with conventional pellet catalysts, the architected catalyst exhibits a 22.1-fold increase in strength and a 4.56-fold increase in normalized reaction kinetics, resulting in more than 95% degradation with 0.08% active component consumption. It occupies the previously unattained region in the catalytic efficiency (*K* value) versus metal utilization (active component content) diagram of reported powder and supported catalysts. This work demonstrates a generalizable strategy for designing catalysts that unite reactivity, reusability, and catalytic component utilization.

## Teaser

A bioinspired architected catalyst cleans wastewater powerfully, stands up to reuse, and uses less active component.

## INTRODUCTION

Severe water scarcity and pollution caused by continuous industrialization and other human activities have driven the increased demand for water reclamation and are among the most pervasive problems affecting people’s sustainable development (*1*, *2*). Thus, efficient and economically viable water decontamination technologies are in high demand (*3*). Advanced oxidation processes, which rely mostly on the reactive oxygen species (ROS) generated in reactions with catalysts, are pivotal options for degrading organic pollutants (*4*). Notably, the catalytic process for water purification is complex as it involves behaviors at the liquid-solid-gas three-phase interface. This complexity necessitates multiscale structural control of the catalyst to ensure that each reaction step proceeds smoothly (*5*–*8*). This includes enough catalytic capability, sufficient accessibility to active sites, and robust mechanical strength for long-term reuse. The microscale framework can provide mechanical robustness. Meanwhile, the mesoporous structure can promote efficient mass transport of reactants via interconnected channels and fluid turbulence regulation. In contrast, the degradation of contaminants predominantly occurs at nanoscale or atomic-scale reactive sites such as defect regions or single-atom centers (*9*–*12*). The two conventional catalysts, powder and supported catalysts, have demonstrated specific advantages in leveraging these structural characteristics. Powder catalysts, with their high surface area and abundant exposed active sites, typically exhibit high intrinsic catalytic activity. Supported catalysts, on the other hand, benefit from the integration of catalytic species with macroscale carriers, which facilitates separation and reuse and reduces the overall consumption of active materials. Nevertheless, developing catalysts that simultaneously integrate multiple functionalities—such as high reactivity, long-term reusability, and efficient catalytic component utilization—remains a major challenge. This is because most existing synthetic strategies are tailored to optimize only a single structural dimension (*13*, *14*). The inherent difficulty of coupling properties across multiple length scales cannot be fully addressed by conventional material modulations, such as etching or hydrothermal synthesis (*15*). Consequently, developing an effective strategy for multiscale-integrated catalysts is crucial toward practical and efficient water purification.

Recently, architected materials with engineered geometries at the micro- and nanoscale have emerged as a promising materials system. Fabricated by three-dimensional (3D) printing techniques, these materials achieve unique mechanical and practical properties beyond those of conventional materials (*16*, *17*). With their unique structural configurations and high design freedom, architected materials enable the efficient coupling of properties such as mechanical strength and energy conversion (*18*). In addition, the precise control of architected materials leads to more predictable and controllable flow patterns in the pores, enhancing the transport of reactants to active sites. Combined with their intrinsic mechanical stability, these features are driving the increasing application of architected materials in chemical processes and environmental remediation (*19*–*21*). Furthermore, the wide selection of base materials used for architected structures allows for postsynthetic chemical or biological modifications, enabling further functional optimization at the atomic scale. For example, metal-organic framework (MOF)-loaded hierarchical porous ceramics have been reported (*22*). Owing to their hierarchical transport channels, these materials exhibit high performance in degrading organic dyes via Fenton reactions. The controllability and stability of these architected catalysts enable them to be adaptable to complex environments during pollutant degradation (*23*), implying a possibility for addressing the overall design of multifunctional catalysts. However, current studies on multifunctional architected catalysts largely remain empirical, with limited precision in tuning structures across multiple scales. A fundamental understanding of the synergies across scales and intrinsic coupling among mechanical robustness, mass transport, and catalytic properties is still lacking. This limitation directly hinders the rational design of fully multiscale architected catalysts for water purification.

Here, we present a bioinspired architected catalyst with single-atom manganese (Mn) for efficient water purification. The single-atom sites are chosen to load onto the architected alumina framework (AAF) for their superior catalytic activity and improved atom utilization over those metal-based nanomaterials to simultaneously promote catalytic efficacy (*24*–*28*). The bioinspired multiscale architected catalysts are developed at (i) macroscale/mesoscale: Shell-based architectures with both periodic and stochastic mesoporous channels minimize mechanical stress concentration to enhance mechanical properties for reusability. These architectures simultaneously increase internal surface area and interfacial shear velocity for reactant contact, thereby improving the utilization efficiency of catalytic active sites; (ii) atomic-scale: Mn-isolated single-atom (Mn-ISA) sites exhibit optimized electronic configurations and geometric arrangements, facilitating the formation of crucial intermediate species (HO_3_•), thereby significantly boosting hydroxyl radical generation and enhancing catalytic reactivity. The intrinsic synergy between the microenvironment-regulating architected framework and the highly efficient Mn-N_4_ sites enhances the overall catalytic performance. Compared with conventional pellet or powder catalysts, our Mn-isolated single-atom @AAF (Mn-ISAs@AAF) catalysts uniquely integrate the advantages of high reactivity, reusability, and catalytic component utilization, achieving exceptional pollutant removal efficiency and ultralow active component consumption in catalytic ozonation processes for both synthetic and real wastewater. Through comprehensive experimental investigations and multiscale modeling—including computational fluid dynamics (CFD), density functional theory (DFT), and molecular dynamics (MD) simulations—we elucidate the intrinsic mechanisms underlying the synergistic interactions among mechanical strength, transport pathways, and catalytic activity across multiple scales. This work provides a thorough and viable strategy for multiscale architected catalysts, overcoming the long-standing limitation that powder and supported catalysts cannot combine reactivity, reusability, and catalytic component utilization, and laying the groundwork for high efficiency in wastewater decontamination and other catalytic processes.

## RESULTS

### Bioinspired design and hierarchical construction of Mn-ISAs@AAFs

**Fig. 1.**
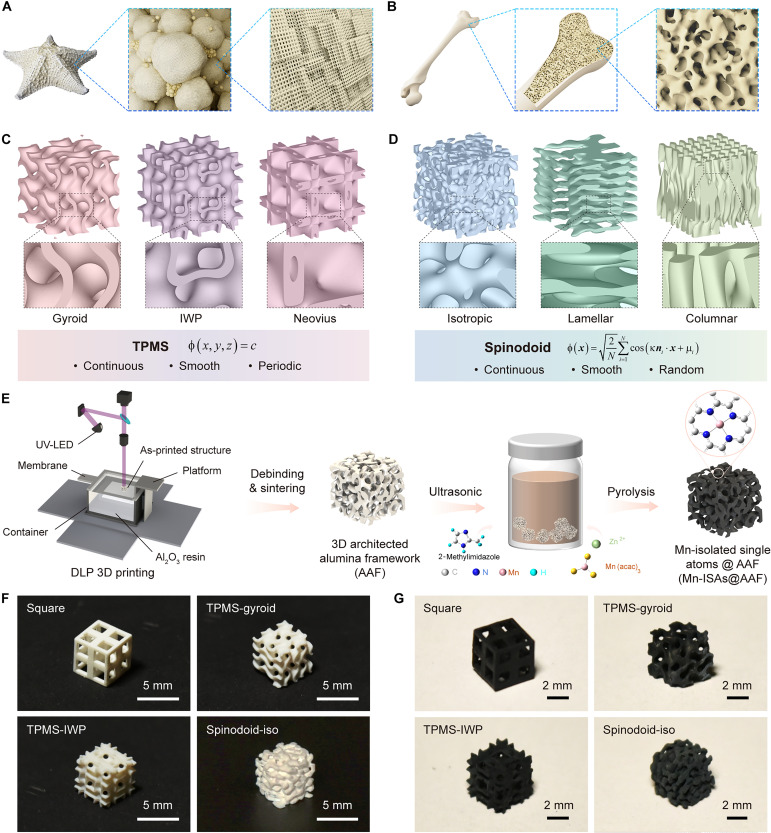
Design and fabrication of bioinspired architected catalysts with single-atom Mn. (**A** and **B**) Schematic illustration of starfish ossicles and bone trabeculae. (**C**) Schematic illustration of TPMS and its representative architectures. (**D**) Schematic illustration of spinodoid and its representative architectures. (**E**) Synthesis procedure for the bioinspired architected catalysts with single-atom Mn. LED, light-emitting diode. (**F**) Digital photographs of the as-prepared microarchitected alumina substrate. Scale bars, 5 mm. (**G**) Digital photographs of the Mn-isolated single-atom (Mn-ISA) catalyst after a series of postprocessing steps. Scale bars, 2 mm.

A catalyst framework design strategy that incorporates 3D continuous, smooth, and shell-based architectures inspired by natural sophisticated structures was proposed for highly efficient water purification. The periodic ossicles of starfish (Fig. 1A) and the random trabecular network of human bone (Fig. 1B) exemplify naturally optimized 3D architectures that combine continuous, smoothly curved surfaces with high structural interconnectivity. The dual-scale microlattices of starfish ossicles exhibit lightweight yet high-strength and damage-tolerant features (*29*), while trabecular bone presents a stochastic but highly interconnected rod-plate network with large surface area and efficient fluid/nutrient transport (*30*). Drawing inspiration from these biological templates, two biomimetic catalyst architectures emerge: triply periodic minimal surfaces (TPMSs) and stochastic structures based on biological spinodal decomposition. TPMS-based architected materials exhibit excellent mechanical and functional properties because of their smooth and continuous curved topologies (*31*–*33*). These lattice structures can be described using a level-set approximation equation, i.e., ϕ(*x*, *y*, *z*) = ±*c*. The constant *c* governs both the relative density and shell thickness. Figure 1C and table S1 summarize the geometries (with a relative density of 30%) and corresponding level-set equations for several common TPMS-based lattices, including gyroid, I-Wrapped Package (IWP), and Neovius topologies. Spinodal decomposition–architected material (referred to as a spinodoid) is an emerging topology characterized by smooth, continuous, and random geometry (Fig. 1D). Compared with periodic TPMS lattices, spinodoids have nearly zero mean curvature while exhibiting isotropic or tunable anisotropic elasticity, a uniform distribution of stress and strain during deformation, reduced sensitivity to imperfections, and increased energy absorption (*34*–*36*). The spinodoid topology can be numerically generated by a Gaussian random field (*37*), where the modulation of the statistical parameters of the wave vectors enables precise control of the relative density, pore morphology, and elastic anisotropy (fig. S1 and text S1). TPMS structures provide highly ordered, bicontinuous pathways with smooth curvature, uniform shear fields, excellent mechanical robustness, and well-distributed surface area, enabling stable and efficient mass transport. Spinodoid architectures introduce nonperiodic, tortuous, and diffusively connected channels that suppress flow channeling, extend residence time, and generate locally intensified shear velocity to enhance interfacial mass transfer. Table S2 benchmarks TPMS and spinodoid against conventional architectures, demonstrating their unique ability to reconcile connectivity, curvature, surface area, and mechanical robustness within a single framework. Consequently, both architectures offer distinct yet complementary advantages that directly address the catalytic requirements of reactivity, reusability, and catalytic component utilization.

To further facilitate highly efficient water purification, we developed a multistep fabrication process for bioinspired architected catalysts with single-atom Mn (Fig. 1E). Alumina resin was used to construct 3D architected frameworks via high-precision digital light processing (DLP) printing. A series of sliced images guide layer-by-layer photopolymerization of the precursor solution through patterned ultraviolet (UV) irradiation, which enabled the fabrication of precise 3D structures (text S2). Considering both the design strategy and structural formability, TPMS-gyroid, TPMS-IWP, isotropic spinodoid (denoted as Spinodoid-iso), and common square lattice framework controls were printed, each with a relative density of 30% (Fig. 1F). The square lattice, lacking any curvature or shell-based continuity, serves as a comparative structure to decouple the role of surface area enhancement from that of topological complexity. This comparison enables the evaluation of whether multiscale performance benefits arise from mere inner geometric surface increase or from intrinsically optimized architectures. Conventional solid pellets, obtained directly from commercially available products, were also included as a reference structure for subsequent synthesis and performance comparisons. The as-printed AAFs were subsequently subjected to debinding and sintering to obtain a dense ceramic structure, thereby improving the mechanical properties and catalyst loading efficiency. After postprocessing, the surface of the AAFs was modified with atomically dispersed Mn reactive sites via an ultrasonic sonochemical process. Ultrasonic process enables the uniform distribution of precursors throughout the porous 3D framework, thereby facilitating Mn precursor penetration into the nanocages and promoting the in situ growth of Mn@zeolitic imidazolate framework-8 (Mn@ZIF-8) on the AAF. This precursor layer is then transformed into a carbon coating hosting Mn single-atom sites during the subsequent pyrolysis to prepare multifunctional Mn-ISAs@AAF catalysts. The resulting strong interfacial bonding, arising from interactions between the alumina surface groups and the carbon layer, integrates the robust AAF with the carbon layer into a monolithic entity, crucial for long-term structural integrity. Microarchitected structures contribute to robust mechanical properties and sufficient internal surface area for reactant contact, whereas atomically dispersed Mn-ISA sites provide adequate active centers for high catalytic efficiency, collectively resulting in the formation of multifunctional architected catalysts (Fig. 1G). This multiscale synergy design establishes an intrinsic coupling among mechanical robustness, mass transport, and catalytic properties, thereby enabling the simultaneous enhancement of reactivity, reusability, and catalytic component utilization for water purification.

### Atomic-scale characterization of Mn-ISAs@AAF catalysts

**Fig. 2.**
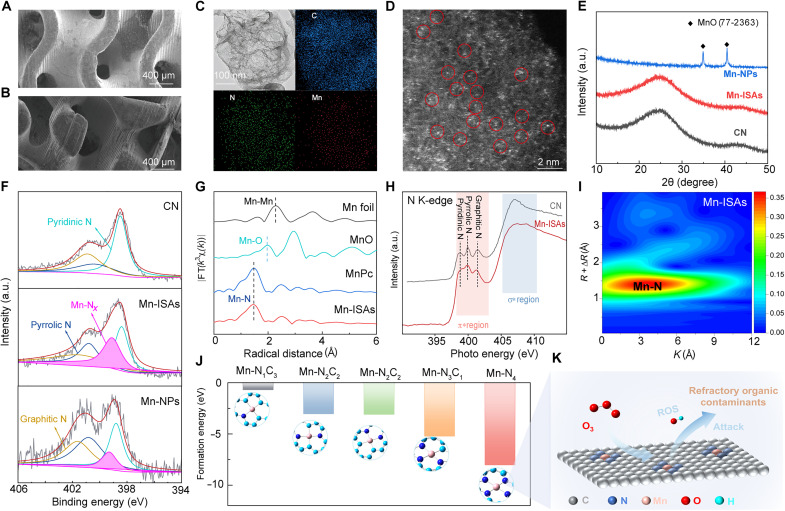
Morphology and structural characterization of catalyst reactive sites with single-atom Mn. (**A** and **B**) Scanning electron microscopy (SEM) images of the Mn-ISAs@AAF catalysts. (**C**) Element mapping images. (**D**) High-angle annular dark-field scanning transmission electron microscopy (HAADF-STEM) image. (**E**) X-ray diffraction (XRD) patterns of different catalysts. (**F**) N 1s XPS spectra of different catalysts. (**G**) Fourier transform extended x-ray absorption fine structure (FT-EXAFS) spectra of Mn-ISAs and reference samples (Mn foil, MnO, and MnPc). (**H**) N K-edge XANES spectra. (**I**) Wavelet transform (WT) plots. (**J**) Formation energy of different configurations. (**K**) Reaction mechanism on Mn-N_4_ sites.

To confirm the structural integrity and chemical configuration of the prepared Mn-ISAs@AAF catalysts, comprehensive microscopic and spectroscopic characterizations were performed. Figure 2 (A and B) shows two scanning electron microscopy (SEM) images of Mn-ISAs@AAF. These findings indicated that the designed TPMS-gyroid and Spinodoid-iso structures were completely preserved after the synthesis of Mn-ISAs@AAF. Unlike the independent AAF, the porous carbon layer with nano-reactive Mn sites covered the Mn-ISAs@AAF (fig. S2). To further demonstrate the surface-loaded active component, transmission electron microscopy (TEM) dark-field images and corresponding energy-dispersive spectroscopy (EDS) maps were obtained to investigate the powders scraped from the surface. The dark-field TEM image and corresponding EDS mappings (Fig. 2C) of the powders collected from catalyst surfaces revealed a homogeneous distribution of Mn, C, and N, suggesting uniform incorporation of the active components. High-angle annular dark-field scanning TEM (HAADF-STEM) images demonstrated isolated single-atom Mn sites without observable particle agglomeration (marked by red circles, Fig. 2D and figs. S3 and S4). X-ray diffraction (XRD) analysis provided further evidence for the atomic dispersion state of Mn. The XRD pattern of Mn-ISAs showed only a broad peak at ∼26° (2θ) corresponding to N-doped carbon (CN), without additional peaks attributable to crystalline Mn nanoparticles, indicating a high dispersion of atomic Mn. In contrast, samples loaded with higher Mn content (Mn-NPs@AAF) exhibited characteristic diffraction peaks of MnO (Joint Committee on Powder Diffraction Standards, no. 77-2363; Fig. 2E), signifying nanoparticle aggregation (*38*). The increased Mn content promoted enhanced graphitization of the carbon skeleton, as evidenced by Raman spectroscopy (fig. S5). This is attributed to the metal-catalyzed graphitization effect of Mn nanoparticles during pyrolysis. Collectively, these results unequivocally confirmed the atomic dispersion of Mn species on the Mn-ISAs@AAF surfaces.

To elucidate the local electronic structure and coordination environment of the Mn sites on bioinspired architectures, x-ray absorption near-edge structure (XANES) and Fourier transform extended x-ray absorption fine structure (FT-EXAFS) analyses were performed. The XANES spectrum (fig. S6) revealed that the absorption edge of Mn-ISAs fell between those of Mn foil and MnO, indicating a positively charged oxidation state of Mn (*39*). Furthermore, FT-EXAFS spectra of Mn-ISAs exhibited a prominent single peak at ∼1.4 Å, assigned specifically to the Mn-N scattering path, without Mn-Mn or Mn-O signals (Fig. 2G), aligning well with Mn 2p XPS spectra (fig. S7). The high-resolution N 1s XPS spectra of these catalysts (Fig. 2F) were deconvoluted into four binding types: pyridinic N, pyrrolic N, graphitic N, and Mn-N (*40*). Among them, the content of pyridinic N in the Mn-ISAs was greater than that in the other methods, in which a Mn-N peak also appeared. Notably, a slight shift in binding energy of pyridinic N was observed in Mn-ISAs, compared with CN and Mn-NPs (table S3). The observed shift is associated with stronger electronic perturbation of neighboring nitrogen species and subtle electron density redistribution, which are induced by atomically dispersed Mn. This further confirmed the existence of a Mn-N coordination structure, with a high content of pyridine nitrogen contributing to the reaction. The N K-edge XANES spectra (Fig. 2H) of the catalysts were dominated by three well-resolved resonance peaks, corresponding to pyridinic π*, pyrrolic π*, and graphitic π*. Compared with CN, the resonance of pyridinic N for Mn-ISAs showed a notable reduction. Meanwhile, in the σ* region, Mn doping resulted in a broader resonance profile compared with CN, which indicated the diverse lengths of C─N bonds. All these variations in the N K-edge XANES spectra demonstrated the alterations in the chemical environment of N atoms for Mn-ISAs due to the Mn doping, giving rise to the changed electronic configuration. Quantitative EXAFS fitting analysis further revealed that each isolated Mn atom was coordinated with four nitrogen atoms (Mn-N_4_ structure), as indicated by the fitting curves and structural parameters summarized in fig. S8 and table S4. To reinforce the above findings, wavelet transform (WT) EXAFS contour plots were constructed in a concise and lucidly visualized manner with high resolution in both *k* and *R* space. As shown in Fig. 2I, the WT plots of the Mn-ISAs only exhibited one intensity maximum, which is consistent with the Mn-N coordination. The structural stability of Mn-N_4_ was further verified by calculating the formation energy. As shown in Fig. 2J, Mn-N_4_ exhibited the lowest formation energy, indicating that it was thermodynamically the most stable configuration, consistent with the characterization results. Collectively, these detailed characterizations provide strong and consistent evidence that the optimized coordination environment for the single Mn atoms in Mn-ISAs@AAF catalysts is Mn-N_4_ configuration, which further serves as the reactive center for catalytic reactions (Fig. 2K). These atomically dispersed active sites are expected to deliver enhanced catalytic reactivity in water purification processes.

### Mechanical and mass transfer performance of microarchitected framework

**Fig. 3.**
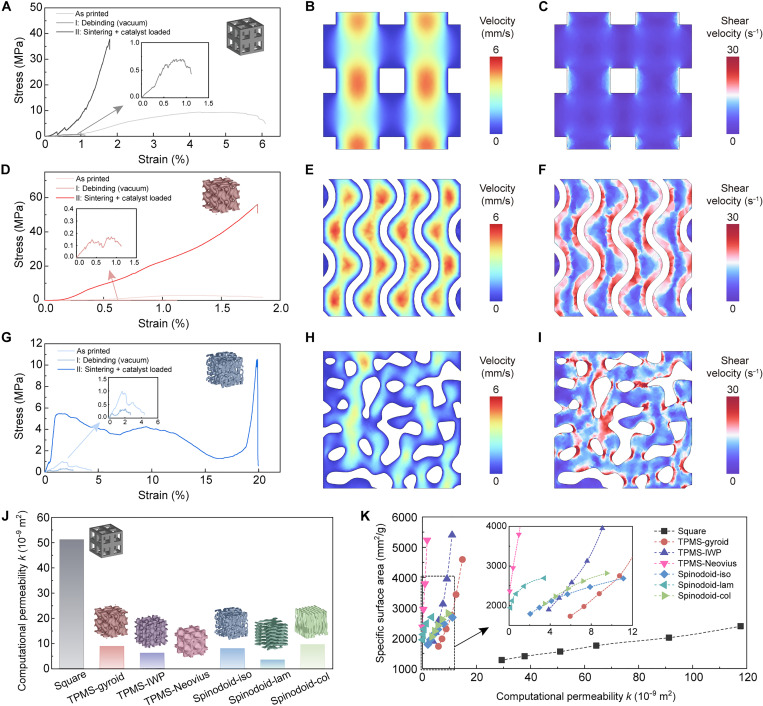
Mechanical properties and mass transfer of microarchitected catalyst frameworks. (**A**) Stress-strain curves of architected square lattices under compression. (**B** and **C**) Simulated distributions of the fluid velocity and shear velocity inside the square catalyst under laminar flow conditions, respectively. (**D**) Stress-strain curves of architected gyroid frameworks under compression. (**E** and **F**) Simulated distributions of the fluid velocity and shear velocity inside the gyroid catalyst under laminar flow conditions, respectively. (**G**) Stress-strain curves of architected Spinodoid-iso frameworks under compression. (**H** and **I**) Simulated distributions of the fluid velocity and shear velocity inside the Spinodoid-iso catalyst under laminar flow conditions, respectively. (**J**) Comparison of the permeability of different frameworks. (**K**) Specific surface area versus computational permeability of different frameworks.

To evaluate the mechanical stability of the AAFs, a series of mechanical tests were conducted to investigate the mechanical properties of the AAFs. Figure 3 (A, D, and G) shows the typical compressive stress-strain curves of the square lattice, TPMS, and spinodoid catalysts at different postprocessing stages, respectively. TPMS-gyroid frameworks exhibited significantly higher compressive strengths than the comparative square lattice under the same relative density (30%), demonstrating that the smooth and continuous shell-based architecture minimizes stress concentration more effectively than planar structures, leading to improved mechanical performance. Specifically, the sintered TPMS-gyroid framework reached a compressive strength of 54.7 MPa, and the Spinodoid-iso structure achieved 9.97 MPa. Compared with those of the widely used conventional pellet catalyst (2.48 MPa), the strengths of the TPMS-gyroid and Spinodoid-iso catalysts increased by 22.1 times and 4.0 times, respectively, underscoring the structural superiority of the bioinspired 3D architected designs. The exceptional strength is indispensable for structural integrity and long-term reusability. Hence, the high strength of TPMS and spinodoid structures forms the foundation for their potential long-term durability.

In addition to structural topology, postprocessing treatments such as debinding and sintering further improved mechanical robustness. For all architectures, the as-printed green bodies showed limited strength due to the fragile polymer network. Following vacuum debinding and sintering, ceramic consolidation occurred, leading to substantial increases in strength (fig. S9). After the debinding and sintering treatment, the compressive strength of the TPMS-gyroid catalyst increased to 54.7 MPa, which is 15.3 times greater than that of the as-printed structure (compressive strength of 3.57 MPa). The compressive strength of the Spinodoid-iso catalyst reached 9.97 MPa, which is 11.1 times greater than that of the as-printed structure (compressive strength of 0.90 MPa). Notably, the fracture strain of the Spinodoid-iso catalyst reached 19.9%. Three-point bending tests revealed that the sintered TPMS-gyroid and Spinodoid-iso structures achieved bending strengths of 55.49 and 47.98 MPa, respectively, substantially exceeding that (20.15 MPa) of the square lattice. Similarly, fracture tests showed that the sintered TPMS-gyroid and Spinodoid-iso exhibited higher fracture toughness (2.51 and 1.56 MPa·m^1/2^, respectively) than the square architecture (0.71 MPa·m^1/2^). Together with the strengthening effect of the sintered ceramic backbone, these results indicate that shell-based architectures enhance both load-bearing capacity and resistance to flexural deformation and crack propagation.

To investigate the influence of catalyst architectures on transport performance, we performed CFD simulations and further analyzed fluid flow behaviors in the square lattice, TPMS, and spinodoid catalyst frameworks. Conventional pellet catalysts, being solid throughout without internal pore channels, have negligible internal surface area and were therefore excluded from the fluid dynamics simulations. In CFD simulations, the laminar flow model was used, and water was treated as the continuous phase. Velocity and shear velocity distributions were calculated for each framework at a relative density of 30%. Figure 3 (B, E, and H) and fig. S12 show the fluid velocity distributions in the vertical cross sections of various catalyst frameworks with a relative density of 30%. The square lattice exhibited concentrated high-velocity flow in the center of the channel due to its large pore size and flat structure, whereas the TPMS architectures, featuring curved surfaces and refined internal pore sizes, demonstrated a relatively dispersed fluid velocity distribution, which facilitated fluid mixing and enhanced pollutant transport to the catalyst surface (*14*, *41*). The Spinodoid-iso structure, with its inherently disordered porosity and high surface area, showed the most diffused flow pattern and substantially reduced flow velocity with the longest residence time (figs. S13 to S15). These features mitigated the formation of fast-flowing central channels, thereby enabling more uniform fluid-catalyst interaction across the framework. Shear velocity analysis, shown in Fig. 3 (C, F, and I) and fig. S16, revealed that both TPMS and spinodoid structures generated significantly higher shear rates than the square lattice, particularly near the fluid-solid interfaces. Such high shear rates facilitate sufficient pollutant-site contact and mass transfer efficiency, creating a favorable microenvironment for the catalytic reaction. Volume-averaged shear velocities, calculated across all structures at 30% relative density (fig. S17), further confirmed this trend, with TPMS and spinodoid frameworks exhibiting shear rates more than twice those of the square lattice. Notably, the prolonged residence time increases the overall frequency of pollutant-surface encounters, while the elevated interfacial shear enhances the mass transfer efficiency of each interaction. These two effects act synergistically, boosting the total reactant flux to the catalyst surface and reinforcing every stage of the transport-reaction process, thereby further improving catalytic utilization. These results underscore the crucial role of continuous 3D porous curvature in regulating local hydrodynamics and optimizing mass transport behavior, thereby enhancing the utilization of catalytic active sites.

To quantitatively compare the mass transfer characteristics of different catalyst architectures, we calculated their permeability (*k*) based on pressure drop (Δ*P*) using Darcy’s law. Figure 3J and fig. S18 illustrate the computational permeability and pressure distribution for catalysts with different architectures at a fixed relative density of 30%, respectively. Overall, all TPMS and spinodoid frameworks exhibited higher pressure drops and lower permeabilities than the square lattice, reflecting more tortuous internal flow paths. Among them, the TPMS-Neovius framework showed the highest pressure drop (79.63 Pa) due to its ultrafine pore channels, resulting in the lowest permeability. While such constricted pores may increase local fluid velocity, they also risk impeding flow under real wastewater conditions due to capillary effects and surface tension. Notably, both TPMS-gyroid and Spinodoid-iso showed comparable pressure drops and computational permeabilities (8.78 × 10^−9^ and 7.88 × 10^−9^ m^2^, respectively), both of which were much lower than those of the square lattice (5.10 × 10^−8^ m^2^). In anisotropic spinodoid designs, differences in horizontal and vertical pore alignment led to architectural-dependent variations in permeability: Spin-lam exhibited reduced permeability, whereas Spin-col showed enhanced values. In contrast, isotropic frameworks such as TPMS-gyroid provided uniform flow resistance.

Permeability was also found to be strongly dependent on porosity. As relative density increased, permeability consistently decreased across all structures (fig. S19). Using spinodoid frameworks as an example, increasing relative density led to narrower pores and elevated pressure drops, which, in turn, elevated flow velocities and shear rates near the liquid-solid interfaces (figs. S20 to S22). Figure 3K presents the relationship between specific surface area and permeability across architectures. At comparable specific surface areas, TPMS-gyroid and Spinodoid-iso frameworks achieved permeability values closely matching those of human cancellous bone (5.6 × 10^−9^ ∼ 8.3 × 10^−9^ m^2^) (*42*). This biomimetic alignment suggests that such architected structures support continuous water flow while maintaining intimate contact with the framework surfaces. In addition to the single-phase laminar flow simulations discussed above, we further investigated the effect of a gas phase on the trends observed in different catalyst architectures. Given the intrinsic solid-gas-liquid three-phase nature of catalytic ozonation, a laminar bubble flow model was performed to assess the impact of gaseous ozone. The simulation results indicated that, under the ozone flux used in this study, the ozone bubbles had no significant influence on the fluid velocity, shear velocity, and pressure distribution (text S3). Therefore, the enhanced mechanical properties, increased interfacial shear velocity, and reduced permeability in architected frameworks synergistically enhance structural integrity and pollutant-site interaction, unattainable in other conventional supported catalysts. These synergistic effects lay the structural foundation for efficient mass transfer and catalytic component utilization.

### Water purification performance in synthetic and real wastewater

**Fig. 4.**
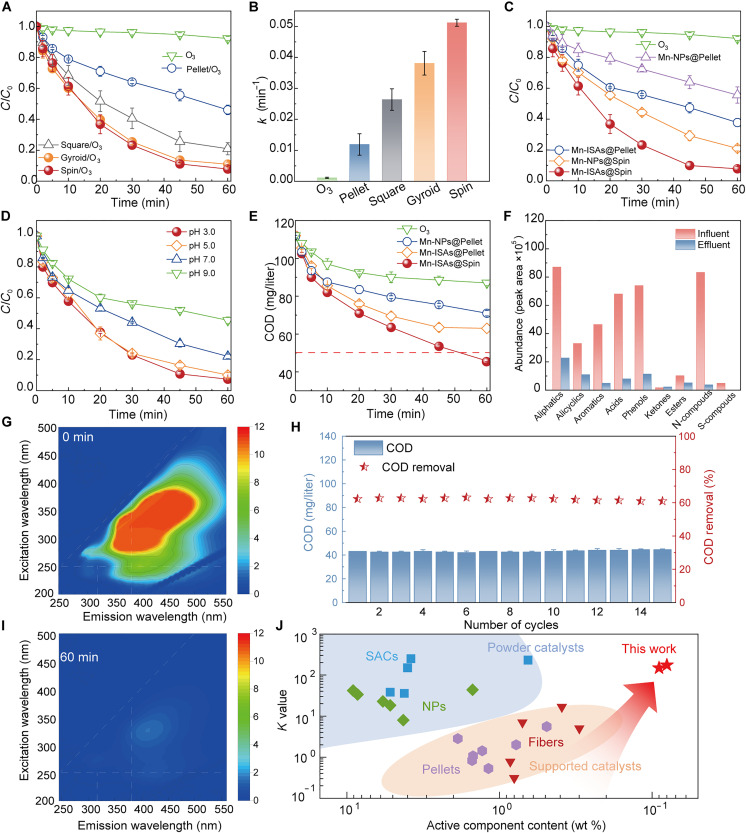
Water purification performance of Mn-ISAs@AAF catalysts and comparison. (**A** and **B**) OA degradation performance and corresponding kinetic constants of different Mn-ISAs@AAF catalysts. (**C**) OA degradation performances of different catalysts. (**D**) Degradation efficiency at different initial pH conditions. (**E** and **F**) COD degradation and changes in the organic pollutant content of actual coal-chemical wastewater. (**G** and **I**) Excitation emission matrix (EEM) spectra of actual coal chemical industry wastewater before (G) and after (I) treatment. (**H**) Cycling tests for the Mn-ISAs@Spinodoid catalyst. (**J**) Comparison of *K* value and active component content for different types of catalysts.

Owing to the optimal configurations of the AAFs and the atomically dispersed reactive sites, the Mn-ISAs@AAF exhibited superior performance in the heterogeneous catalytic ozonation (HCO) process for highly efficient water purification. These advantages were first demonstrated through model contaminant degradation. Oxalic acid (OA) was selected as the representative organic contaminant to evaluate the catalytic performance because it is a refractory pollutant and has a low reaction rate with ozone (*43*). Figure 4 (A and B) demonstrated the degradation performance of different structures with identical Mn-ISAs content. Mn-ISAs@Spinodoid-iso (Mn-ISAs@Spinodoid for short) and Mn-ISAs@TPMS-gyroid (Mn-ISAs@Gyroid for short) achieved OA degradation over 90%, far surpassing the performance of the square lattice structure, conventional solid alumina pellets, and the limited 8% degradation by ozone alone, underscoring the importance of architected frameworks in enhancing performance. Among all frameworks, Mn-ISAs@Spinodoid also demonstrated the best absorption properties and highest specific surface area, which was favorable for the removal of OA on the catalyst surface (fig. S23). The normalized kinetics evaluated through a pseudo-first-order reaction model demonstrated that the removal efficiency gradually improved from Mn-ISAs@Square to Mn-ISAs@Spinodoid. This trend aligned well with the computational mass transfer analysis (Fig. 3K), confirming that the combination of high shear velocity, appropriate permeability, and large accessible surface area collectively governs catalytic performance under identical reactive site loadings (fig. S24). Notably, the same structure-dependent performance trend was also observed for Mn-free bare AAF and CN@AAF during ozonation (figs. S25 and S26). This indicates that architectural topology intrinsically governs mass transfer–related performance differences, while Mn incorporation uniformly elevates the overall catalytic activity.

To further reveal that the improvement in catalytic activity was attributed to both the designed structures and atomically dispersed sites, conventional Mn-loaded catalysts with nanoparticle clusters were synthesized for comparison with the chosen Spinodoid-iso structure. As shown in Fig. 4C, compared with all the other catalysts, Mn-ISAs@Spinodoid exhibited the best performance for OA degradation. Notably, the rate constant (*k*_obs_) (0.0512 min^−1^) of Mn-ISAs@Spinodoid increased 4.56-fold compared with that of Mn-NPs@Pellet (0.0092 min^−1^, fig. S27), which was a general combination of active metal components and substrates. Regardless of the support architecture (Spinodoid or pellet), the low-loading Mn-ISAs exhibited superior performance to the high-loading Mn-NPs. This trend underscores that the unique chemical coordination and electronic structure of the single-atom sites are inherently more active for ozonation process. Meanwhile, neither intrinsic site activity enhancement nor mass transfer regulation alone can account for the overall performance improvement; instead, their coupling leads to a synergistically enhanced performance (fig. S28). To provide a broader benchmark beyond pellets, the performance of Mn-ISAs@AAF was also compared against a typical fiber-based catalyst, further underscoring its architectural advantages (fig. S29). The Mn-ISAs@Spinodoid with the best performance was selected for further experiments. Moreover, Mn-ISAs@Spinodoid exhibited higher reactivity than its homogeneous counterpart (Mn^3+^/O_3_ system) even under equal Mn dosage (fig. S30), and its performance advantage remained when controlling for equal catalyst volume (fig. S31). Meanwhile, the Mn-ISAs@Spinodoid maintained robust activity across a wide pH range (*3*–*9*), achieving high degradation under acidic and neutral conditions and still maintaining 60% removal in alkaline solution (pH 9) after 60 min (Fig. 4D), with negligible Mn leaching throughout the process (fig. S32). These results highlight that the Mn-ISAs@AAF catalysts offer outstanding reactivity, enabled by the synergistic interplay between bioinspired architectures and atomically dispersed active sites.

Beyond model pollutant removal, Mn-ISAs@Spinodoid also exhibited outstanding advanced degradation for actual coal-chemical wastewater, which is a typical refractory organic industrial wastewater with a chemical oxygen demand (COD) of ∼110 mg liter^−1^. As shown in Fig. 4E, the Mn-ISAs@Spinodoid presented the highest removal efficiency, and the effluent COD decreased to 43 mg liter^−1^ (fig. S33 and table S5). These results also indicated that the coexisting suspended solids and salinity in the real wastewater exerted negligible adverse effects on the catalytic performance, confirming the excellent anti-interference ability under complex matrix conditions. In contrast, the COD outcome of pure ozonation was 87 mg liter^−1^ and that of the general Mn-NPs@Pellet was 71 mg liter^−1^. The dissolved organic matter content of coal-chemical wastewater and the water quality before and after treatment were analyzed thoroughly. The gas chromatography–mass spectrometry (GC-MS) results (Fig. 4F) revealed that, after catalytic ozonation by the Mn-ISAs@Spinodoid catalysts, the abundance of organic compounds in the effluent markedly decreased. Furthermore, 3D excitation emission matrix (EEM) fluorescence spectra were used to analyze the sources and intensities of different organic components in the influent and effluent through the fluorescence region integration method. As shown in Fig. 4 (G and I) and fig. S34, almost all the fluorescent macromolecules were removed after 60 min of treatment, further indicating their effectiveness in coal-chemical wastewater treatment, which is consistent with the results of the UV-visible (UV-vis) spectrum (fig. S35). Consequently, the high removal efficiency, broad-spectrum pollutant degradation, and exceptional reusability of Mn-ISAs@Spinodoid in complex wastewater matrices demonstrate its strong potential for practical implementation in large-scale industrial water purification systems.

To further demonstrate the universality and stability of Mn-ISAs@Spinodoid, the degradation tests using other types of industrial wastewaters and cyclic experiments were conducted. Two other representative industrial wastewaters were selected, petrochemical wastewater and pharmaceutical wastewater, to further verify the universality of the bioinspired architected catalyst. As shown in fig. S36, after 60 min of degradation, the effluent COD concentrations of both wastewaters decreased to below 50 mg liter^−1^. The 3D EEM fluorescence spectra revealed that most of fluorescent organic species disappeared, indicating substantial pollutant degradation. These results, together with the degradation of coal-chemical wastewater, indicated that the Mn-ISAs@AAF catalyst exhibits broad applicability across various types of industrial wastewater. Moreover, the cyclic degradation experiments using actual coal-chemical wastewater were supplemented. The catalyst was continuously operated for 15 successive cycles, and, in each cycle, the effluent COD remained consistently below 50 mg liter^−1^, demonstrating the excellent reusability (Fig. 4H). Meanwhile, the pressure drop remained stable under different wastewater concentrations and long-term operation, further confirming its excellent anti-fouling capability and long-term operational stability (fig. S37). This indicated that, under prolonged hydraulic erosion and flow conditions, the porous structure of the catalyst did not suffer clogging or collapse, with internal flow channels remaining unobstructed, demonstrating outstanding hydrodynamic and structural stability. Characterizations after long-term operation were also performed. As shown in the postreaction SEM images (fig. S38), the shell-based architectures remained intact without obvious cracking or pulverization. Furthermore, the compressive strength and bending strength of all three architectures remained essentially unchanged after continuous hydraulic flushing (fig. S39). TEM characterizations further revealed that the dispersion of active sites remained essentially stable, demonstrating that the interfacial bonding between the carbon layer and the alumina support is preserved after multiple reaction cycles. Postreaction N 1s XPS spectra (fig. S40) revealed that the Mn-N*_x_* coordination environment remained stable after reaction, while the observed evolution in N-functionalities was consistent with known oxidative restructuring of the N-doped carbon support (*44*). The above findings confirmed that the Mn-ISAs@AAF catalysts have remarkable structural stability, mechanical robustness, and catalytic durability under long-term hydraulic erosion.

Figure 4J presents a comparative analysis of Mn-ISAs@AAF catalysts against reported systems in terms of catalytic efficiency (*K* value) and metal utilization (active component content). All relevant data are included in table S6. Our architected catalyst occupies the previously unattained region in Fig. 4J for nearly all powder and supported catalysts. Powder catalysts typically exhibit high reactivity due to full exposure of active sites, resulting in excellent *K* values; however, they suffer from poor mechanical strength and limited recyclability, which hinders their practical deployment. In contrast, supported catalysts exhibit improved reusability and structural stability with minimal active metal usage, resulting in high active component utilization; yet, their catalytic activity is generally limited due to mass transfer constraints imposed by rigid support frameworks. Achieving an optimal balance between these attributes has been a long-standing challenge as nearly all reported catalysts exhibited the inherent trade-offs. Although some attempts have been made to bridge this gap, they remain limited. Notably, our Mn-ISAs@AAF catalysts transcend this dichotomy by simultaneously achieving exceptional catalytic activity and efficient metal utilization, combining high catalytic efficiency with ultralow Mn loading (0.08 wt %). The exceptional performance of Mn-ISAs@AAF underscores the significance of overcoming traditional scale-dependent optimization limitations, illustrating that synergistic coupling of macroscale shell-based architectures and atomic dispersion can fundamentally advance reactivity, reusability, and catalytic component utilization synergistically beyond conventional limits.

### Catalytic mechanisms and structural synergy in architected catalysts

**Fig. 5.**
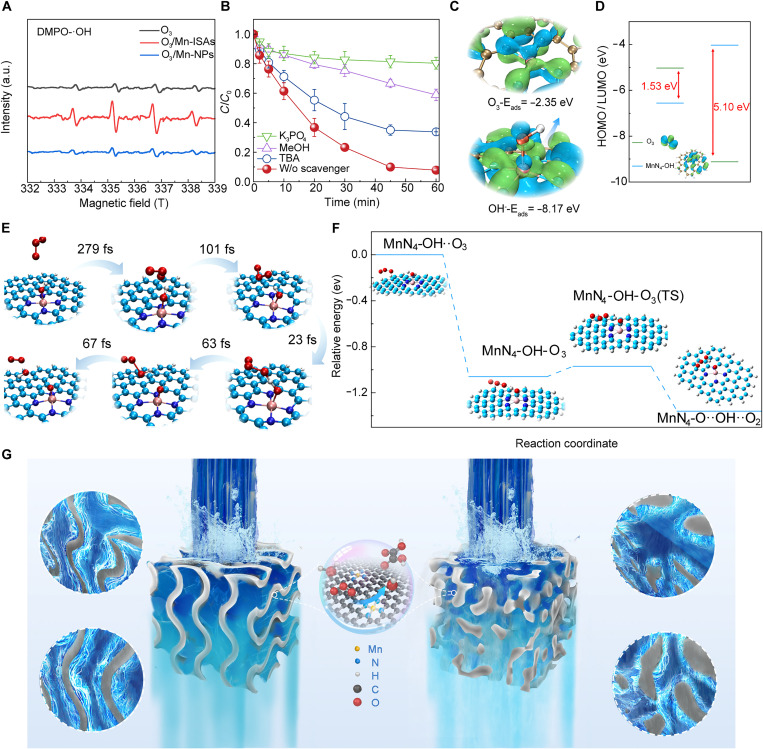
Reactive species identification and DFT calculations for elucidating the catalytic mechanism. (**A**) DMPO-trapped EPR spectra of different systems. (**B**) Quenching tests for OA in the O_3_/Mn-ISAs system. (**C**) O_3_ and OH^−^ adsorption energy on MnN_4_ sites. (**D**) Lowest unoccupied molecular orbital (LUMO)–highest occupied molecular orbital (HOMO) analysis between MnN_4_-OH and O_3._ (**E**) Dynamic process simulated by AIMD in the formation of •OH. (**F**) Relative energy profile. (**G**) Multiscale-integrated mechanism in bioinspired architected catalysts.

Given the outstanding performance of Mn-ISAs@AAF, arising from the integration of advantages associated with both powder and supported systems, it is imperative to further investigate the underlying catalytic mechanisms at the atomic level, as well as the structural synergy across scales. Hydroxyl radicals (•OH) were identified as the predominant ROS responsible for the superior catalytic ozonation performance observed with the Mn-ISAs@AAF catalysts. Electron paramagnetic resonance (EPR) spectroscopy using 5,5-dimethyl-1-pyrroline *N*-oxide (DMPO) as the spin-trapping agent indicated enhanced •OH generation in the Mn-ISAs@AAF system compared to control systems (Fig. 5A). Specifically, the characteristic quadruple peaks of DMPO-•OH (hyperfine splitting constants *A*_N_ = _Hβ_ = 14.78 G) were significantly more pronounced in the Mn-ISAs@AAF reaction, confirming the critical role of atomically dispersed Mn-N_4_ sites in promoting radical production. In addition, the generation of superoxide radical anion (•$O_{2}^{-}$), an essential intermediate for initiating radical chain reactions, was also notably enhanced (fig. S41), further supporting the central role of •OH in pollutant removal (*45*).

To verify the contribution of •OH radicals more explicitly, radical scavenging tests were conducted. The introduction of tert-butanol (TBA; a bulk-phase •OH scavenger) and methanol (MeOH; scavenging both bulk and surface-bound •OH) significantly suppressed OA degradation (Fig. 5B). The OA removal rate decreased from 92.2 to 66.2% and 41.3% after adding TBA and MeOH, respectively. The results indicated that •OH played an important role in the degradation of pollutants, both in the solution and on the catalyst surface. Fluorescence images of the Mn-ISAs@AAF system further confirmed the generation of surface-absorbed •OH (fig. S42). When the concentration of MeOH was increased from 10 to 15 mM, the change in OA removal was quite small (fig. S43). To further explore the original sites of ROS generation, phosphate (K_3_PO_4_) was used as a Lewis base to demonstrate the importance of surface hydroxyl groups on the catalysts for catalytic ozonation. Phosphate is a stronger Lewis base than water and can replace the surface hydroxyl groups on the catalyst, reducing the number of surface hydroxyl groups and ultimately hindering the activation process of ozone to radicals (*46*). After adding 15 mM K_3_PO_4_, OA removal was significantly inhibited, with only 20% OA degraded through the reaction, indicating the importance of surface hydroxyl groups in the generation of ROS (such as •OH) during the degradation process.

The atomic dispersion of Mn-N_4_ sites specifically promotes •OH generation via a two-step mechanism, facilitating the decomposition of O_3_ through a low-energy pathway that directly yields •OH radicals. Ab initio MD (AIMD) simulations and DFT calculations are two widely used methods for elucidating mechanisms. AIMD is used to simulate the dynamic reaction process, whereas DFT is applied to precisely calculate reaction energy barriers (*47*, *48*). The Mn-N_4_ configuration was identified as the dominant coordination environment of the Mn single atoms based on the preceding characterization results. The electrostatic potential (ESP) maps of O_3_, OH^−^, and the carbon skeleton containing the Mn-N_4_ moiety (fig. S44) indicated that Mn single-atom site with low electron density was susceptible to attack by electron-rich groups. The adsorption energies of O_3_ and OH^−^ were calculated by DFT calculations. As shown in Fig. 5C, the adsorption energy of OH^−^ was much lower than that of O_3_, meaning that it was easier to form Mn-OH structures at single-atom sites. These results aligned with previous studies (*49*, *50*), indicating that transition metal atomic sites were very easily occupied by hydroxyl groups to form the M-OH structure when surrounded by water molecules. Figure S45 shows a sequence of snapshots of the dynamic process of OH^−^ interactions with the Mn-N_4_ carbon skeleton from AIMD simulations. These AIMD results verified the reactive sites calculated above. The simulation animation illustrates the formation of the MnN_4_-OH intermediate in the first step (movie S1): During the dynamic interaction process, OH^−^ gradually approached Mn-N_4_ due to electrostatic effects, ultimately forming a stable configuration after 105 fs. Therefore, the Mn-N_4_ sites occupied by hydroxyl groups (MnN_4_-OH) tended to become the reactive sites for the generation of ROS.

•OH was continuously produced during the reaction between ozone and the carbon skeleton with MnN_4_-OH sites. Molecular orbital theory can be used to describe the state of electrons in polyatomic molecules and analyze possible reaction paths. As shown in Fig. 5D, a lower gap between the highest occupied molecular orbital of MnN_4_-OH and the lowest unoccupied molecular orbital of ozone was calculated, indicating that the MnN_4_-OH sites had a higher reactivity for attacking ozone molecules as electron donors. The entire reaction between MnN_4_-OH and O_3_ was also simulated by AIMD to demonstrate the intermediate states of the reaction (Fig. 5E and movie S2). HO_3_• was progressively generated throughout the process, playing important roles in •OH initiation, which was consistent with previous studies (*50*, *51*).

On the basis of the calculated Mulliken charges and spin population, the active oxygen species with a charge of −0.28|*e*| and a spin density of 0.58 was more likely to be a •OH radical, whereas the active oxygen species left on the surface with 0.60 spin population might be surface atomic oxygen (*O) (*52*), which can further transform into •OH. The relative energy changes of the reaction process were also calculated to demonstrate the thermodynamic properties. As shown in Fig. 5F, the formation of intermediate HO_3_• represented a critical transition state (TS) in the entire reaction, serving as a potential limiting step for the subsequent generation of ROS. The entire process was exothermic and spontaneous, which was favorable for the generation of •OH, consistent with the aforementioned characterization and experimental results.

Based on the above analyses, the Mn-ISAs@AAF catalyst successfully integrates key functionalities across multiple structural levels (Fig. 5G, created by Autodesk Maya and Adobe Photoshop). The improved catalytic performance originates from the intrinsic synergy between the shell-based architecture and the Mn-N_4_ sites rather than from either component alone. In practical catalytic ozonation systems, degradation mechanisms generally include fouling, pore blockage, and bed compaction from accumulated organic/inorganic species (*53*, *54*), hindering mass transfer. Our bioinspired architecture, with open channels and high interfacial shear, mitigates deposition and compaction, substantially retarding these dominant degradation pathways and prolonging catalyst service life. The architected framework regulates permeability, interfacial shear, and active-site exposure, thereby determining pollutant-site contact and mass transport behavior. At the same time, the atomically dispersed Mn-N_4_ sites can spontaneously generate key reactive intermediates and avoid the harsh localized oxidative environments, promoting the overall framework to maintain stable operation over repeated ozonation cycles. This complementary interaction across structural and catalytic domains underlies the simultaneous improvement in activity, durability, and efficient metal utilization.

## DISCUSSION

In this work, we developed a bioinspired architected catalyst with single-atom Mn through the synergistic integration of high-precision 3D printing and ultrasonic decoration with single Mn atoms, enabling a precisely regulated multiscale architecture for highly efficient water purification. The bioinspired shell-based framework not only enhances mechanical strength via relieving local stress concentration but also orchestrates the catalytic microenvironment by improving mass transfer and interfacial dynamics, thereby synergistically maximizing the utilization efficiency of the atomic sites. Furthermore, atomically dispersed Mn-N_4_ sites were successfully constructed on the architected framework to facilitate the formation of key reactive intermediates (HO_3_•), thereby significantly accelerating •OH generation under mild conditions conducive to long-term structural integrity. With minimal active component loading, the catalyst demonstrated outstanding performance in HCO for both synthetic and real wastewater. Multiscale modeling and simulations corroborated the synergy mechanisms: CFD simulations decoded how the architecture governs transport, while DFT and AIMD simulations revealed the atomic-scale reaction pathway. The fabrication cost of the bioinspired architected catalyst is expected to be mitigated by evolving large-scale 3D printing technologies, further enhancing its economic viability for practical water purification. Thus, this work transcends the limitations of both powder and supported catalysts by synergistically integrating high degradation efficiency, long service life, low material consumption, and structural scalability into a unified multiscale synergistic system for water purification, offering a general design principle applicable to a wide range of catalytic systems.

## MATERIALS AND METHODS

### Chemicals and materials

Zinc nitrate hexahydrate [Zn(NO_3_)_2_•6H_2_O] and OA dihydrate were obtained from Sinopharm Chemical Reagent Co. Ltd. (China). *p*-Hydroxybenzoic acid, 2-methylimidazole (C_4_H_6_N_2_), Mn(III) acetylacetonate [Mn(acac)_3_; C_15_H_21_MnO_6_], MeOH, and TBA of analytical grade or better were purchased from Beijing J&K Scientific Co. Ltd. (China). DMPO and 2,2,6,6-tetramethyl-4-piperidinol were received from Shanghai Meryer Chemical Technology Co. Ltd. (China). All reagents were used without further purification.

### Fabrication of 3D AAF

The DLP printing of 3D AAFs was performed using a PμSL-based 3D printer (microArch S240, BMF, China) with a resolution of 10 μm. The required architectures, including square, TPMS, and spinodoid frameworks, were sliced using Materialise Magics software. Alumina resin (CA-100A, BMF, China) was poured into the printer’s container. A 405-nm UV beam with microstructural patterns selectively cured the alumina resin on the platform surface. The printing layer thickness was set as 20 μm. The complete set of printing parameters is summarized in table S7. After printing, the structures underwent ultrasonic cleaning and water rinsing to remove residual resin. Following natural air drying for 24 hours, heat treatment for debinding and sintering was performed according to the temperature curve shown in fig. S46.

### Synthesis of single-atom Mn active sites

Typically, a certain amount of Mn(acac)_3_ (0.705 g, 2 mmol) was dissolved in 50 ml of MeOH containing zinc nitrate (2.98 g, 10 mmol) and was stirred for 10 min to prepare solution A. Similarly, 3.29 g of C_4_H_6_N_2_ was dispersed into 30 ml of MeOH to obtain solution B. At normal temperature and pressure, solution A was quickly poured into solution B containing the catalyst supports (AAFs, pellets, and fibers), and the mixture was then put into an ultrasonic reactor for 1-hour reaction. The resulting catalysts were filtered out, washed three times with ethanol, and dried at 65°C under vacuum overnight. The dried materials were calcined at 900°C in a tube furnace for 3 hours under the argon atmosphere to obtain the Mn-ISAs@AAFs. It is noted that, during high-temperature treatment, the Zn^2+^ nodes in ZIF-8 are reduced to metallic zinc, which subsequently vaporizes and escapes from the framework, thereby being effectively removed from the final catalyst (*55*, *56*). For comparison, the amount of Mn(acac)_3_ added was 0 and 4.2 g to obtain the CN@AAFs and Mn-NPs@AAFs (table S8).

### Material characterization

SEM observations were conducted on ZEISS Gemini SEM 500. A transmission electron microscope (TEM, JEOL-2010F) equipped with an energy-dispersive spectrometer (INCA-IET 200) was used to obtain TEM and high-resolution TEM images and EDS maps of the catalysts. The HAADF-STEM images were collected by an FEI Titan 80-300 instrument. Raman spectra were characterized by a LabRAM HR Evolution spectrometer with a 532-nm laser. XRD patterns were obtained using a D/max-2550 diffractometer with Cu Kα radiation. The XPS measurements were conducted using an ESCALAB 2502Xi instrument (Thermo Fisher Scientific). The Mn K-edge XAS spectra were collected at beamline 12-BM-B at the Advanced Photon Source at Argonne National Laboratory using double-crystal monochromator. The Mn loading contents and leaching concentrations in the influent were detected by inductively coupled plasma–mass spectrometry (Agilent 7800). The signals of different ROS were detected by EPR tests on a JEOL FA-200 spectrometer.

### Catalytic ozonation measurements

Catalytic ozonation experiments were carried out in a self-made acrylic reactor at the bench scale. OA was selected as the model contaminants, while an actual coal-chemical wastewater was selected as the typical refractory organic wastewater. The catalysts were added to 300 ml of wastewater solution, and the ozone generated from an ozone generator (Longevity EXT120, Canada) was quickly introduced to initiate the catalytic reactions at room temperature. Detailed degradation parameters are as follows. The OA degradation: The concentration was 200 mg liter^−1^; at the same mass, the dosage of different catalysts was 4 g, while, at the same volume, the dosage of pellet catalyst was 6.5 g and the others were still 4 g, the O_3_ concentration was 5 mg liter^−1^, and the O_3_ flow rate was 100 ml min^−1^. The actual coal-chemical wastewater degradation: The influent COD was about 110 mg liter^−1^, the dosage of catalysts was 4 g, the O_3_ concentration was 10 mg liter^−1^, and the O_3_ flow rate was 500 ml min^−1^. Different scavengers (TBA, MeOH, *p*-benzoquinone (*p*-BQ), and K_3_PO_4_) were used for quenching experiments. The reaction solution was sampled at fixed intervals. The OA was detected by high-performance liquid chromatography (Agilent 1200), while the coal-chemical wastewater was measured with a COD detector (5B-3BV8, LianHua). The water qualities of coal-chemical wastewater were analyzed by 3D EEM fluorescence spectroscopy (F-7000, Hitachi, Japan), UV-vis spectrophotometer (Hach, DR6000) and GC-MS (7890A-5975C, Agilent). All catalytic ozonation experiments were performed in triplicate using independently prepared samples, and the averaged values with SDs were reported.

### Mechanical testing

Uniaxial compression tests were performed on the 3D AAFs to evaluate their mechanical properties. The tests were conducted using a commercial universal material testing machine (AGX-V2, Shimadzu Corporation, Japan) equipped with a 20-kN load cell. The compression strain rate was set as 10^−3^ s^−1^. Stress was calculated by dividing the applied force by the initial cross-sectional area of the alumina frameworks.

Square, TPMS-gyroid, and Spinodoid-iso rectangular bars with the same characteristic feature size as the 3D AAFs were printed, with dimensions of 35 mm by 5 mm by 5 mm and a relative density of 30%. The printed specimens were subjected to debinding and sintering by following the same postprocessing protocol described above. After sintering, the bars shrank to 28.84 mm by 4.12 mm by 4.12 mm. Three-point bending tests were then performed on the sintered specimens using a universal testing machine with a 20-kN load cell (AGX-V2, Shimadzu Corporation, Japan). A span length of 25 mm was used, and the loading head applied displacement at a constant rate of 0.3 mm min^−1^. Force-displacement curves were recorded for each specimen, and the maximum load before fracture was taken as the failure load *P*. The bending strength σ*_f_* was calculated using the following expression

$$\sigma_{f}=\frac{3\mathrm{PL}}{2bd^{2}}$$ (1)

where *L* is the span length and *b* and *d* are the specimen width and height, respectively.

Compact tension (CT) fracture specimens of square, TPMS-gyroid, and Spinodoid-iso were printed. The overall dimensions of the as-printed CT specimens were designed on the basis of ASTM E1820 (*57*), with *W* = 15 mm and *B* = 7.5 mm. A precrack with length of *a*_0_ = 4.5 mm and height of 0.1 mm was incorporated into the sliced images and directly printed. The printed specimens were subjected to debinding and sintering. After sintering, the CT specimens shrank to final dimensions of 15.45 mm by 14.83 mm by 6.18 mm with *W* = 12.36 mm and *B* = 6.18 mm. Fracture tests were then performed on the sintered CT specimens using a universal testing machine with a 20-kN load cell (AGX-V2, Shimadzu Corporation, Japan) at a constant displacement rate of 0.3 mm min^−1^. Force-displacement curves were recorded for each sample. Fracture toughness was evaluated in terms of the mode-I stress intensity factor *K*_I_, following the procedure outlined in ASTM E1820. The stress intensity factor at an instant (*i*) was calculated from the recorded force *P*_(*i*)_ as

$$K_{I\left(i\right)}=\frac{P_{\left(i\right)}}{B\sqrt{W}}f\left(\frac{a_{\left(i\right)}}{W}\right)$$ (2)

where *a*_(*i*)_ is the crack length at the instant (*i*) and *f*(*a*_(*i*)_/*W*) is the calibration function for CT specimens, given by

$$f\left(\frac{a_{\left(i\right)}}{W}\right)=\frac{\left(2+\frac{a_{\left(i\right)}}{W}\right)\left[0.886+4.64\left(\frac{a_{\left(i\right)}}{W}\right)-13.32{\left(\frac{a_{\left(i\right)}}{W}\right)}^{2}+14.72{\left(\frac{a_{\left(i\right)}}{W}\right)}^{3}-5.6{\left(\frac{a_{\left(i\right)}}{W}\right)}^{4}\right]}{{\left(1-\frac{a_{\left(i\right)}}{W}\right)}^{\frac{3}{2}}}$$ (3)

It is noted that the present sintered ceramic lattices exhibit brittle fracture behavior with negligible plastic deformation. For such brittle materials, the critical stress intensity factor *K*_IC_ is commonly used as the primary measure of fracture toughness. Therefore, the calculated *K*_I_ values at failure were taken as *K*_IC_. For each type of architecture, the compression tests, bending tests, and fracture tests were performed on three independent samples.

### CFD finite element simulations

The CAD models of TPMS and Spinodoid geometries were constructed and then imported into COMSOL Multiphysics 6.2 software to solve the continuity and Navier-Stokes equations. Water was used as the flow medium, with a density of 1000 kg/m^3^ and a viscosity of 1.01 mPa·s. To ensure laminar flow within the entire catalyst, an inlet velocity of 10^−3^ m/s was applied at the inflow side, and the outflow pressure was set as 0. A fluid domain with a height equal to half of the catalyst height was introduced above the catalyst to avoid the boundary effect, and the sides of the catalyst were defined as walls. The fluid velocity, shear velocity, and pressure distributions were obtained using a stationary solver. The particle tracing module was used to simulate tracer-particle trajectories within the laminar flow velocity field. In this model, 500 massless tracer particles were uniformly released from the inlet plane and tracked over a total simulation time of 20 s using a time-dependent solver. The particles were coupled with the laminar velocity field under a steady state, indicating that they serve as tracers within the fluid rather than representing solid particles. The average pressure at the inlet and outlet planes of the catalyst was extracted and used in the Darcy equation to determine the computational permeability (*58*)

$$k=\frac{v\mu H}{\mathrm{\Delta P}}$$ (4)

where *v* is the fluid velocity, μ is the dynamic viscosity coefficient, *H* is the height of model, and Δ*P* is the pressure difference between the top and bottom surfaces of the structure, defined as Δ*P* = *P*_in_ − *P*_out_.

### DFT calculations

DFT calculations were carried out using the Gaussian package. The Becke-Lee-Yang-Parr (B3LYP) functional with the 6-31+G(d) basis set were applied to optimize the configurations of the reactants, TSs, and products. Frequency calculations were carried out to confirm the stability of all structures, and solvent effects were also considered. The TS configurations were further confirmed by one imaginary vibration frequency. Meanwhile, the formation energy (*E*_f_) of Mn-N*_x_*C*_y_* and the adsorption energies (Δ*E*_ads_) of reactants on the active sites were calculated (details were in text S4).

### MD simulations

AIMD was used to simulate the dynamic interactions between the reactants and active sites by the CP2K package, with the Quickstep method and the basic set of MOLOPT. The core electrons were described by the norm-conserving Goedecker-Teter-Hutter pseudopotentials. The valence electron wave functions were extended in the double-zeta plus polarization basis sets. The SCF was based on the traditional RESTART diagonalization algorithm with the convergence criterion of 1.0 × 10^−6^ atomic units (a.u). The simulations were accomplished in the canonical ensemble with a constant number of particles, volume, and temperature (NVT), using Nose-Hoover thermostats with a time step of 0.5 fs at a finite temperature of 298 K.

## Supplementary Materials

### The PDF file includes:

Supplementary Text S1 to S4 Figs. S1 to S52 Tables S1 to S8 Legends for movies S1 and S2 References
