# Supplementary material for: Bioinspired architected catalyst for efficient water purification: Bridging reactivity, reusability, and catalytic component utilization
Source: Sci Adv. 2026 Aug 5;12(32):eaeb0624. doi: 10.1126/sciadv.aeb0624 (PMC13440438; doi:10.1126/sciadv.aeb0624)
Supplement: Supplementary file 1 [file sciadv.aeb0624_sm.pdf]

Science Advances
AAAS

Supplementary Materials for

Bioinspired architected catalyst for efficient water purification: Bridging reactivity, reusability, and catalytic component utilization

Xirui Zhou *et al.*

Corresponding author: Xiaoyuan Zhang, zhangxiaoyuan@tsinghua.edu.cn;
Xiaoyan Li, xiaoyanlithu@tsinghua.edu.cn

Sci. Adv. **12**, eaeb0624 (2026)
DOI: 10.1126/sciadv.aeb0624

The PDF file includes:

Supplementary Text S1 to S4
Figs. S1 to S52
Tables S1 to S8
Legends for movies S1 and S2
References

Other Supplementary Material for this manuscript includes the following:

Movies S1 and S2

Text S1. Details of design strategy of TPMS and spinodoid

Triply Periodic Minimal Surface (TPMS) is the surface with a periodic minimum area in three directions in space for a given boundary with zero curvature at each point (*59*). These structures can be described using a level-set approximation equation, $\phi(x, y, z)$, which incorporates trigonometric functions (*59*). The minimal surface-like topology is defined by evaluating this function at an isovalue, i.e., $\phi(x, y, z) = c$. For lattice structures with finite wall thickness, TPMS is derived from the region bounded by two surfaces with distinct level-set values, i.e., $\phi(x, y, z) = \pm c$. The constant c governs both the relative density and wall thickness. Table S1 lists several common TPMS lattices with finite wall thicknesses, along with their corresponding level set equations, including gyroid, IWP, Neovius, Schwarz P, and Schwarz D.

The spinodoid topology can be numerically generated by Gaussian random field (GRF) (*37*), i.e.

$$\phi(\boldsymbol{x}) = \sqrt{\frac{2}{N}} \sum_{i=1}^{N} \cos\left(\kappa \boldsymbol{n}_i \cdot \boldsymbol{x} + \mu_i\right) \qquad \text{(S1)}$$

where $\phi(\boldsymbol{x})$ is the phase field, representing the concentration fluctuation of one phase at position $\boldsymbol{x} \in \Omega$ in a domain $\Omega \subset \mathbb{R}^3$. N is the number of waves, κ is the wavenumber, $\boldsymbol{n}_i$ and μ_i are the directions and phase angles of the ith wave vector, respectively, which are independently sampled from uniform probability distributions. Spinodoid anisotropy is determined by orientation distribution functions (ODFs), which constrain the sampling of the wave vector to each of the three orthogonal directions, defined by angular ranges θ_1, θ_2, and θ_3. Isotropic architecture ($\theta_1 = \theta_2 = 0$, $\theta_3 = \pi/2$) arises from uniform ODF sampling in the unit sphere, while lamellar ($\theta_1 = \theta_2 = 0$, $0 < \theta_3 < \pi/2$) and columnar ($0 < \theta_1$, $\theta_2 < \pi/2$, $\theta_3 = 0$) configurations emerge from axis-aligned ODF constraints, enabling programmable anisotropic design via angular parameterization (*60*). As $\phi(\boldsymbol{x})$ evolves from a random distribution, the two phases split to form a bicontinuous field separated by a smooth interface. The spinodoid is obtained from the region bounded by two surfaces with different isovalues.

Text S2. DLP printing parameters and optimization process

As illustrated in Fig. S47a, the complex Al_2O_3 architectures were fabricated using a bottom-up projection DLP printing system (microArch S240, BMF, China) with a 10 µm XY resolution. In this approach, the three-dimensional model is digitally sliced into a sequence of two-dimensional images (Fig. S47c), which serve as exposure masks for the layer-by-layer fabrication process. The slicing images were generated using Materialise Magics. During printing, the UV projector, located above the vertically adjustable platform, projects patterned digital light through the membrane to initiate photopolymerization of the Al_2O_3 resin in the illuminated regions, thereby forming each solidified layer. Compared with commercial photopolymer resins, the ceramic resin exhibits substantially higher viscosity, necessitating several adjustments in the printing protocol. Both descending and ascending speeds of the build platform were reduced to facilitate resin reflow and ensure uniform recoating. After each layer was exposed, a roller was activated to prevent adhesion

between the printed structure and the membrane. In addition, sacrificial layers and protective walls were incorporated into the printing design (Fig. S47b). The sacrificial layer minimizes structural damage when detaching the printed part from the build platform, while the protective wall stabilizes the structure and acts as a container to “scoop” sufficient resin during each downward stroke, ensuring adequate contact between resin and the exposure zone.

By balancing printing efficiency with structural fidelity, we selected a layer thickness of 20 μm for slicing the architectures. The complete set of optimized printing parameters is summarized in Table S7. In this example structure, images 1–20 correspond to the sacrificial layer, with a total thickness of 0.4 mm. Images 21–270 represent the structural layers together with the protective wall, and the target architecture has a height of 5 mm. For the lower 30 structural layers, the exposure time and light intensity were slightly increased to enhance structural integrity, preventing collapse or membrane adhesion at the base region. The higher exposure time and intensity applied to the upper portion of the sacrificial layer were designed to facilitate clean separation of the printed structure from the sacrificial layer during post-processing.

The light intensity, and layer thickness (defined as the platform descent distance minus the ascent distance) are the critical parameters governing the printability and structural fidelity of ceramic architectures. We systematically evaluated and optimized all three parameters. Exposure time and light intensity directly determine the degree of photopolymerization and thus strongly influence the formability of the printed structures. A shorter exposure time or lower light intensity resulted in insufficient curing of each layer at the interface between the build platform and the membrane. Consequently, the printed structures tended to collapse, lose edges, or adhere to the membrane. In contrast, excessively long exposure or high intensity led to over-curing, which caused partial fusion or blockage of internal pores, adhesion between the structure and protective walls, and ultimately impaired catalytic performance. Layer thickness primarily affects printing efficiency and dimensional accuracy. For ceramic resin, a thickness of 20 μm is widely used and considered optimal, as it balances micron-level resolution with reasonable printing time and manageable total layer count during rapid fabrication of complex structures. Through extensive parameter screening and practical iteration, we identified the optimal combination of exposure time, light intensity, and layer thickness, which is presented in Table S7.

Text S3. Multiphase flow modeling with ozone bubbles

In catalytic ozonation, the reaction environment inherently involves a solid–gas–liquid, three-phase system in which ozone bubbles serve as the gaseous reactant dispersed within the aqueous phase. Considering the potential influence of gas–liquid coupling on mass transfer, local velocity fields, and interfacial shear, ozone bubbles were incorporated into the CFD simulations. We performed multiphase flow (bubble flow) simulations using COMSOL Multiphysics 6.2 software. In this model, multiphase flow refers to the simultaneous motion of two or more immiscible phases. One phase acts as the continuous phase (liquid water in this case), while the other (ozone gas) is treated as a dispersed phase.

Since our system involves ozone bubbles dispersed in water—with a gas density several orders of magnitude smaller than that of the liquid phase—the bubble flow model, a subtype of the dispersed multiphase flow model, was selected. This model assumes that bubbles occupy only a small volume fraction and travel at their terminal velocity; therefore, only one set of Navier–Stokes equations is solved for the liquid phase, while bubble motion is described using a slip model (*61*). We estimated the ozone gas flux and bubble size in our catalytic system and found that the flow regime is consistent with small and abundant ozone bubbles and a low Reynolds number ($Re < 10$) (*62*). Therefore, we adopted the bubble flow (laminar flow) module for subsequent analysis.

The computational setup was consistent with the laminar-flow simulations. To minimize boundary effects, a half-height domain was retained above the catalyst framework as a buffer zone. Both liquid (water) and gas (ozone) inlets were defined on the top surface of this region, while the outlet was located at the bottom plane of the structure, which corresponded to the liquid carrying ozone bubbles through the interconnected channels. The normal inflow velocity of the liquid was set to 10^{-3} m/s, and the outlet pressure was set to 0 Pa. The molecular weight of ozone was defined as 47.997×10^{-3} kg/mol. The mass flux of the gas inlet was set to 2.94×10^{-5} $kg\cdot m^{-2}\cdot s^{-1}$, corresponding to the actual gas flow rate in the experiment. The external effective gas density at the outlet was set to zero. To capture the temporal evolution of the gas distribution, we employed a time-dependent solver, enabled low gas concentration option, and used a reference pressure of 1 atm and temperature of 293.15 K. Gravity was applied to the model, and the buoyancy-driven motion of gas bubbles was thereby incorporated through the density contrast between the gas and liquid phases within the slip model.

Fig. S48 illustrates the simulated time-dependent behavior of bubble flow (laminar) in the Square, TPMS-gyroid, and Spinodoid-iso catalyst frameworks (all with a relative density of 30%), and visualizes the evolution of the gas-phase volume fraction along the same cross sections analyzed previously for velocity fields. The simulations were conducted for 20 s, showing that the gas-phase volume fraction gradually reached a steady state. For the Square framework, the gas-phase volume fraction within the vertical channels was significantly higher than that near the edges and corners, indicating a pronounced channeling effect caused by geometric confinement. In contrast, the TPMS-gyroid and Spinodoid-iso frameworks exhibited a more uniform gas-phase distribution and local ozone enrichment (red regions) near the structural surfaces.

Fig. S49 further compares the distributions of effective gas density and gas-phase volume fraction among the three structures. In our single-atom catalytic system, ozone acts as a reactant that decomposes on active sites to generate hydroxyl radicals for pollutant degradation. Therefore, the local ozone concentration near the solid–liquid interface directly reflects the structural contribution to catalytic efficiency. Both TPMS-gyroid and Spinodoid-iso exhibited more homogeneous gas density compared to the Square, with substantially higher gas concentrations along the solid–liquid interfaces—particularly pronounced for the Spinodoid-iso structure. Considering that both shell-based architectures possess a much larger specific surface area than the Square, these results demonstrate that the bioinspired shell-based designs significantly enhance catalytic performance through synergistic solid–liquid–gas interactions.

Fig. S50 compares the fluid velocity distributions within the three catalyst frameworks. After reaching steady state, the liquid-phase velocity in the bubble flow (laminar) (Fig. S50a) and the fluid velocity in the single-phase laminar flow (Fig. S50b) showed no significant variation across the cross sections. Although the sparse ozone bubbles introduce minor local deviations due to buoyancy-induced interactions with the surrounding liquid, these effects are limited to small regions and do not alter the overall magnitude or spatial pattern of the velocity field.

Fig. S51 presents the shear velocity distributions under the same conditions. A comparison between the bubble flow (laminar) (Fig. S51a) and the single-phase laminar flow (Fig. S51b) also shows no significant influence on shear velocity. Both TPMS-gyroid and Spinodoid-iso structures maintained significantly higher shear rates than the square lattice, particularly near the fluid–solid interfaces. These results indicate that under the relatively low gas flux adopted in our study, the presence of the gas phase had no significant influence on key hydrodynamic properties such as fluid velocity and interfacial shear rate.

To isolate the pressure drop from gravitational contributions, the hydrostatic component of the pressure field was removed by adding a correction term of $\rho_m gh$ to the original pressure, where ρ_m is a constant effective mixture density calculated from the volume-averaged phase fractions (98.5% liquid and 1.5% gas). This correction ensures that the comparison among different architectures reflects only hydrodynamic effects rather than static head differences. After introducing gas bubbles while maintaining a velocity inlet boundary condition, moderate changes in the pressure fields were observed among the three architectures. As shown in Fig. S52, relative to the single-phase laminar flow, all the three structures exhibited a slight increase in pressure drop. These subtle variations are attributed to geometry-dependent alterations in local flow pathways and velocity profiles induced by the presence of dispersed bubbles. Gravity induces an upward slip tendency of the lighter gas phase, which is compensated by an adjusted pressure field to maintain the prescribed inlet velocity. Importantly, despite these minor variations in pressure magnitude, the quantitative ordering of pressure drops among the architectures remained unchanged.

Text S4. Details of calculation of formation energy and adsorption energy

The formation energies (E_f) of Mn-N_xC_y (x+y = 4, x = 1-4) were calculated through the following expression (Eq. S2) (*63*)

$$E_f = E_{Mn\text{-}NxCy} + y\mu_c - (E_{graphene} + x\mu_N + E_{Mn}) \qquad \text{(S2)}$$

Here, $E_{Mn\text{-}NxCy}$ is the energy of the optimized carbon skeleton with Mn-N_xC_y defects. $E_{graphene}$ is the energy of the optimized pristine carbon skeleton, μ_c is the chemical potential of carbon defined as the total energy of graphene per C atom. μ_N is the chemical potential of nitrogen defined as half of the total energy of an N_2 molecule and E_{Mn} is the energy of an Mn ion. The adsorption energies (E_{ads}) of O_3/OH^- on Mn-N_4 site were calculated through the following expression (Eq. S3)

$$E_{ads} = E_{total} - E_{carbon\ skeleton} - E_{mole} \qquad \text{(S3)}$$

Here, $E_{carbon\ skeleton}$ is the energy of the optimized carbon skeleton with Mn-N_4 defects, E_{mole} is the energy of the O_3/OH^-, and E_{total} is the energy of complex of O_3/OH^- and carbon skeleton with Mn-N_4 defects.

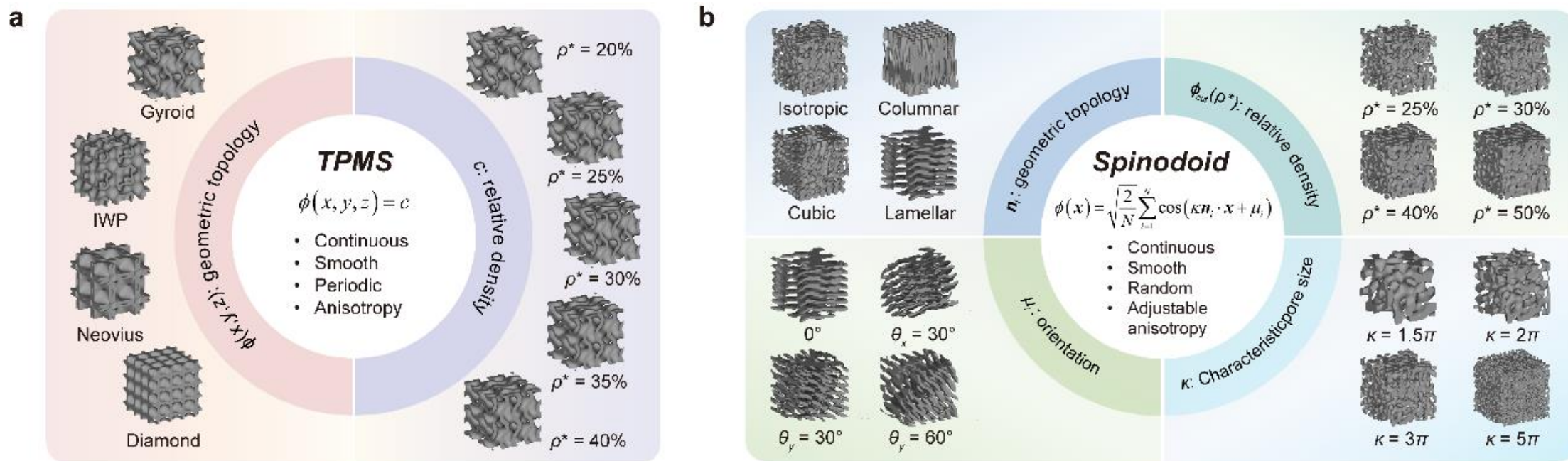

Fig. S1. Catalyst design strategy integrating 3D smooth, continuous, and shell-based architectures. a, Schematic illustration of TPMS generation by tuning the level-set equation. **b,** Schematic illustration of spinodoid generation by tuning the parameters of Gaussian random field (GRF).

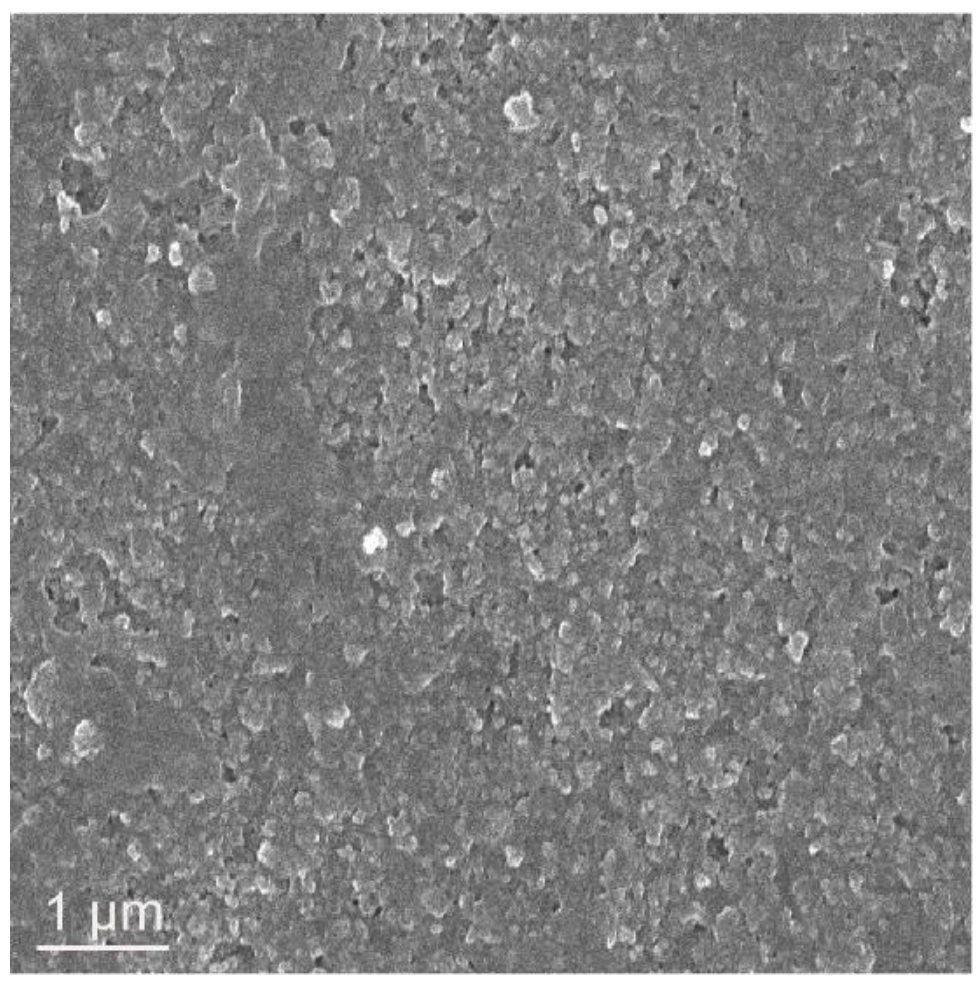

Fig. S2. SEM image of Mn-ISAs@AAF catalyst.

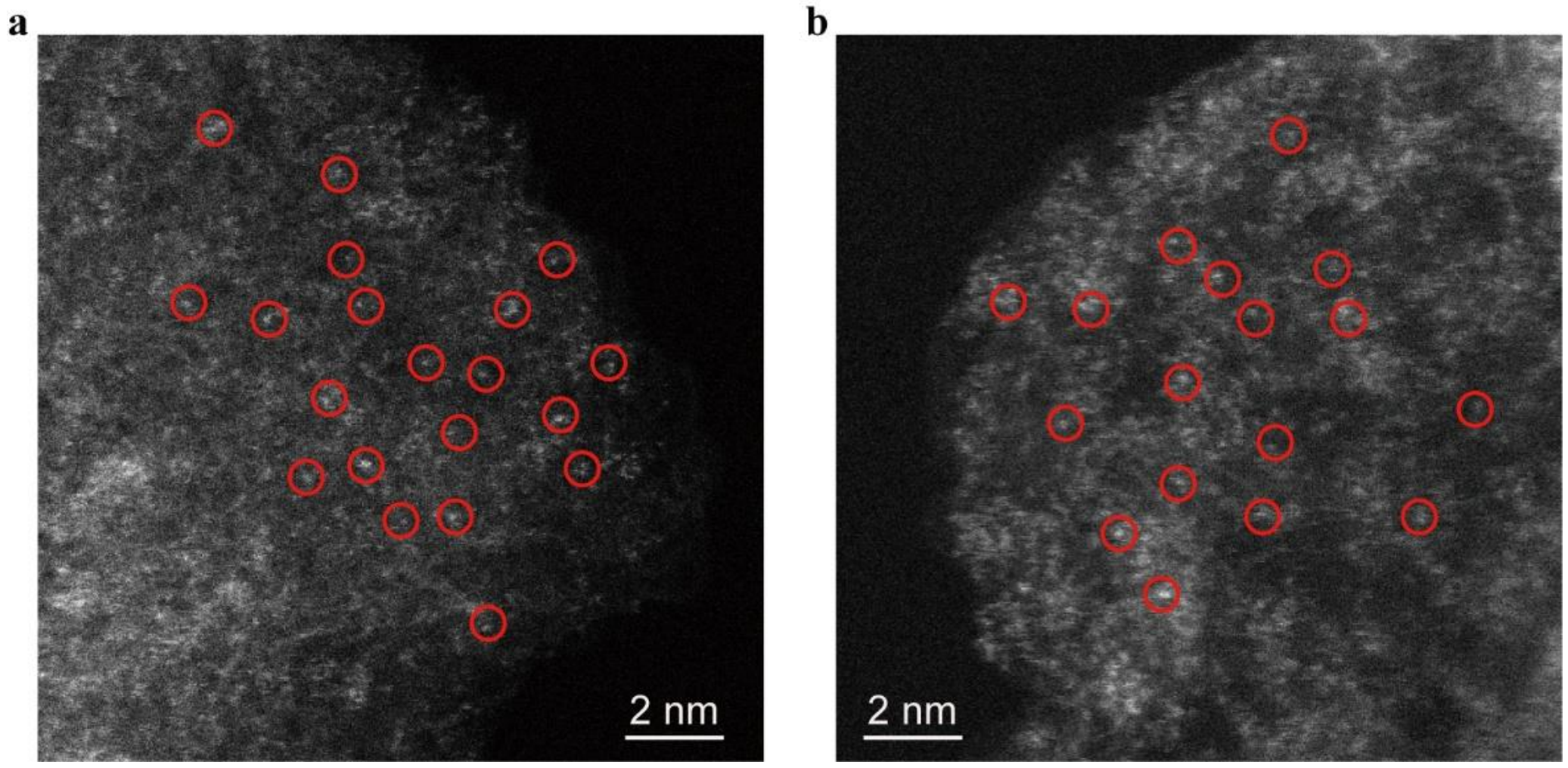

Fig. S3. HAADF-STEM images of Mn-ISAs. The images were randomly sampled at different locations of Mn-ISAs@AAF catalyst.

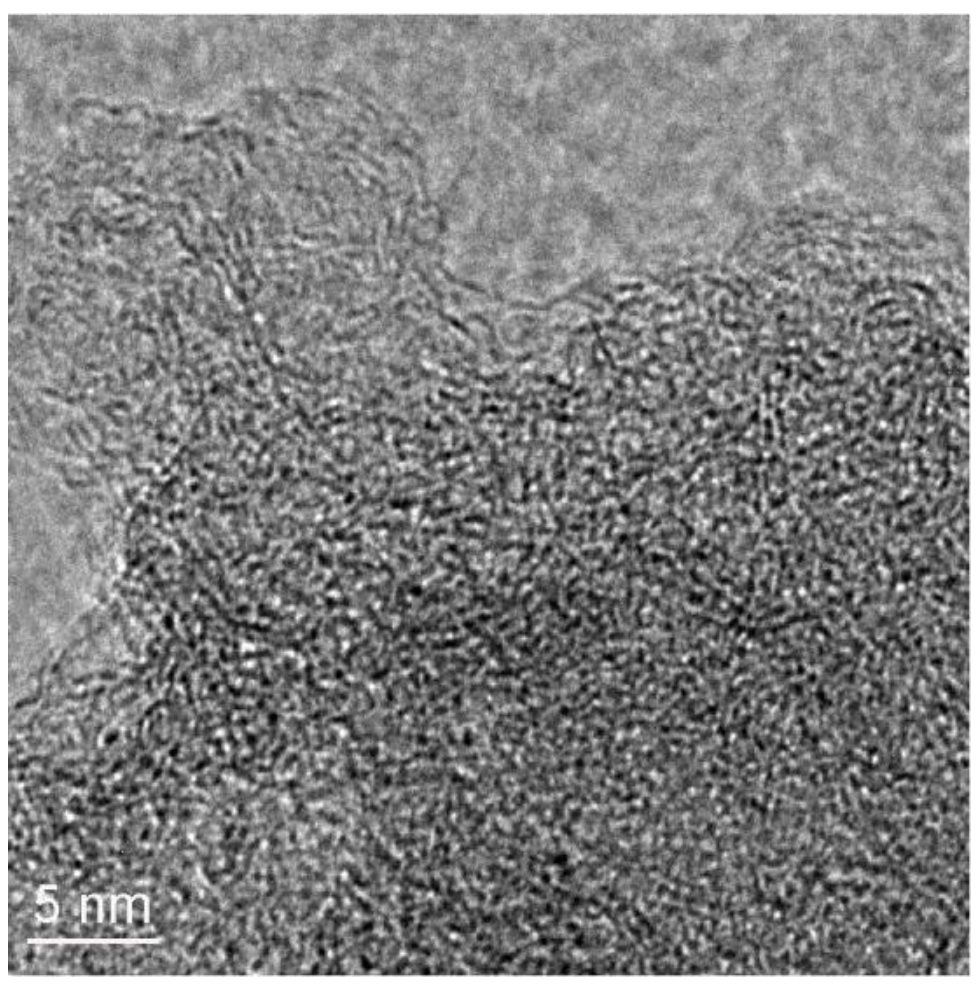

Fig. S4. HRTEM image of Mn-ISAs. No other crystal phases associated with the metallic Mn species were observed.

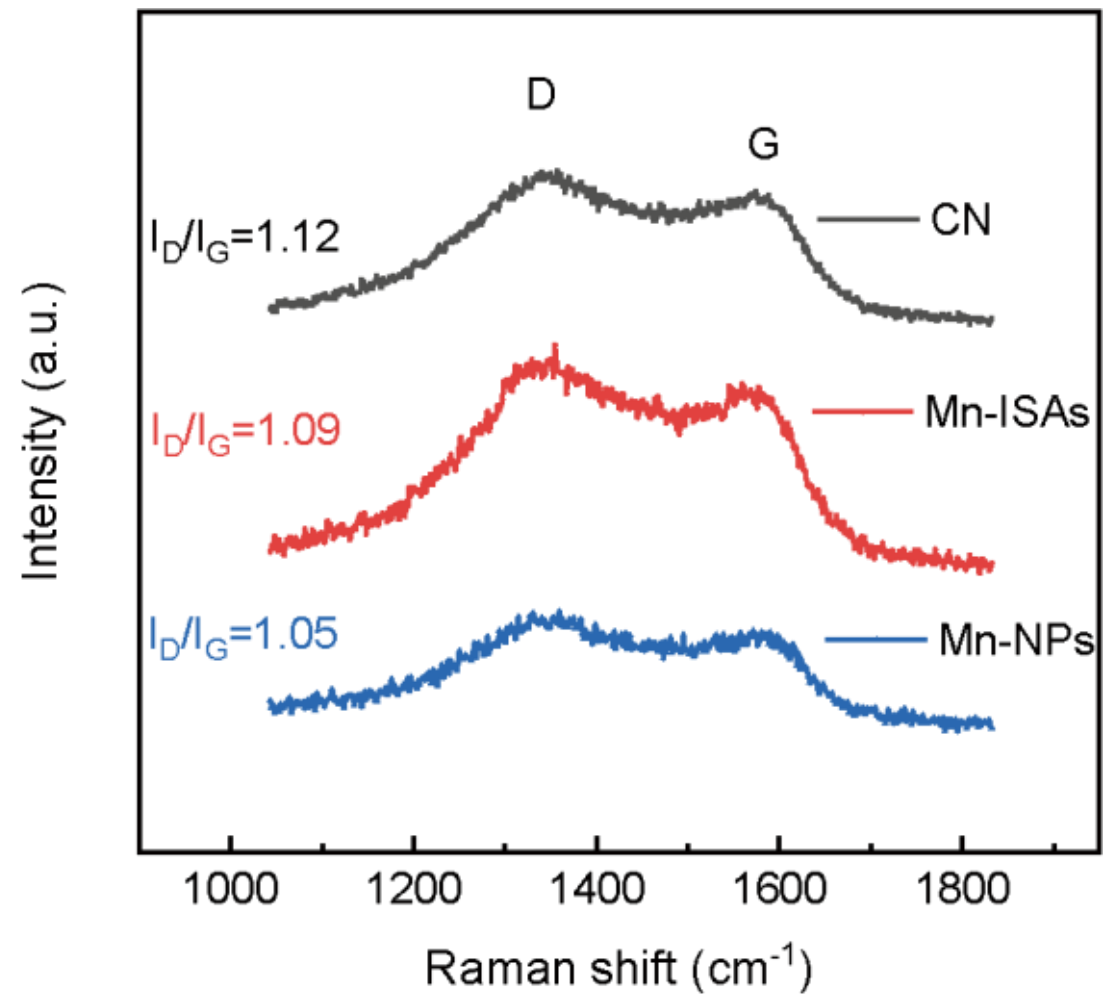

Fig. S5. Raman spectra of CN, Mn-ISAs and Mn-NPs. The enhanced graphitization is attributed to the metal-catalyzed graphitization effect of Mn nanoparticles, which promotes carbon atom diffusion, lattice rearrangement, and defect healing during high-temperature pyrolysis.

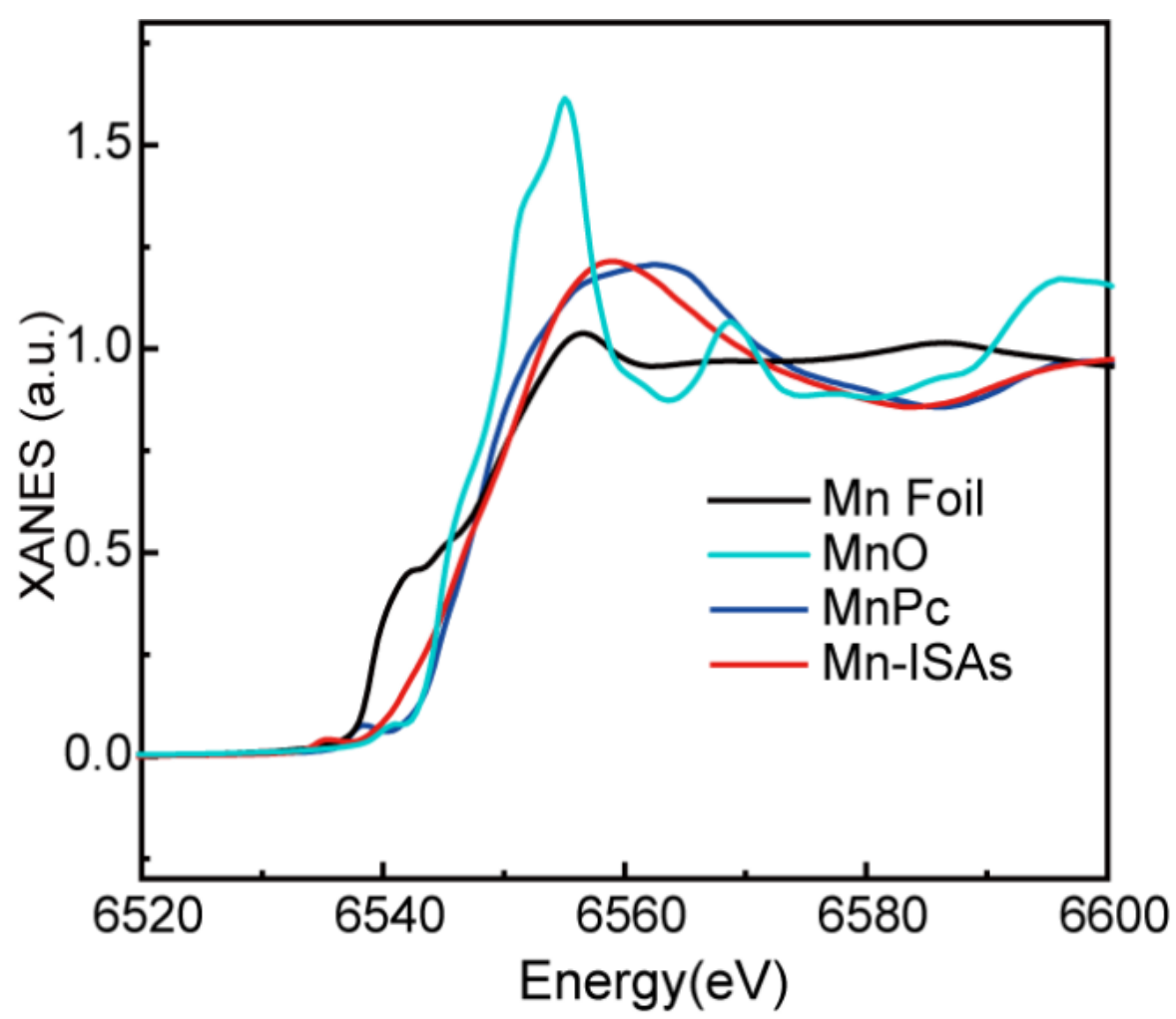

Fig. S6. X-ray absorption near-edge structure (XANES) analysis of Mn sites. The absorption edge of Mn-ISAs fell between those of Mn foil and MnO, indicating a positively charged oxidation state of Mn.

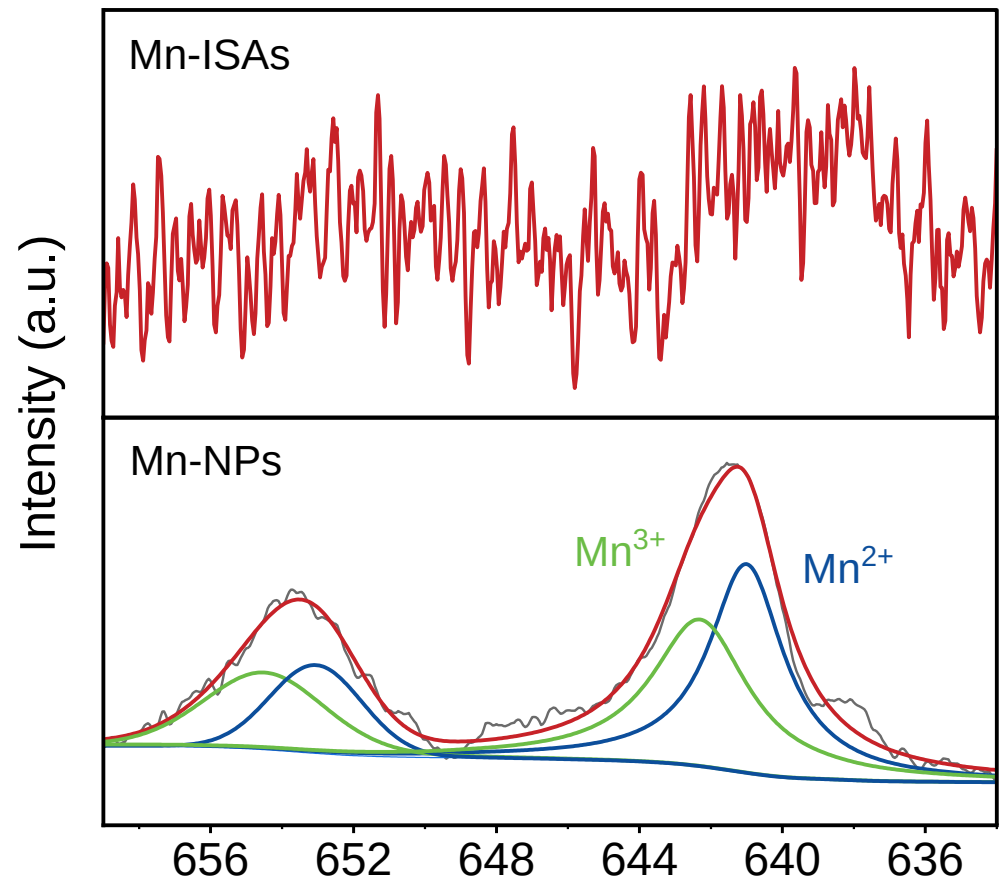

Fig. S7. High-resolution Mn 2p XPS spectra of Mn-ISAs and Mn-NPs. It shows a very weak and broadened signal, which is expected given the ultralow Mn loading (0.08 wt%) and the atomic dispersion of Mn. Importantly, the spectrum does not display the characteristic multiplet splitting or satellite features typically associated with Mn^{2+}/Mn^{3+} oxides, indicating the absence of MnO_x clusters or nanoparticles formation. In contrast, the Mn-NPs sample exhibits well-defined Mn^{2+}/Mn^{3+} peaks together with strong shake-up satellites, which are the typical fingerprints of MnO_x nanoparticles. The clear difference between two Mn 2p profiles confirms that Mn in Mn-ISAs exists in a highly dispersed coordination environment, distinct from the oxide-like states observed in Mn-NPs.

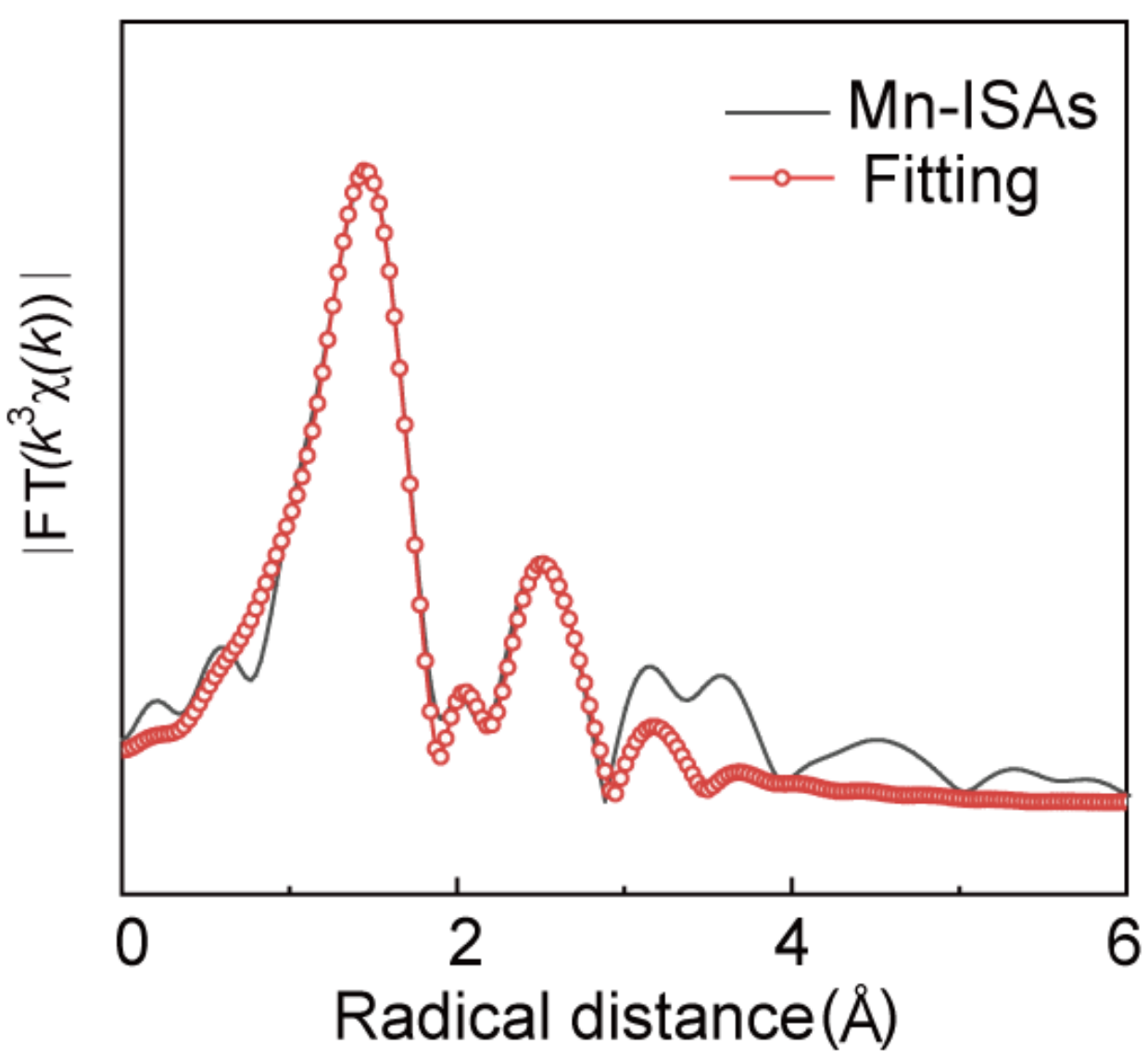

Fig. S8. Corresponding Mn K-edge EXAFS fitting curve. The fitting curve had a very high degree of fit with the original test curve.

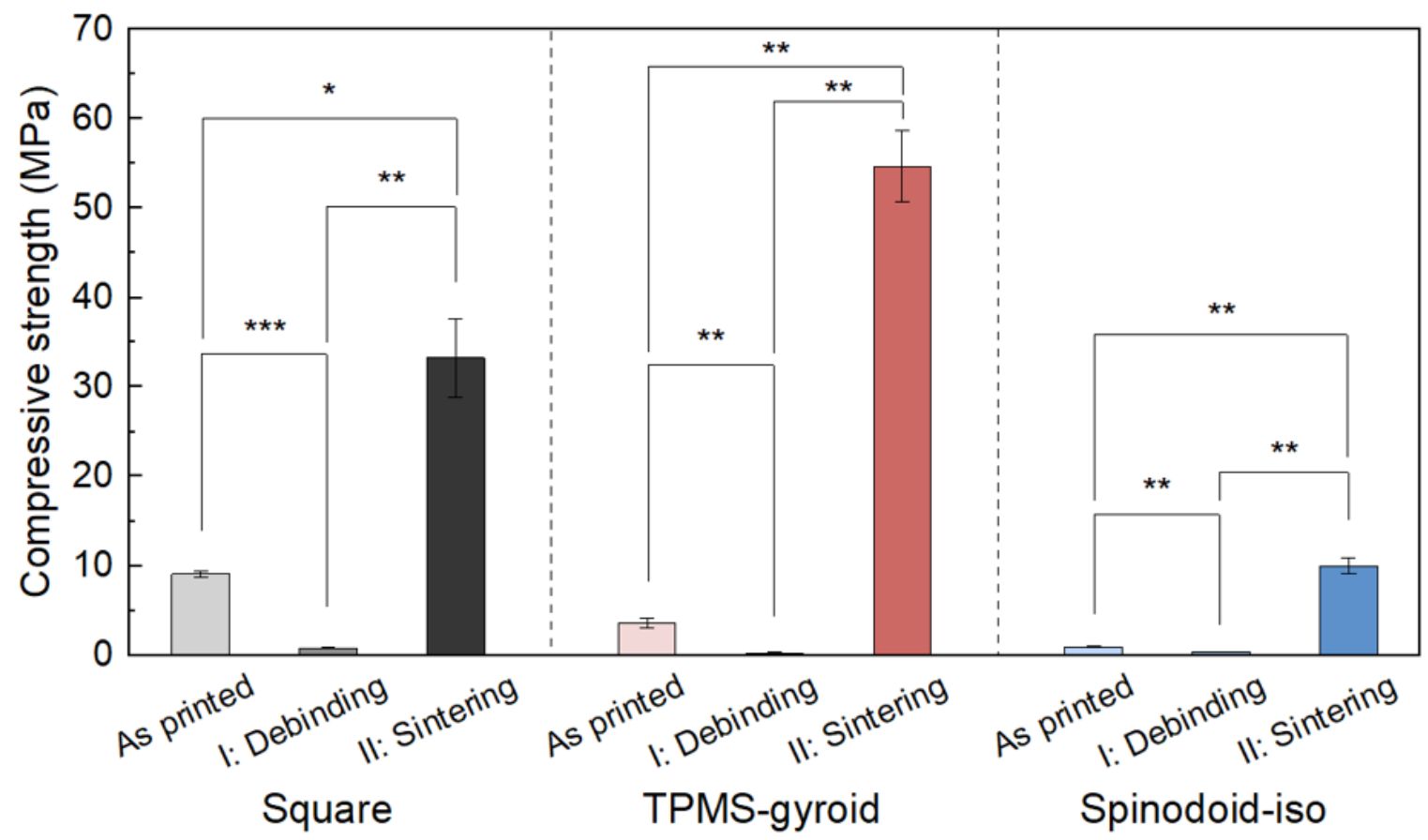

Fig. S9. Compressive strength of the Square, TPMS-gyroid, and Spinodoid-iso catalyst frameworks.

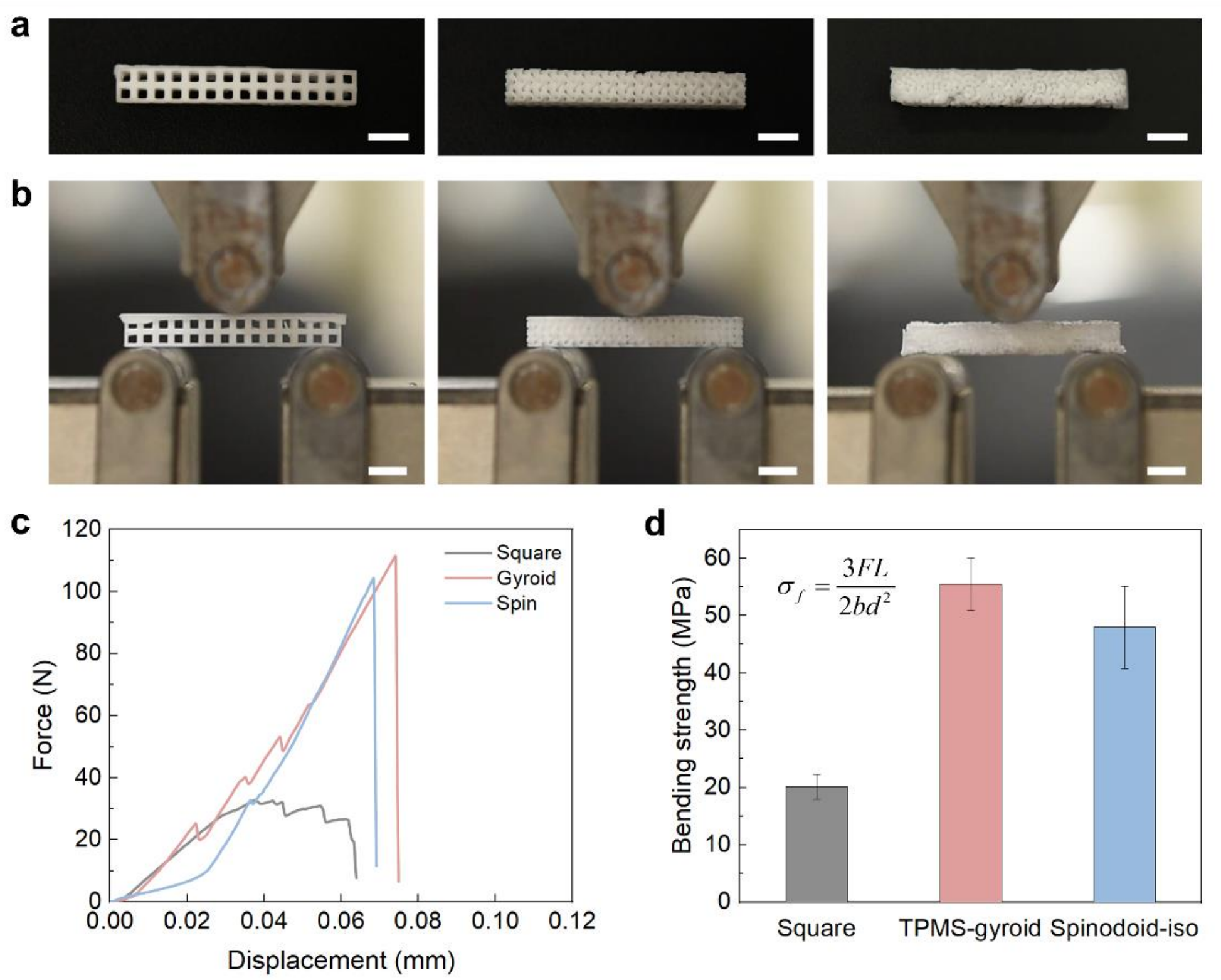

Fig. S10. Three-point bending tests and bending strength of the sintered specimens. a, Sintered Square, TPMS-gyroid, and Spinodoid-iso bending specimens. Scale bar, 5 mm. **b,** Three-point bending test loading conditions. Scale bar, 5 mm. **c,** Representative force–displacement curves of the bending specimens. **d,** Comparison of bending strengths for Square, TPMS-gyroid, and Spinodoid-iso frameworks.

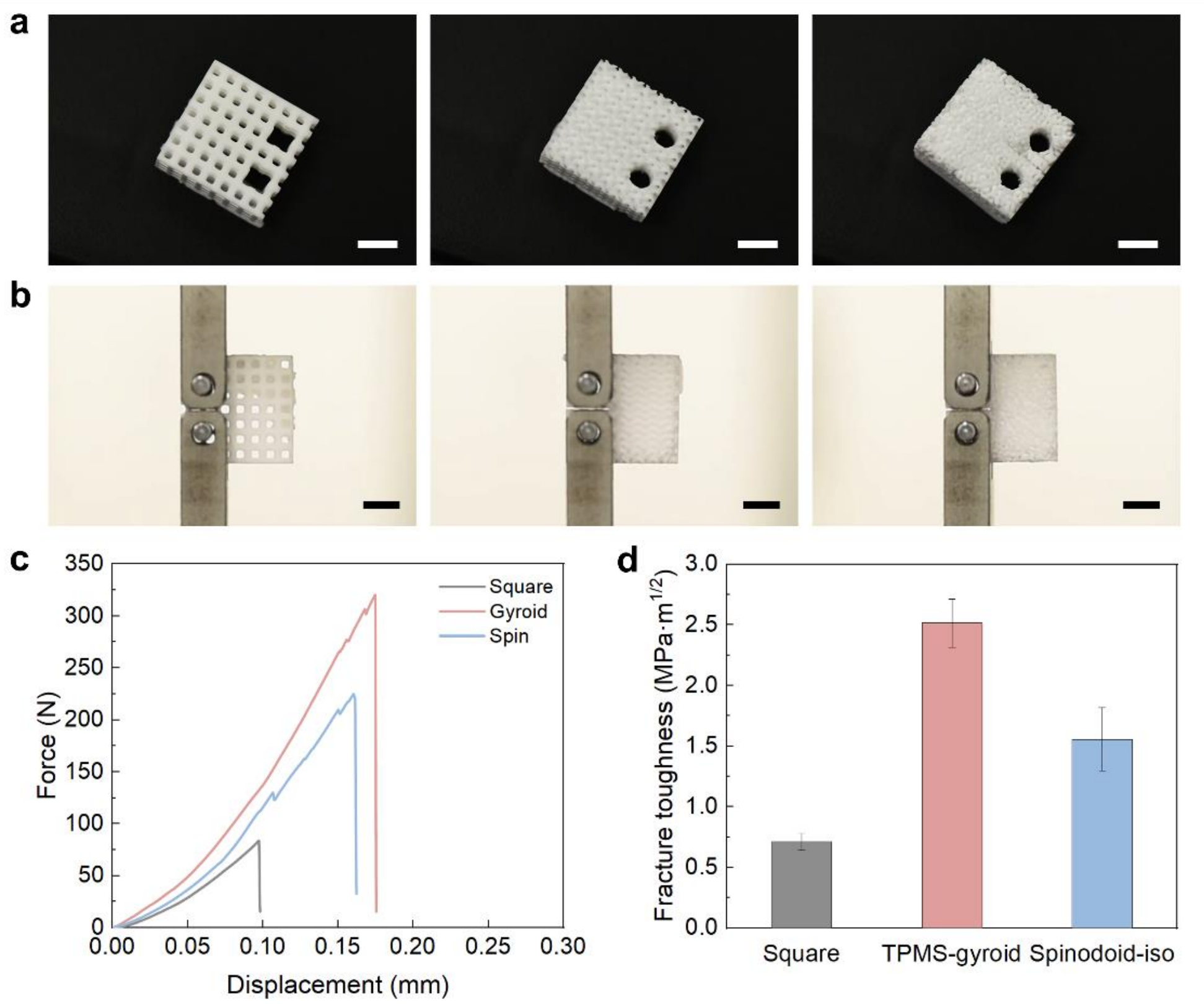

Fig. S11. Fracture tests and fracture toughness of the sintered specimens. a, Sintered Square, TPMS-gyroid, and Spinodoid-iso CT specimens. Scale bar, 5 mm. **b,** Fracture test loading conditions. Scale bar, 5 mm. **c,** Representative force–displacement curves of the CT specimens. **d,** Comparison of fracture toughness for Square, TPMS-gyroid, and Spinodoid-iso frameworks.

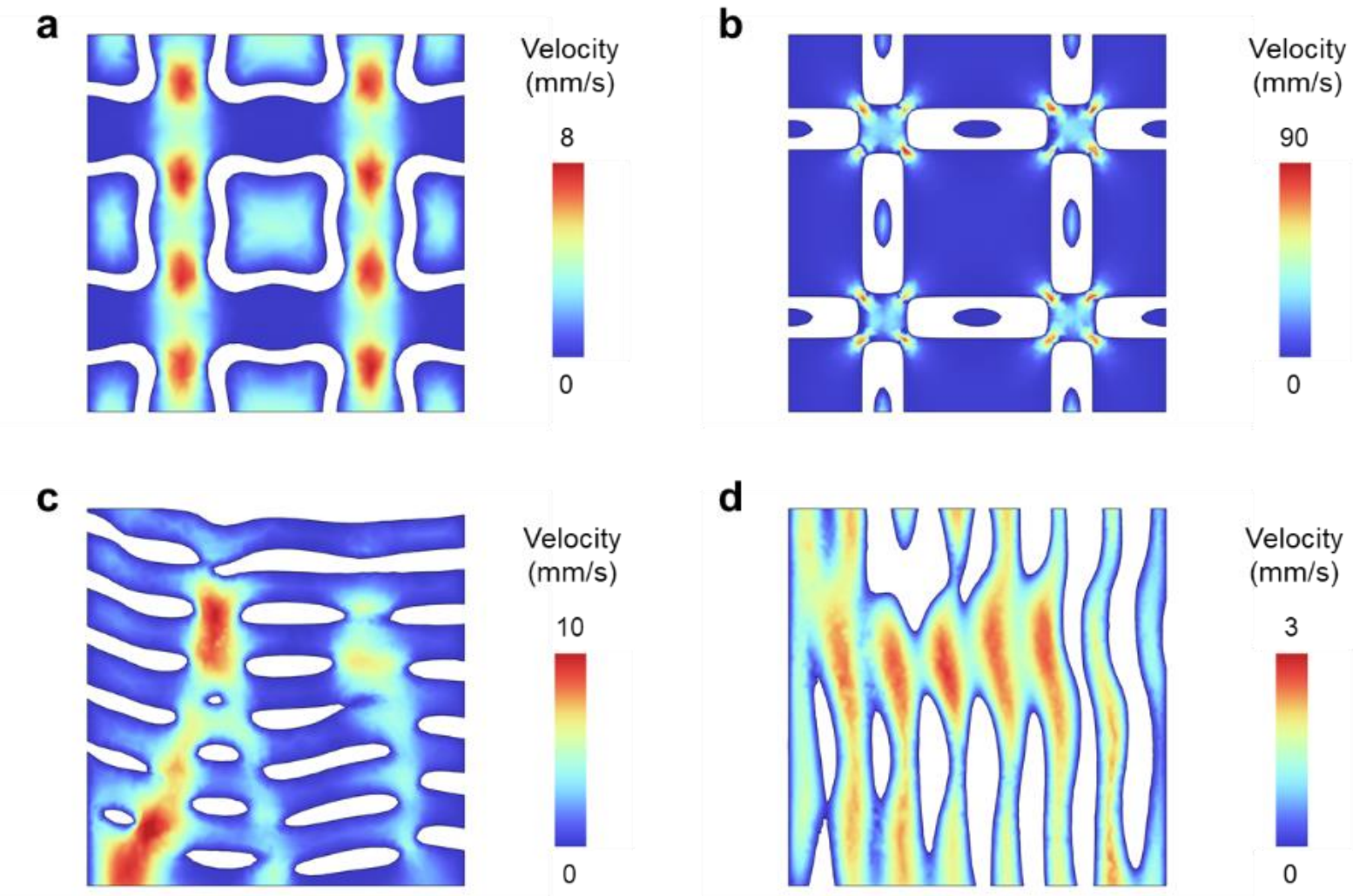

Fig. S12. Fluid velocity distribution in vertical cross-sections of four 30% relative density catalyst frameworks. a, TPMS-IWP. **b,** TPMS-Neovius. **c,** Spin-lam. **d,** Spin-col.

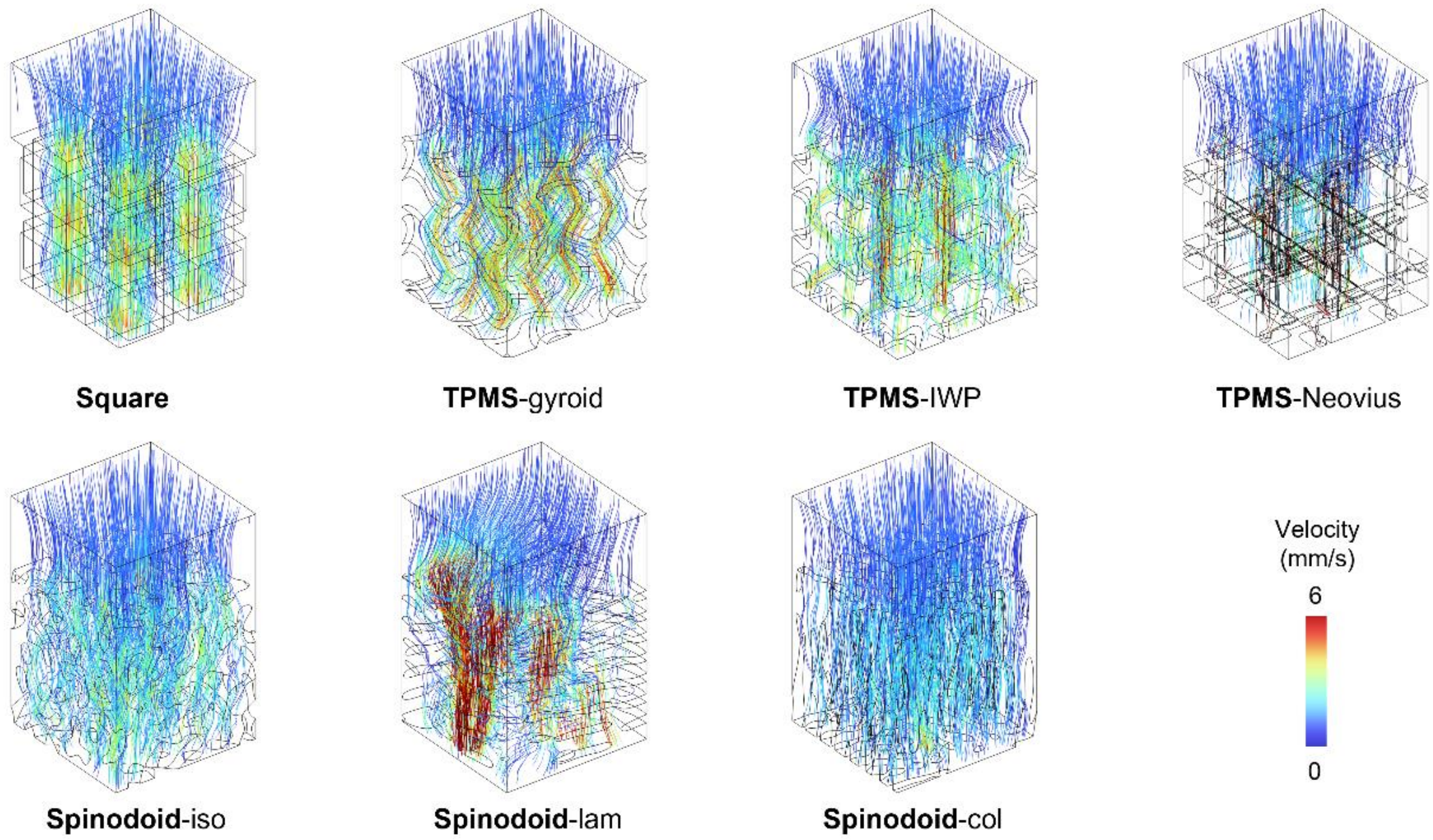

Fig. S13. Simulated particle trajectory distributions of the Square, TPMS-gyroid, TPMS-IWP, TPMS-Neovius, Spinodoid-iso, Spinodoid-lam, and Spinodoid-col catalysts with a relative density of 30%. The trajectory distributions closely mirror the flow streamlines and effectively capture the channelization characteristics of each structure. For instance, the Square, TPMS-Neovius, and Spinodoid-lam frameworks exhibit pronounced channeling behavior, with certain regions showing very high velocities while other zones form dead regions with limited flow. In contrast, the Spinodoid-iso framework shows a more spatially dispersed trajectory pattern without clear channeling.

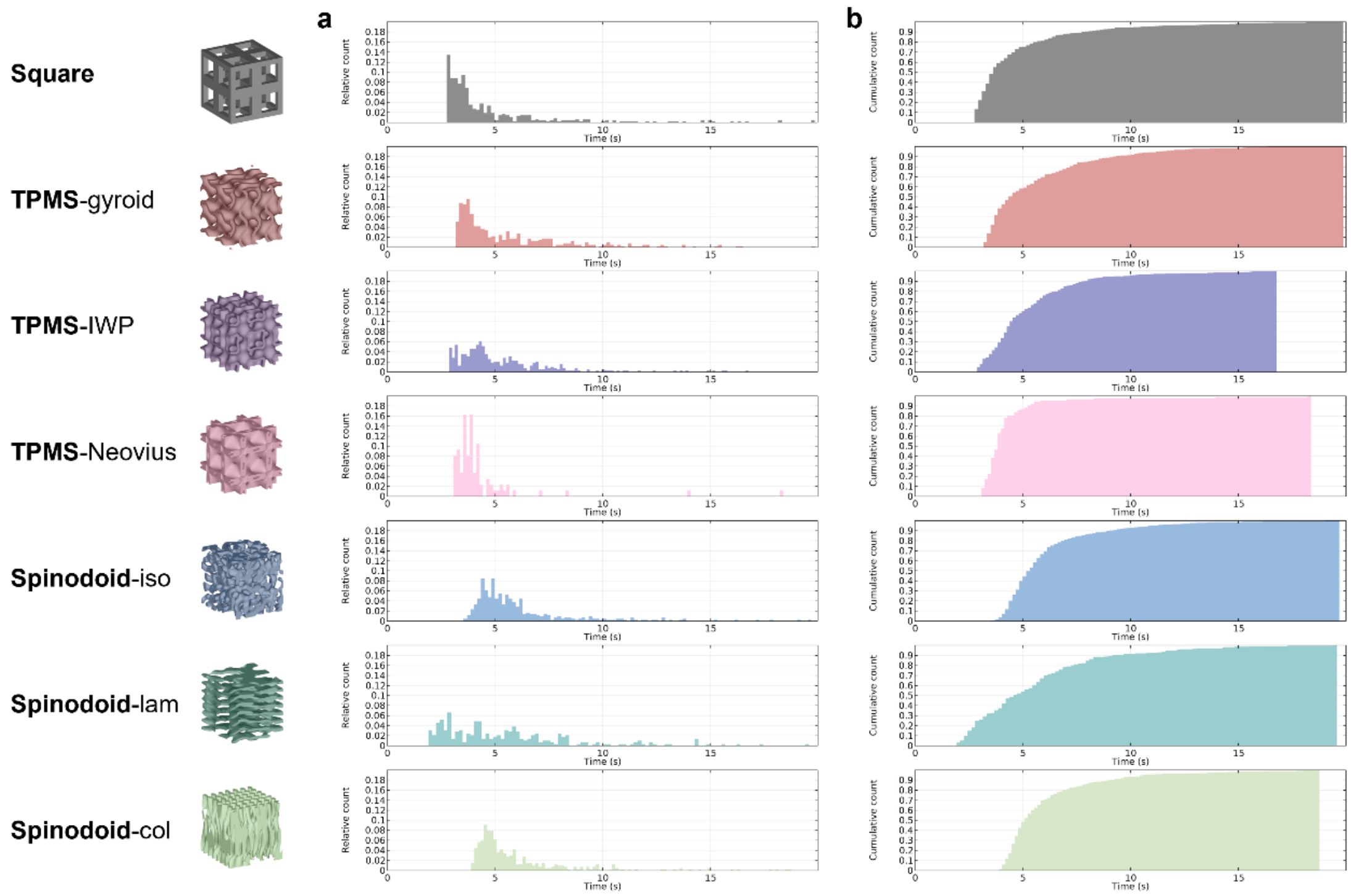

Fig. S14. Simulated probability density distributions of particle residence time across different catalyst frameworks. a, Residence time distribution (RTD). **b,** Cumulative distribution. The Spinodoid-iso framework demonstrates a longer average residence time and a broader distribution, while the Square and TPMS-Neovius structures show shorter and more narrowly distributed residence times, indicating stronger channelization effects.

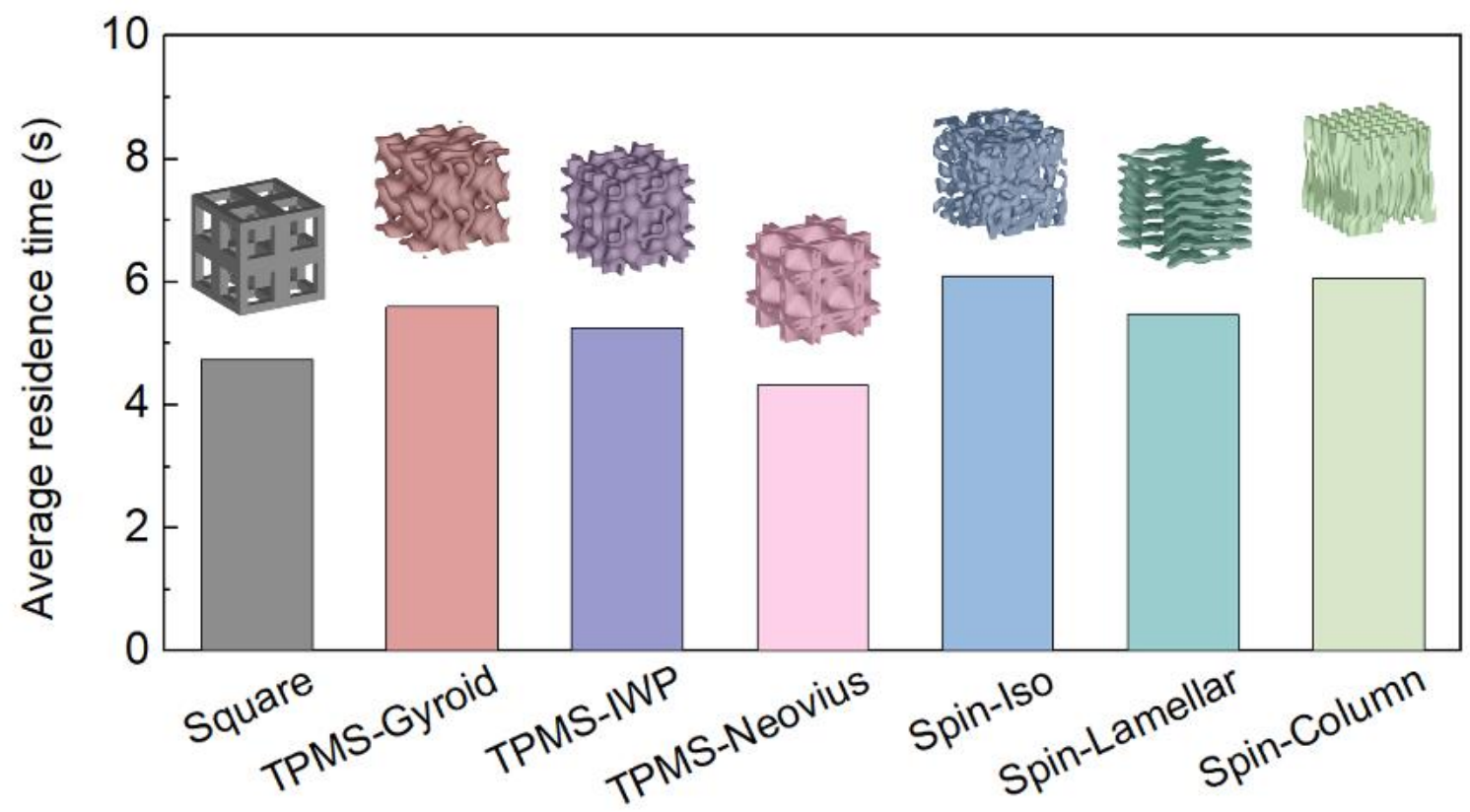

Fig. S15. Comparison of the averaged residence time of different frameworks.

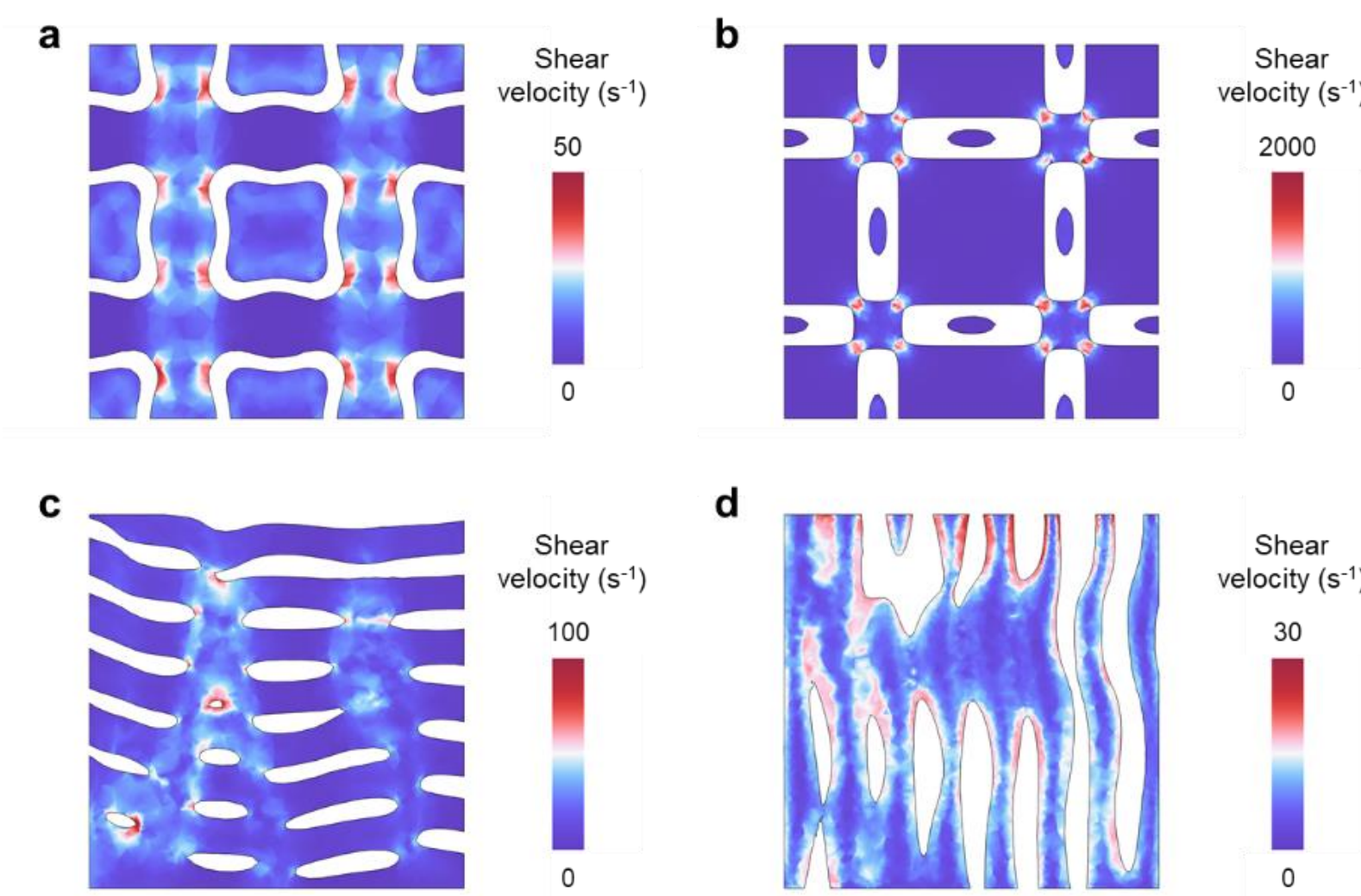

Fig. S16. Fluid shear velocity distribution in 30% relative density catalyst frameworks. a, TPMS-IWP. **b,** TPMS-Neovius. **c,** Spin-lam. **d,** Spin-col.

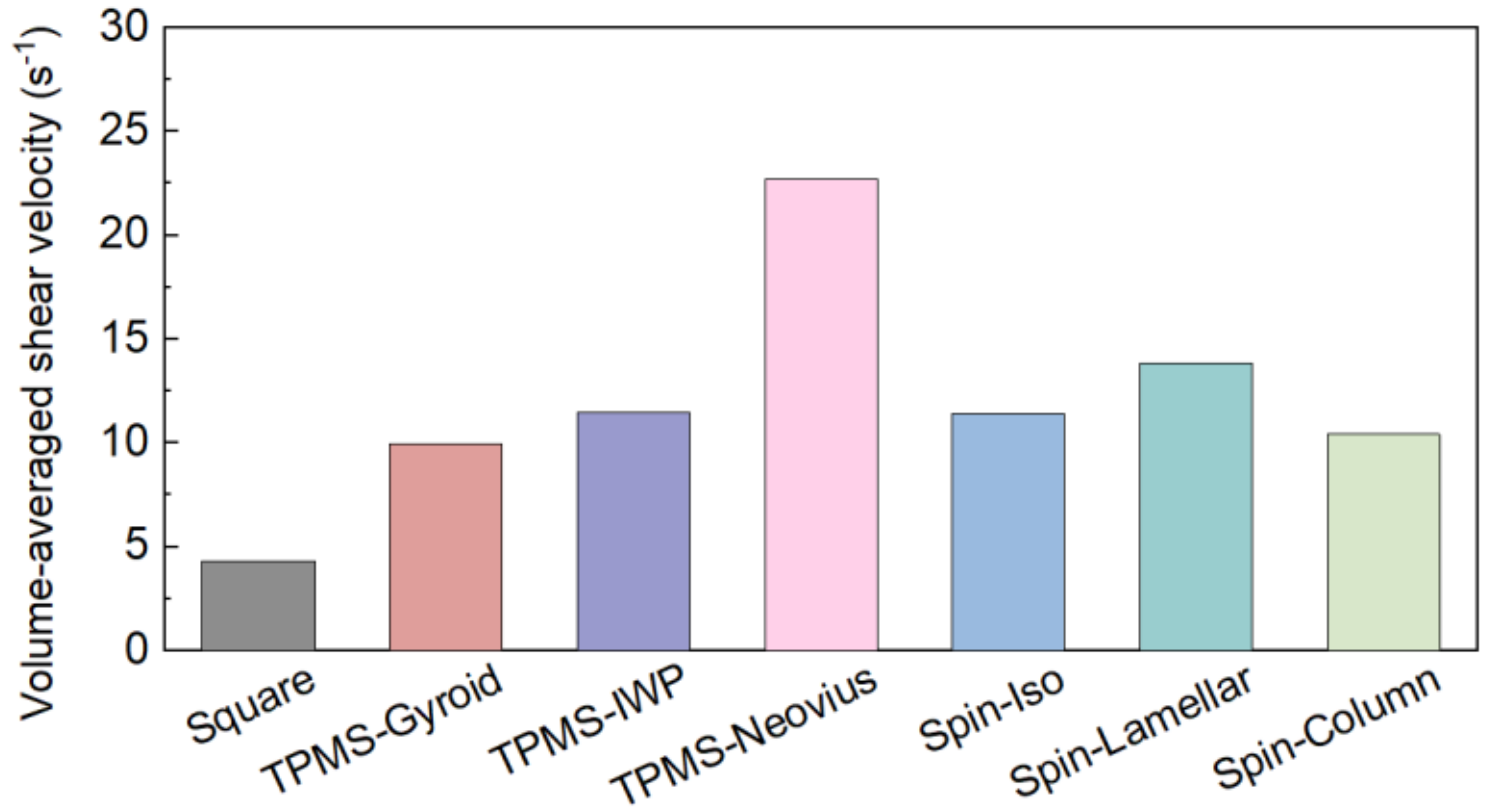

Fig. S17. Comparison of the volume-averaged shear velocity of different frameworks.

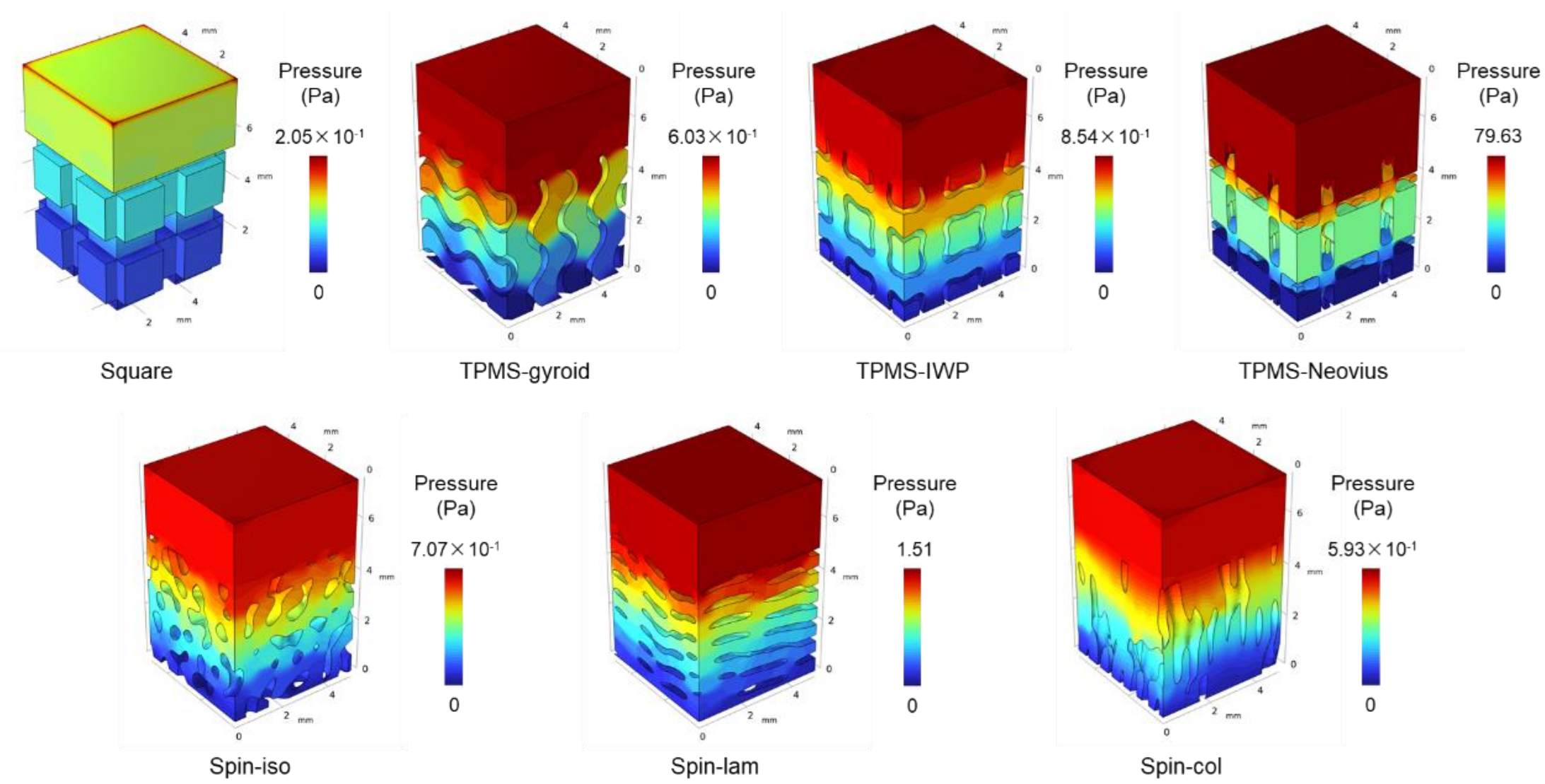

Fig. S18. The pressure distribution of Square lattice, TPMS and spinodoid frameworks with a relative density of 30%.

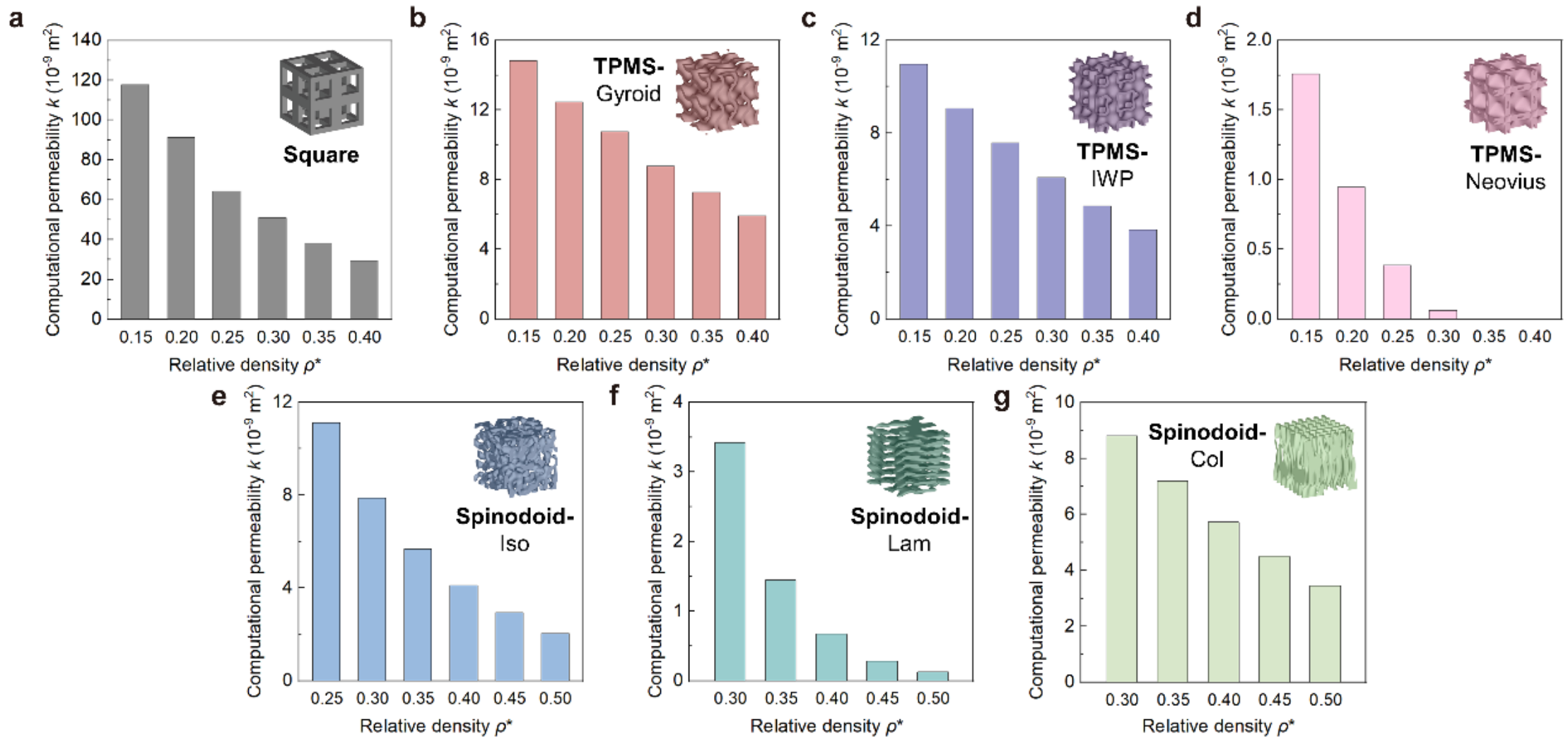

Fig. S19. Computational permeability of TPMS and spinodoid frameworks with different relative density. a, Square lattice. **b,** TPMS-gyroid. **c,** TPMS-IWP. **d,** TPMS-Neovius. **e,** Spinodoid-iso. **f,** Spinodoid-lam. **g,** Spinodoid-col.

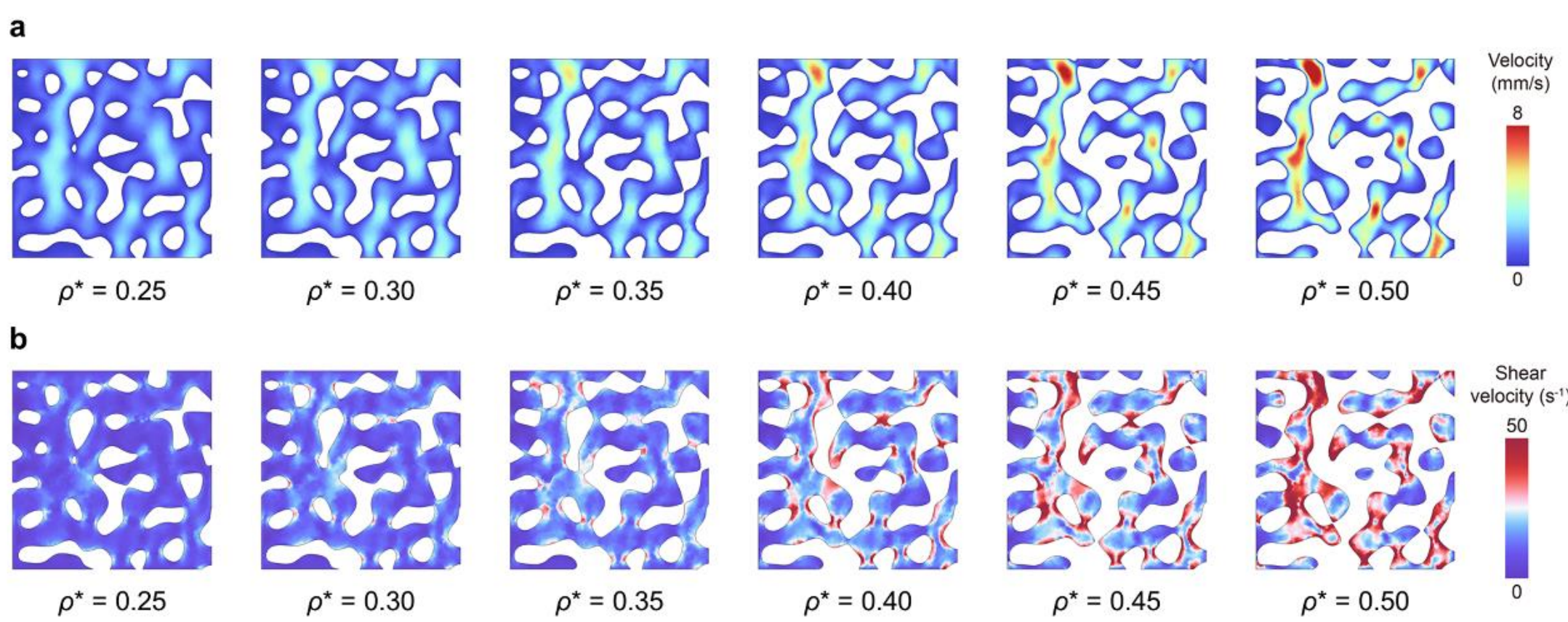

Fig. S20. Fluid velocity and shear velocity distributions in Spin-iso frameworks. a, Fluid velocity and **b,** Fluid shear velocity distribution in the vertical cross-section of Spin-iso frameworks with different relative density.

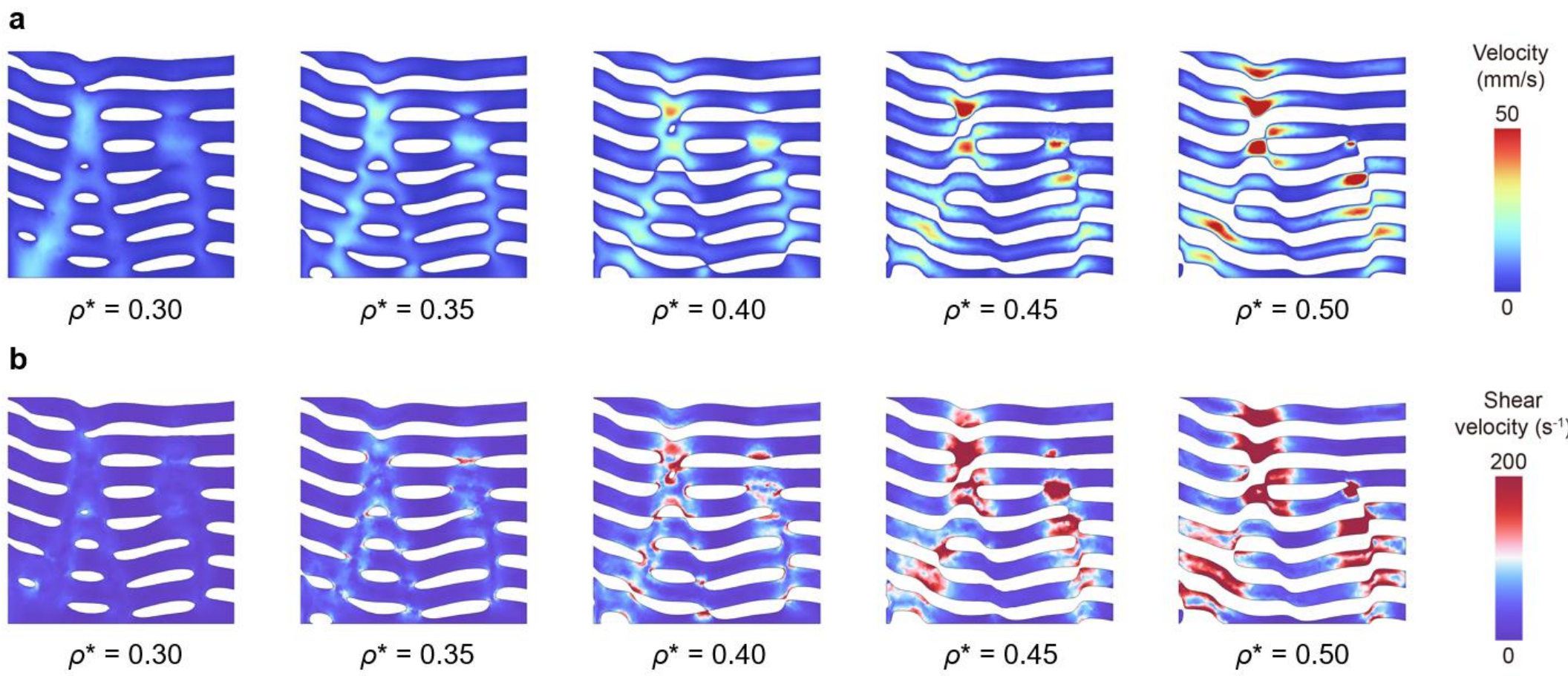

Fig. S21. Fluid velocity and shear velocity distributions in Spin-lam frameworks. a, Fluid velocity and **b,** Fluid shear velocity distribution in the vertical cross-section of Spin-lam frameworks with different relative density.

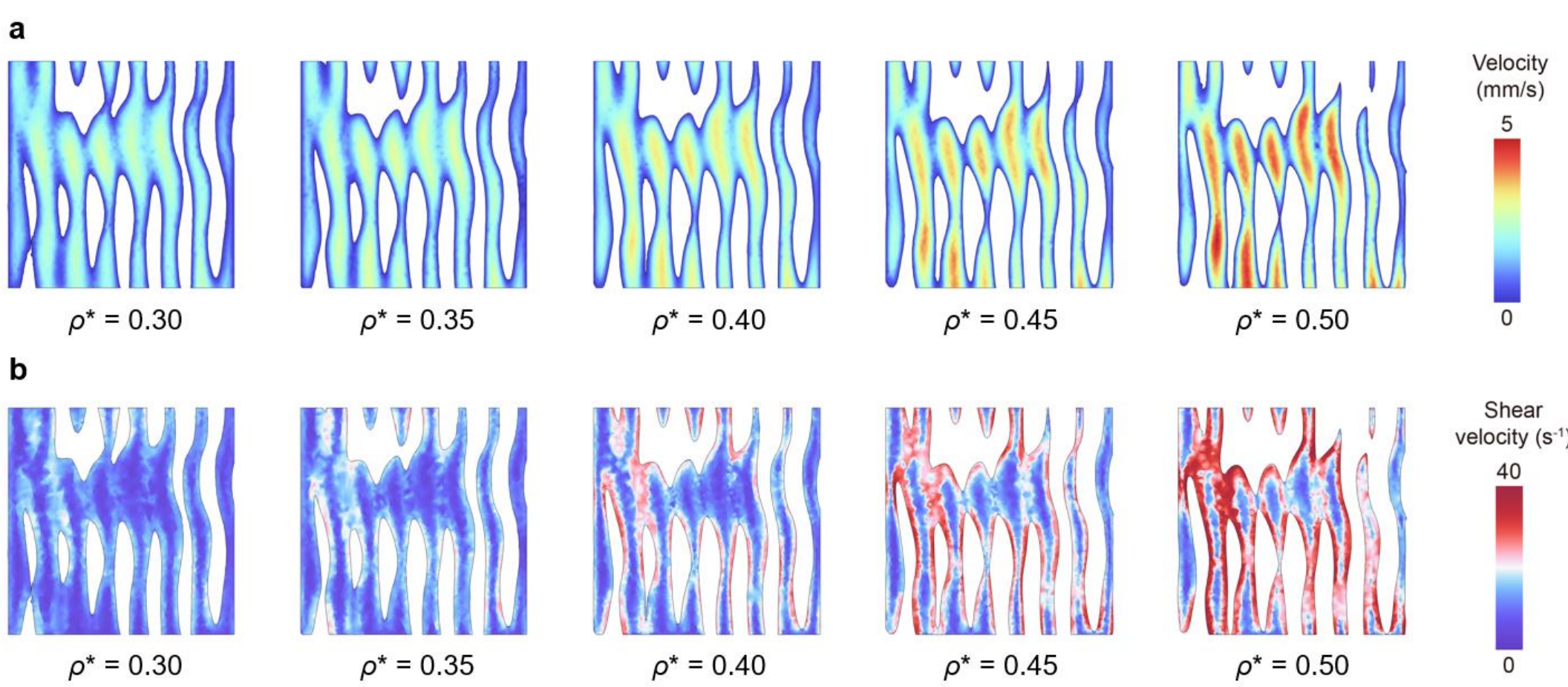

Fig. S22. Fluid velocity and shear velocity distributions in Spin-col frameworks. a, Fluid velocity and **b,** Fluid shear velocity distribution in the vertical cross-section of Spin-col frameworks with different relative density.

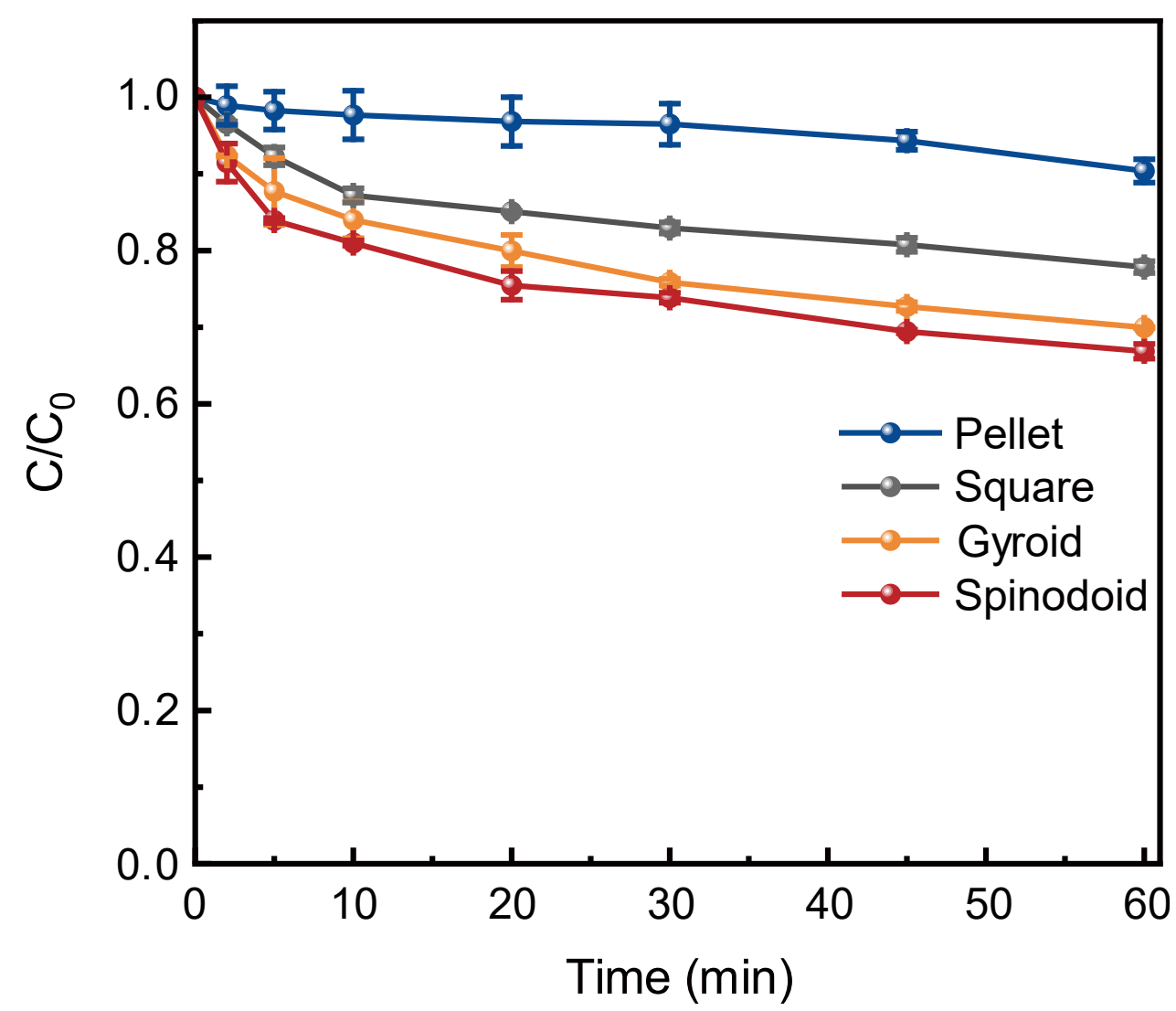

Fig. S23. OA adsorption using different catalysts. Reaction conditions: $[OA]_0$ = 200 mg L^{-1}, catalyst = 4 g, O_3 concentration = 5 mg L^{-1}, O_3 flow rate = 100 mL min^{-1}.

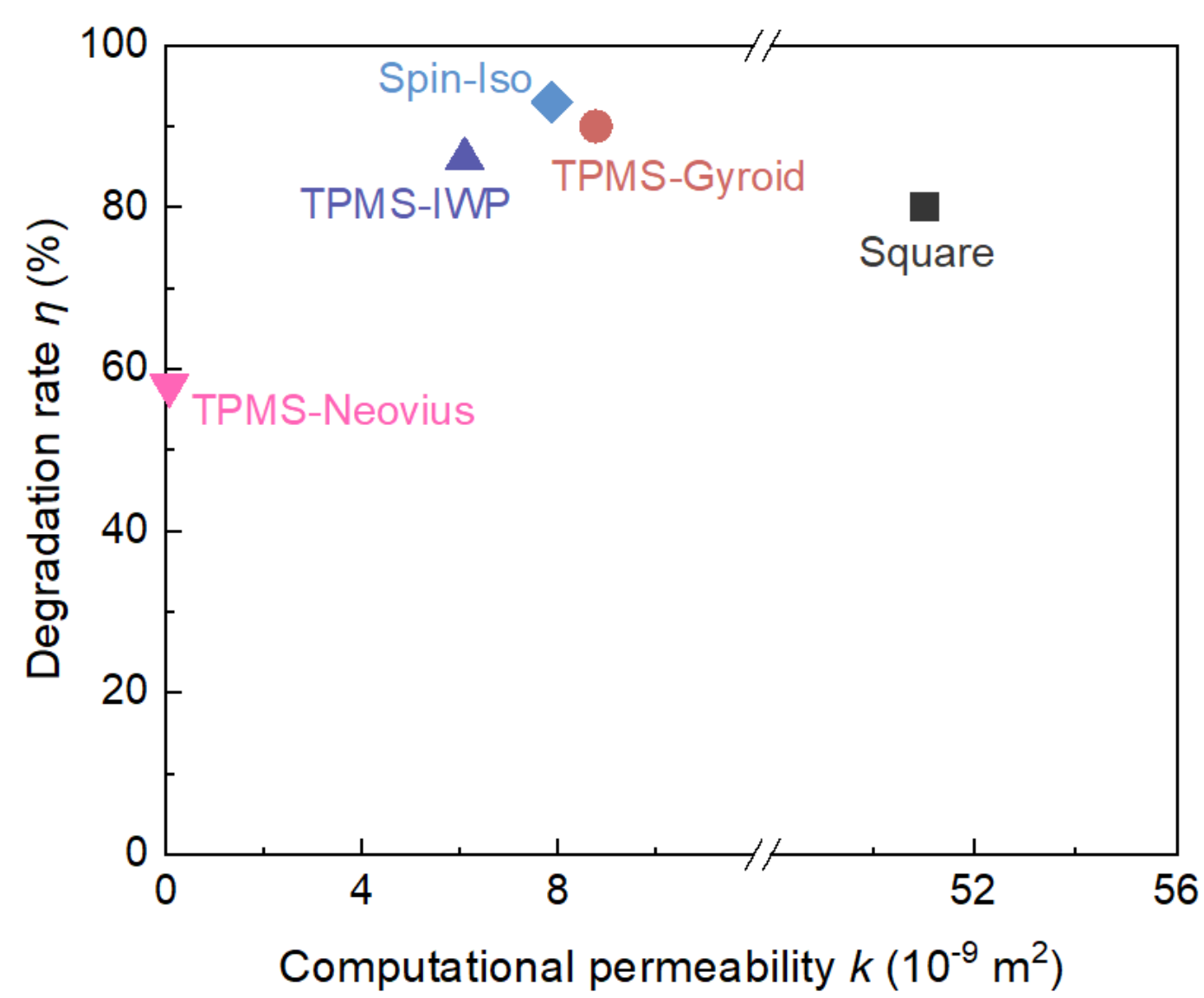

Fig. S24. Correlation between computational permeability and degradation efficiency for different architected catalyst frameworks. It shows the relationship between permeability (k) and degradation efficiency (η) for different catalyst frameworks, including Square, TPMS-Gyroid, TPMS-IWP, TPMS-Neovius, and Spinodoid-iso. The results indicate that the catalytic performance does not increase monotonically with increasing of permeability. Instead, an optimal balance between permeability and accessible surface area leads to enhanced catalytic efficiency, as seen in the TPMS-Gyroid and Spinodoid-iso architectures.

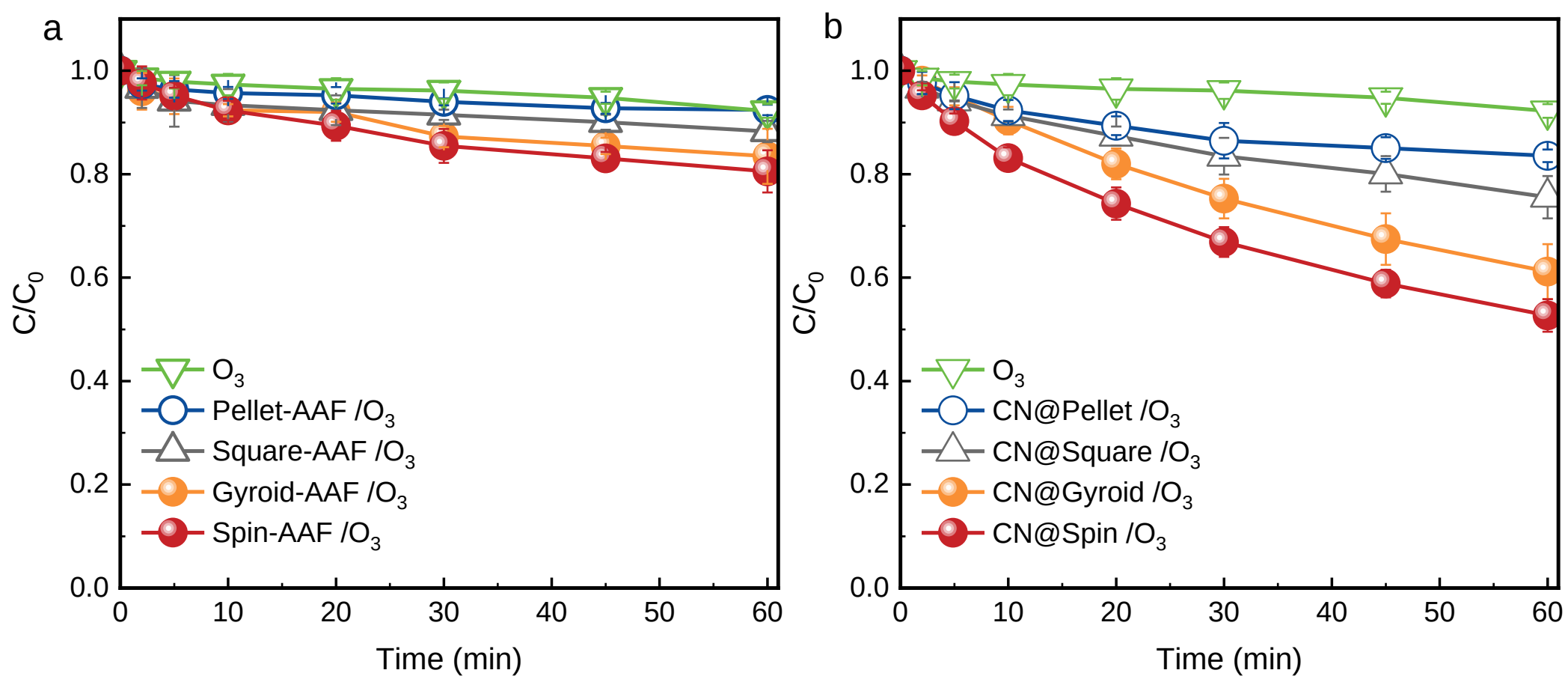

Fig. S25. Ozonation performance and flow characteristics of unmodified and carbon-coated AAF. a, Bare architected alumina frameworks (AAF). **b,** Carbon-coated AAF (CN@AAF) without Mn loading.

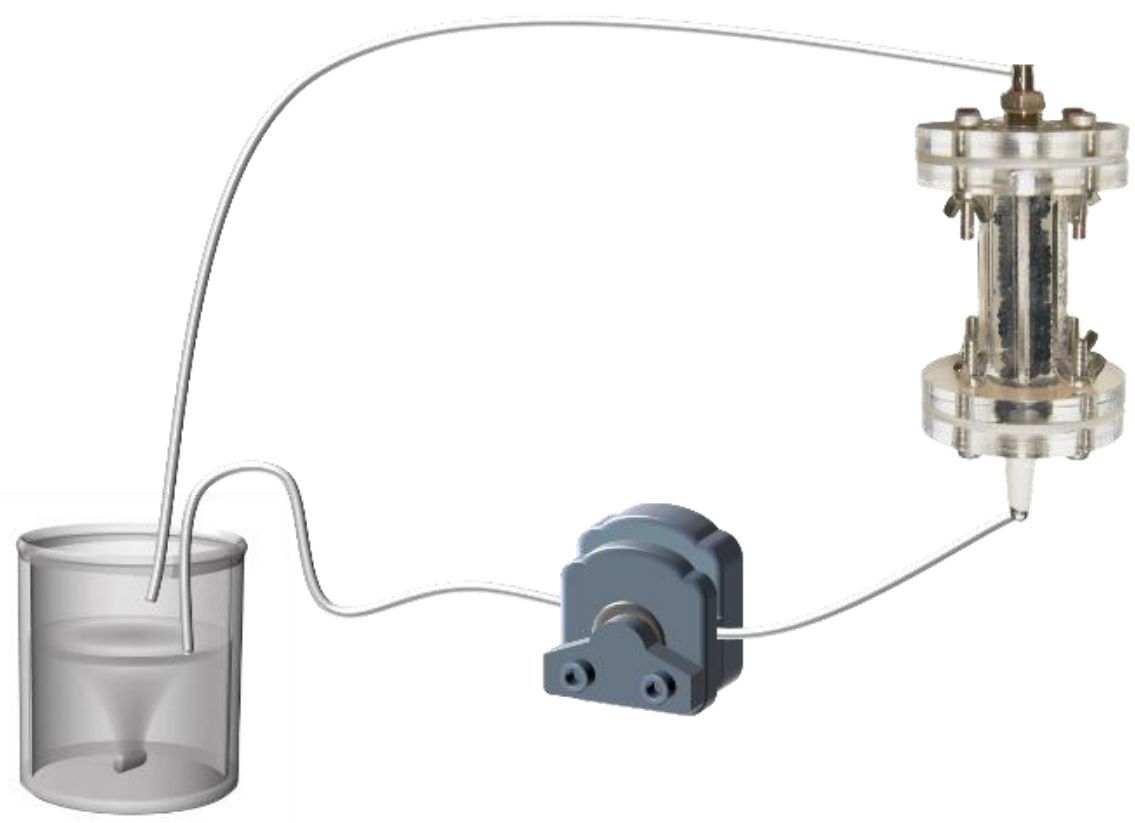

Fig. S26. Schematic diagram of wastewater treatment device. Catalysts were filled in the self-designed reactor with same mass or same volume.

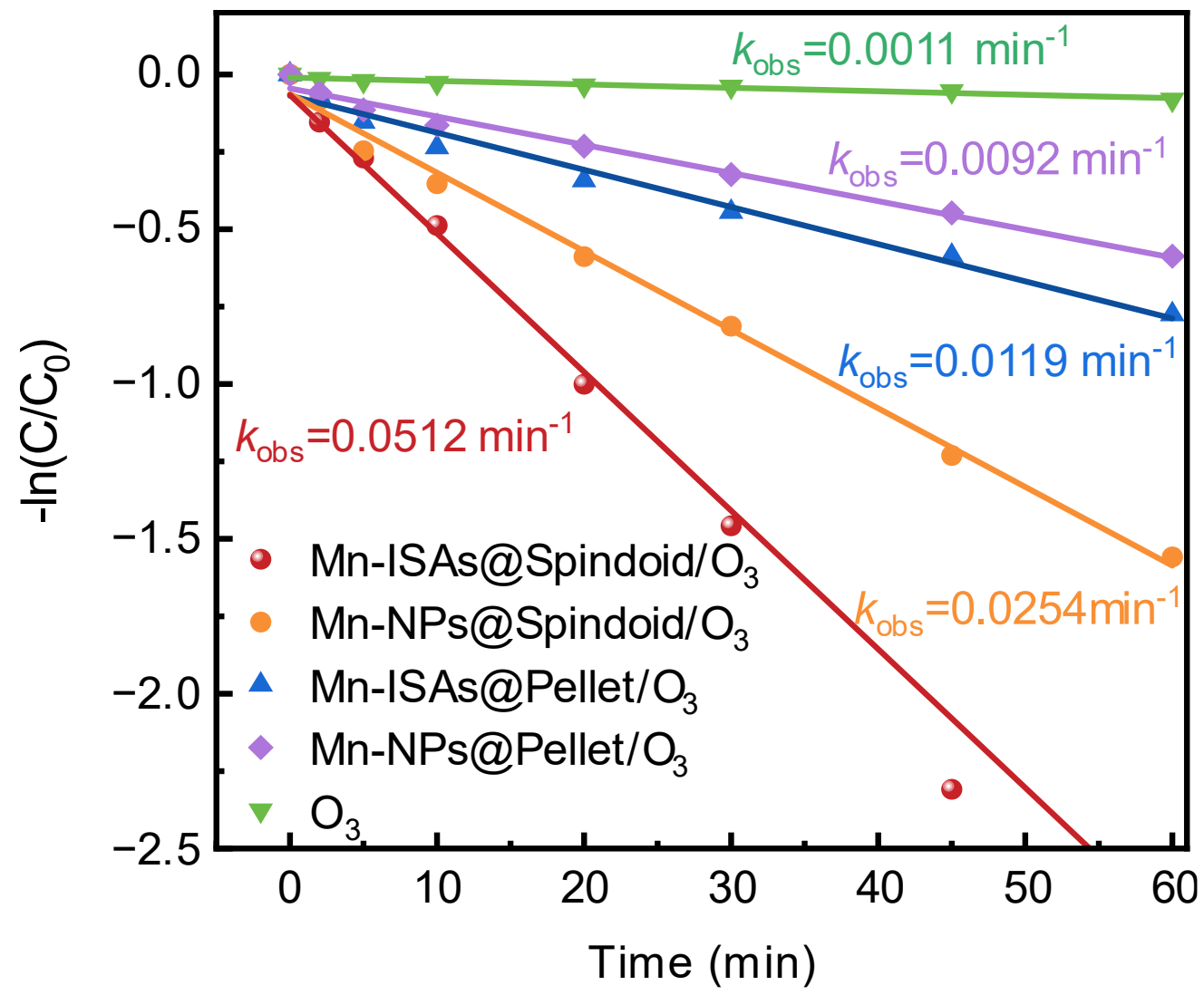

Fig. S27. First-order kinetic model of catalytic ozonation process catalyzed on different catalysts.

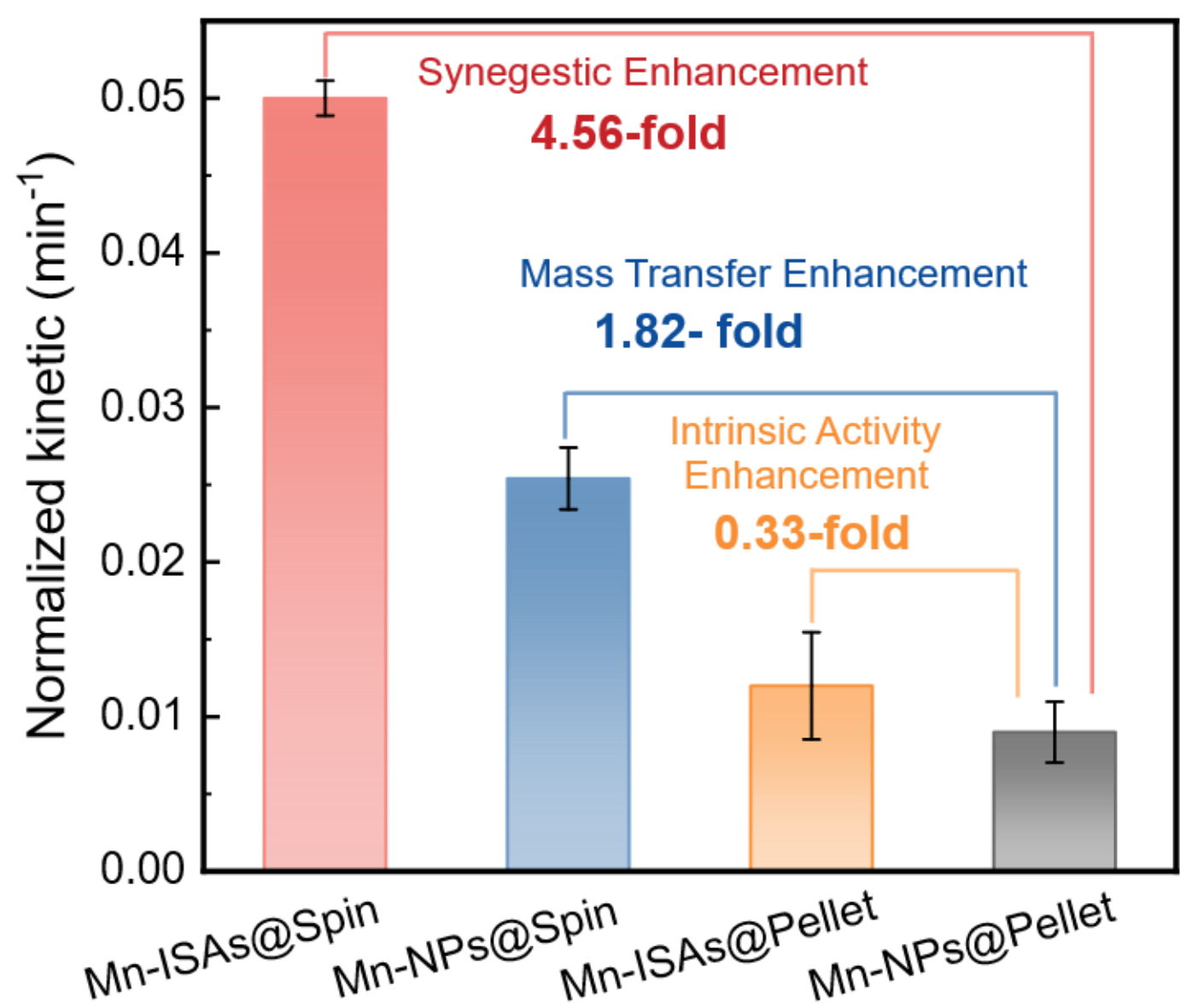

Fig. S28. Quantitative decoupling of intrinsic activity and mass-transfer contributions to catalytic performance. The intrinsic activity contribution (0.33-fold enhancement) was evaluated by comparing Mn-ISAs@Pellet with Mn-NPs@Pellet under identical architectures. The mass-transfer contribution (1.82-fold enhancement) was quantified by comparing Mn-NPs@Spinodoid with Mn-NPs@Pellet. The synergistic effect (4.56-fold enhancement) was evaluated by comparing Mn-ISAs@Spinodoid with Mn-NPs@Pellet, demonstrating a synergistically enhanced performance arising from the coupling between intrinsic site activity and architecture-regulated mass transfer.

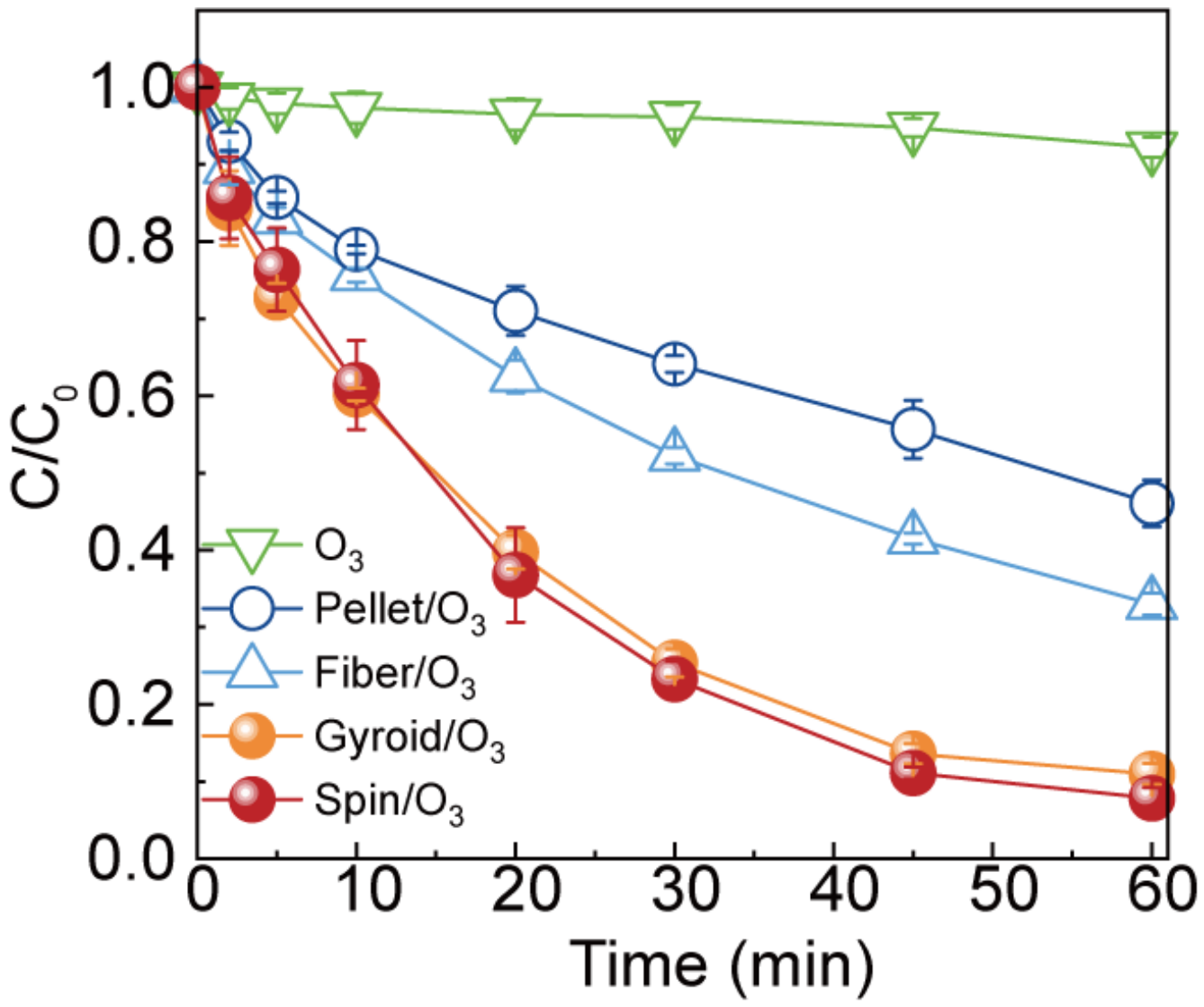

Fig. S29. OA degradation performance of bioinspired catalysts and fiber-based catalysts. Under identical packed-bed volume and operating conditions, the fiber-based catalyst also exhibits a reasonable OA degradation performance (k = 0.0187 min^{-1}). Nevertheless, its activity remains significantly lower than that of the Gyroid (k = 0.0381 min^{-1}) and Spin (k = 0.0512 min^{-1}) architected catalysts. This is attributed to the intrinsic drawbacks of fiber-based catalysts: although fibers possess more void space than pellets, they tend to agglomerate under hydraulic impact and accumulate along the reactor wall, which reduces the effective interfacial area and shortens the residence time of pollutants near the active sites.

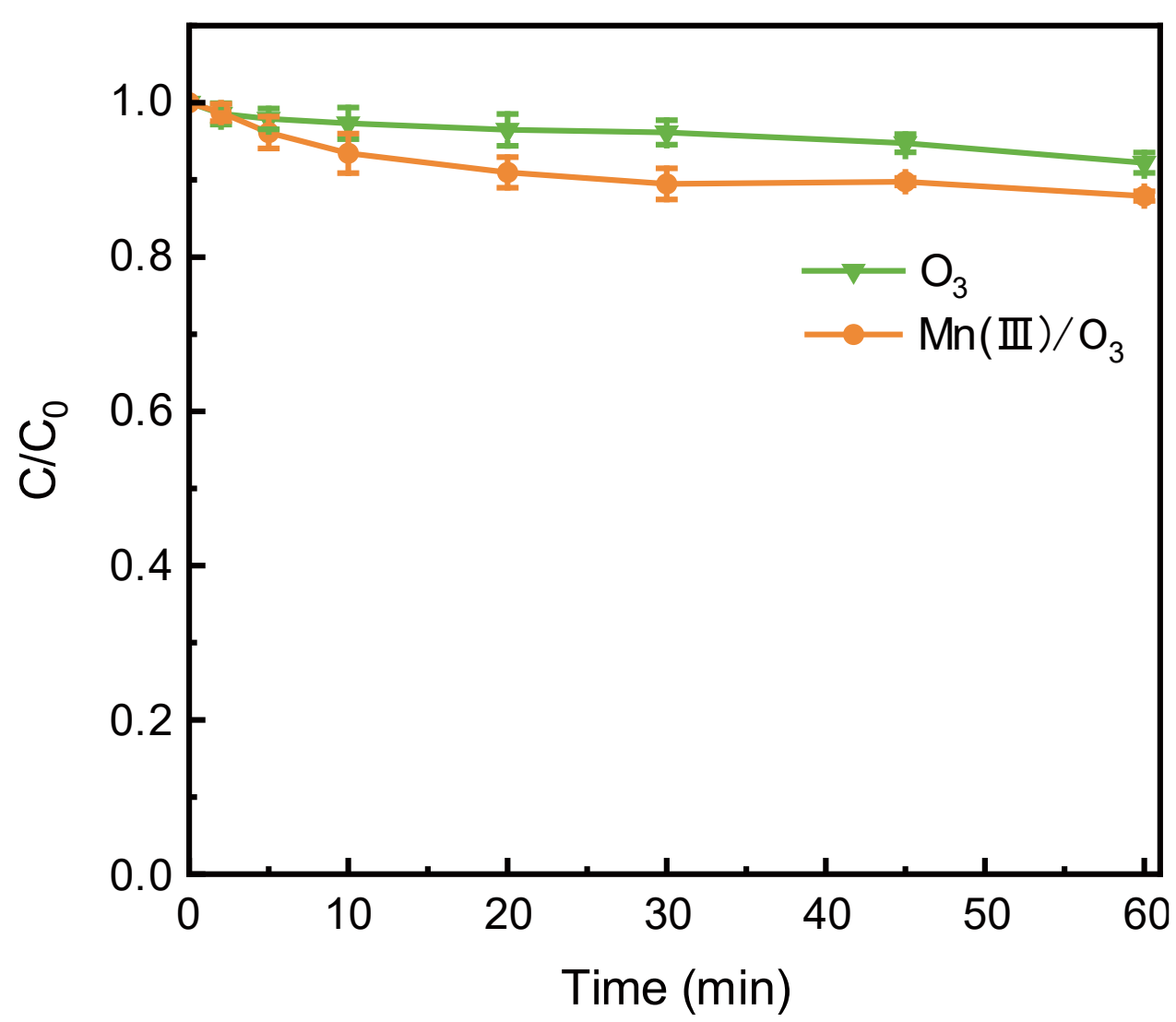

Fig. S30. OA degradation in O_3 alone system and homogeneous Mn (III) /O_3 system. Reaction conditions: $[OA]_0$ = 200 mg L^{-1}, [Mn (III)] = 0.9 mg L^{-1}, O_3 concentration = 5 mg L^{-1}, O_3 flow rate = 100 mL min^{-1}.

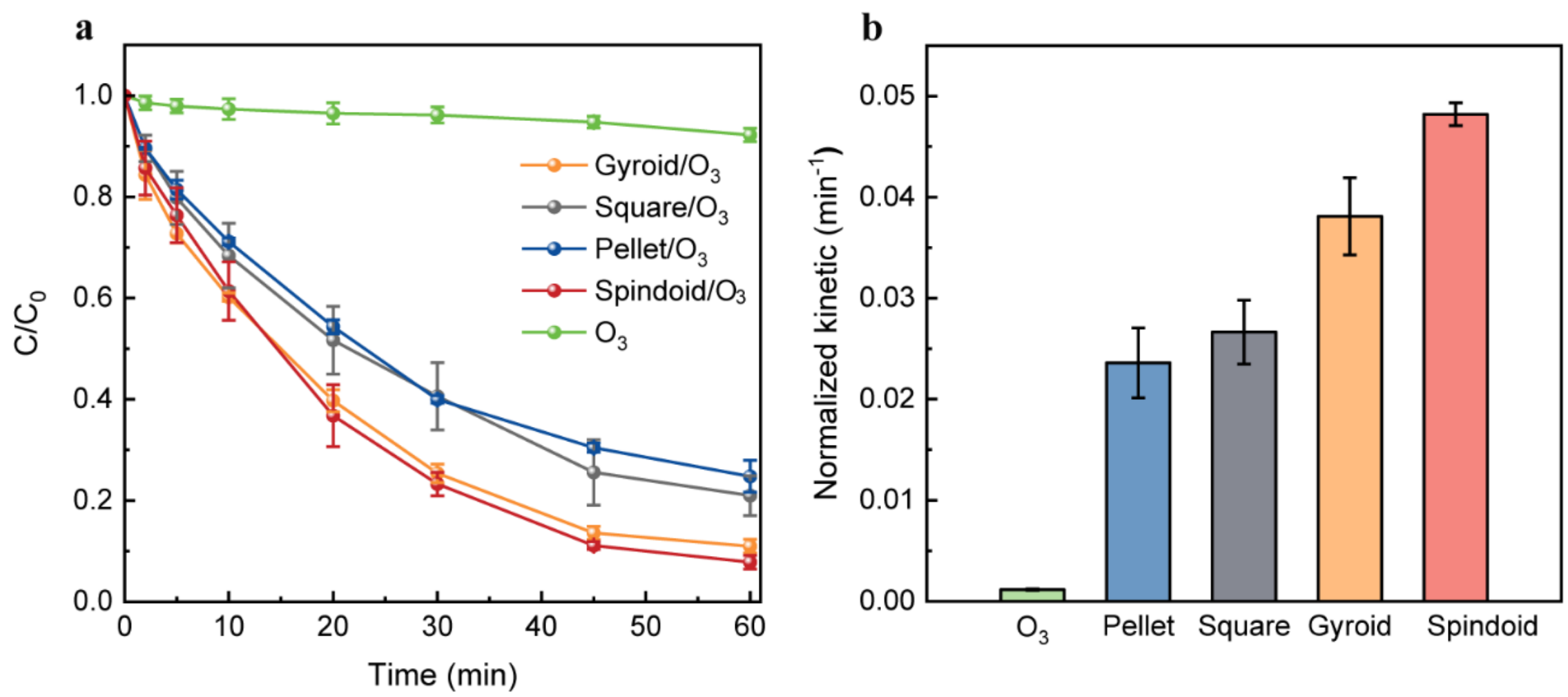

Fig. S31. OA degradation and normalized kinetics over catalysts with identical volume. a, OA degradation. **b,** Normalized kinetics.

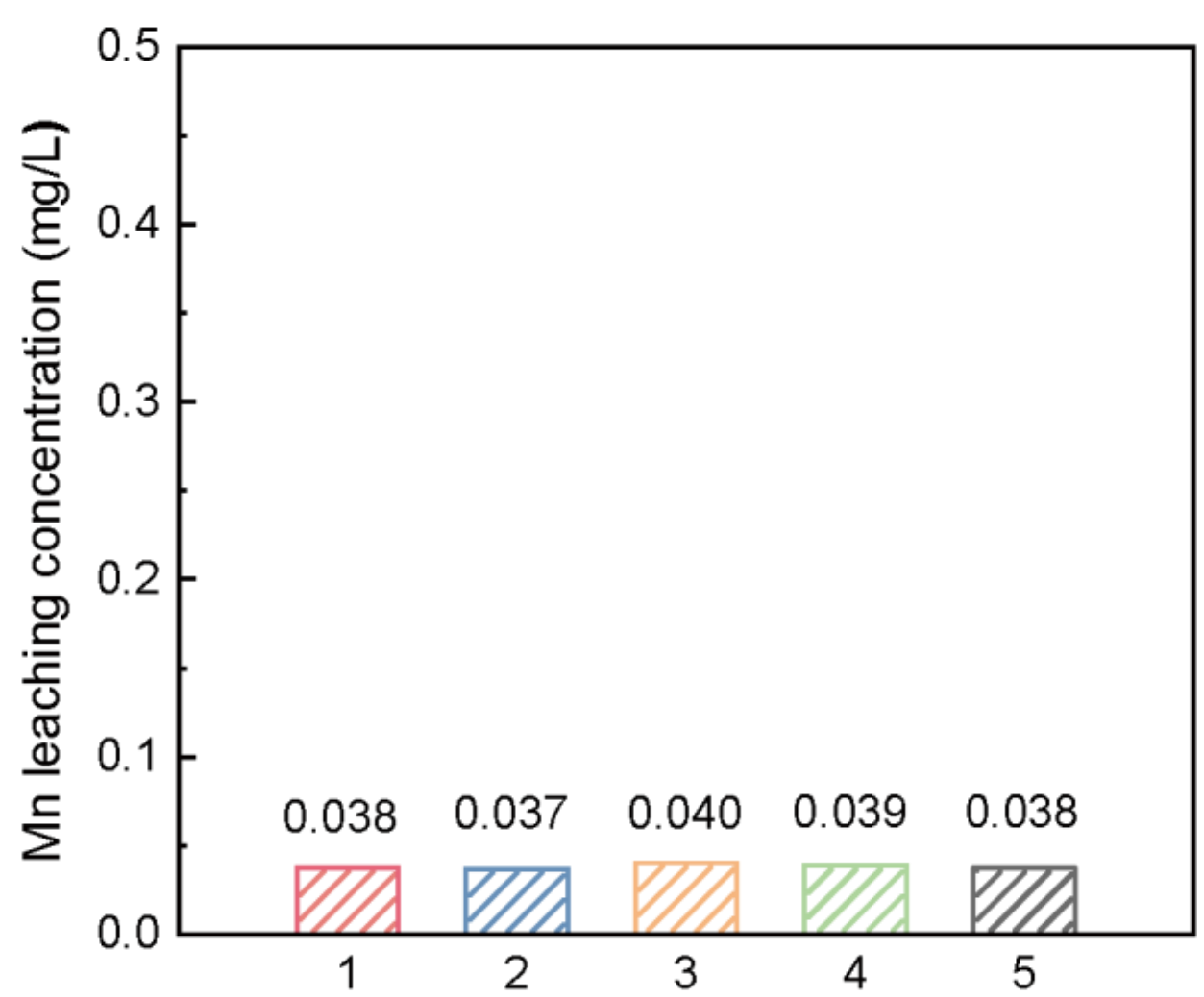

Fig. S32. The Mn leaching concentration tested using ICP-MS after successive runs of catalytic ozonation process. The Mn leaching concentration was significantly lower than the Chinese National Standard of 5 mg L^{-1} (GB 8978-1996), which is negligible for the contribution to OA degradation, showing the strong coordination at the Mn sites and no secondary pollution caused by metal leaching during catalytic ozonation process. This result indicates that the support layer and the catalytic layer anchoring the Mn active sites maintained tight integration during the reaction process, with no structural damage or collapse occurring.

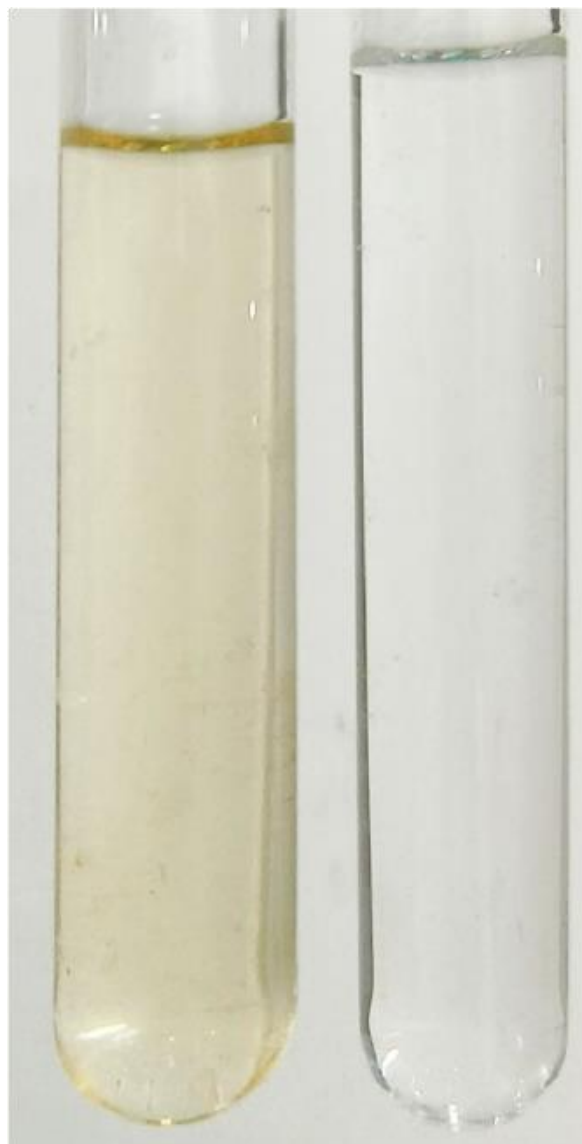

Fig. S33. Influent and effluent images of coal-chemical wastewater after ozonation treatment. The comparison images of influent(left) and effluent(right) coal-chemical wastewater after Mn-ISAs@Spinodoid ozonation treatment.

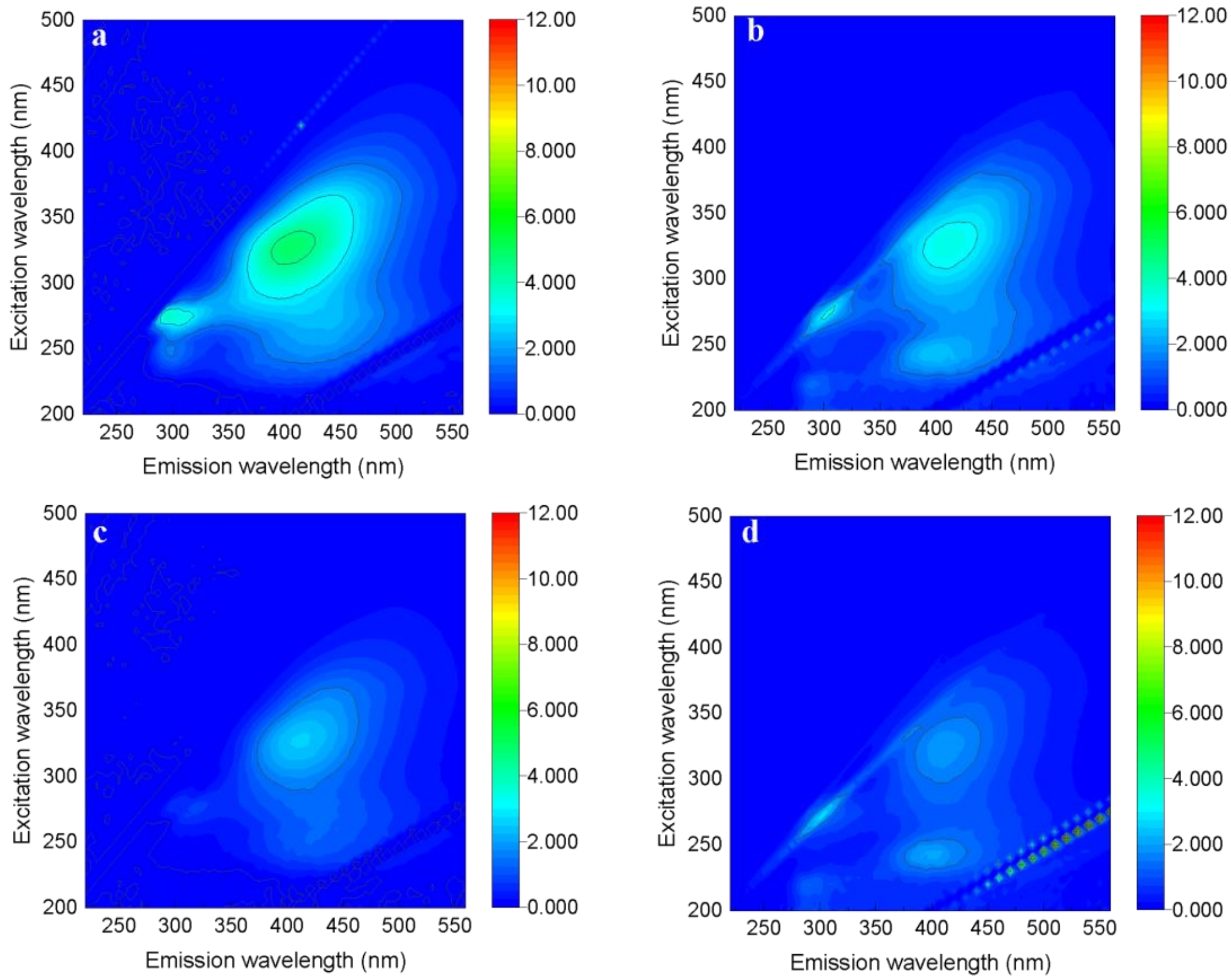

Fig. S34. 3D-EEM fluorescence spectroscopy of coal-chemical wastewater effluent with different treatment times. a, 10 min. **b,** 20 min. **c,** 30 min. **d,** 45 min. The fluorescence region integration (FRI) method divided the obtained spectra into five regions (*64*). The influent coal-chemical wastewater displayed different degrees of luminescence in the five regions, among which the intensity was the highest in Region V (humic acid-like substances, 70.1%) and Region III (fulvic acid-like substances, 12.6%). For comparison, after 60-minute ozonation treatment with Mn-ISAs@spinodoid, almost all the fluorescent macromolecules were removed, further proving its effectiveness in coal-chemical wastewater treatment.

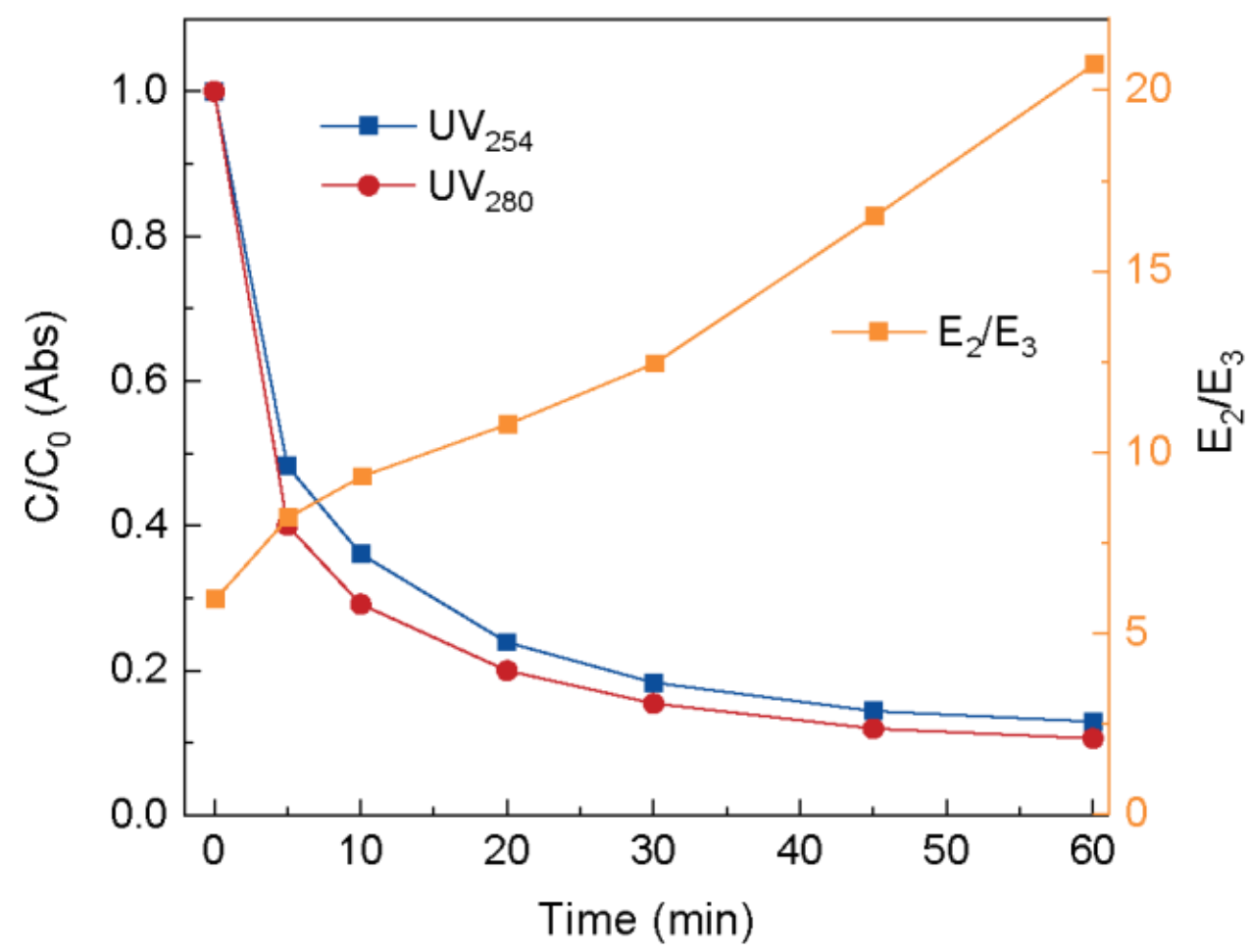

Fig. S35. UV-vis spectra and E_2/E_3 during the advanced treatment. In the UV-vis spectra, the normalized absorbance coefficients at 254 nm (UV_{254}) and 280 nm (UV_{280}) were used to represent the relative abundance of humic acid-like and proteinaceous DOM, while the change in the absorbance ratio (E_2/E_3) at 250 nm and 365 nm was inversely proportional to the relative molecular mass of DOM (*65*, *66*). The absorbance of UV_{254} and UV_{280} exhibited a sharp decrease during the catalytic ozonation with Mn-ISAs@spinodoid, while the value of E_2/E_3 continued to increase, which fully indicated that macromolecular DOM substances such as aromaticity were degraded into environmentally friendly micromolecular.

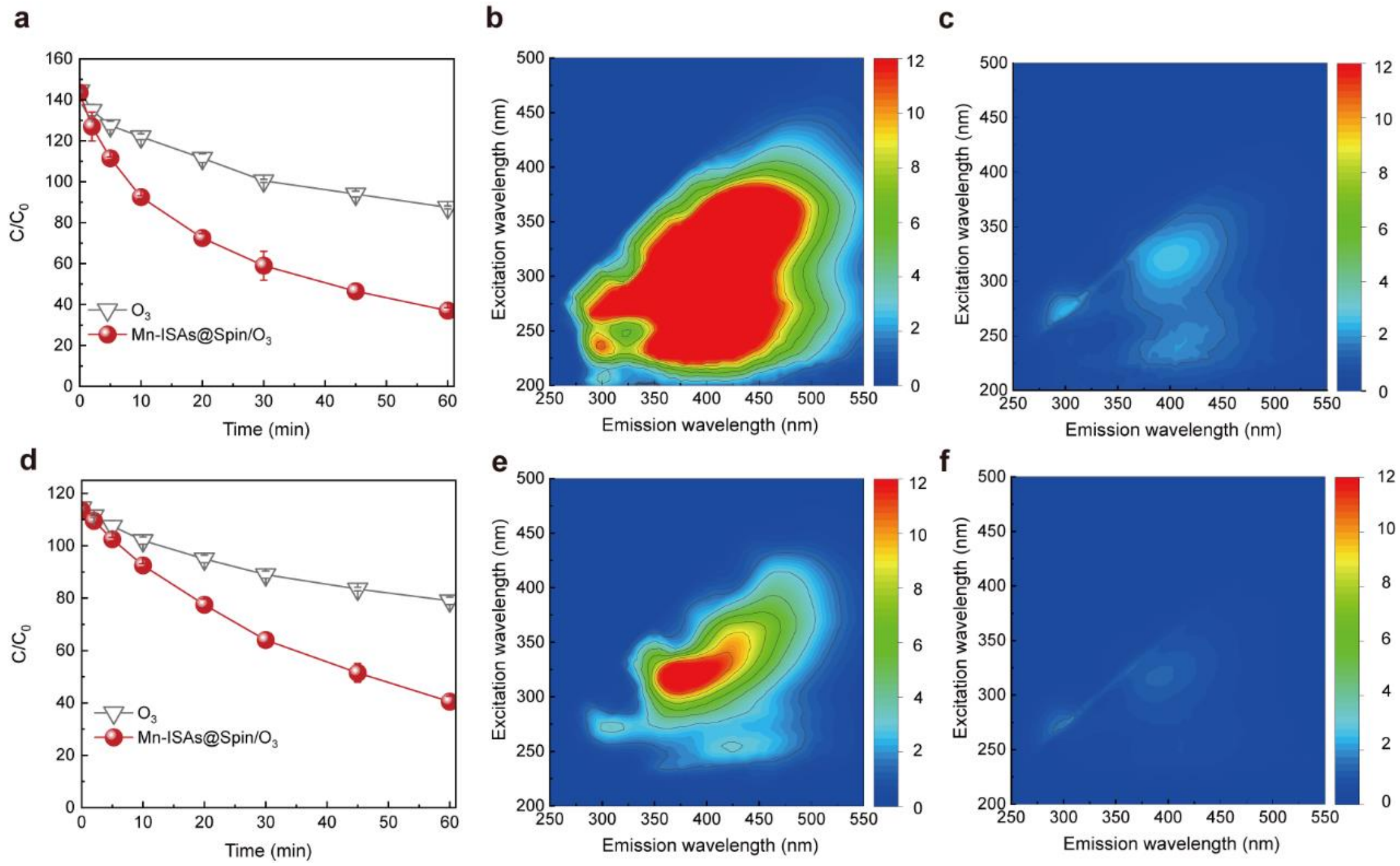

Fig. S36. COD degradation and EEM characterization of petroleum and pharmaceutical wastewaters. a, COD degradation in petroleum wastewater. **b-c,** EEM images of influent and effluent of petroleum wastewater. **d,** COD degradation in pharmaceutical wastewater. **e-f,** EEM images of influent and effluent of pharmaceutical wastewater.

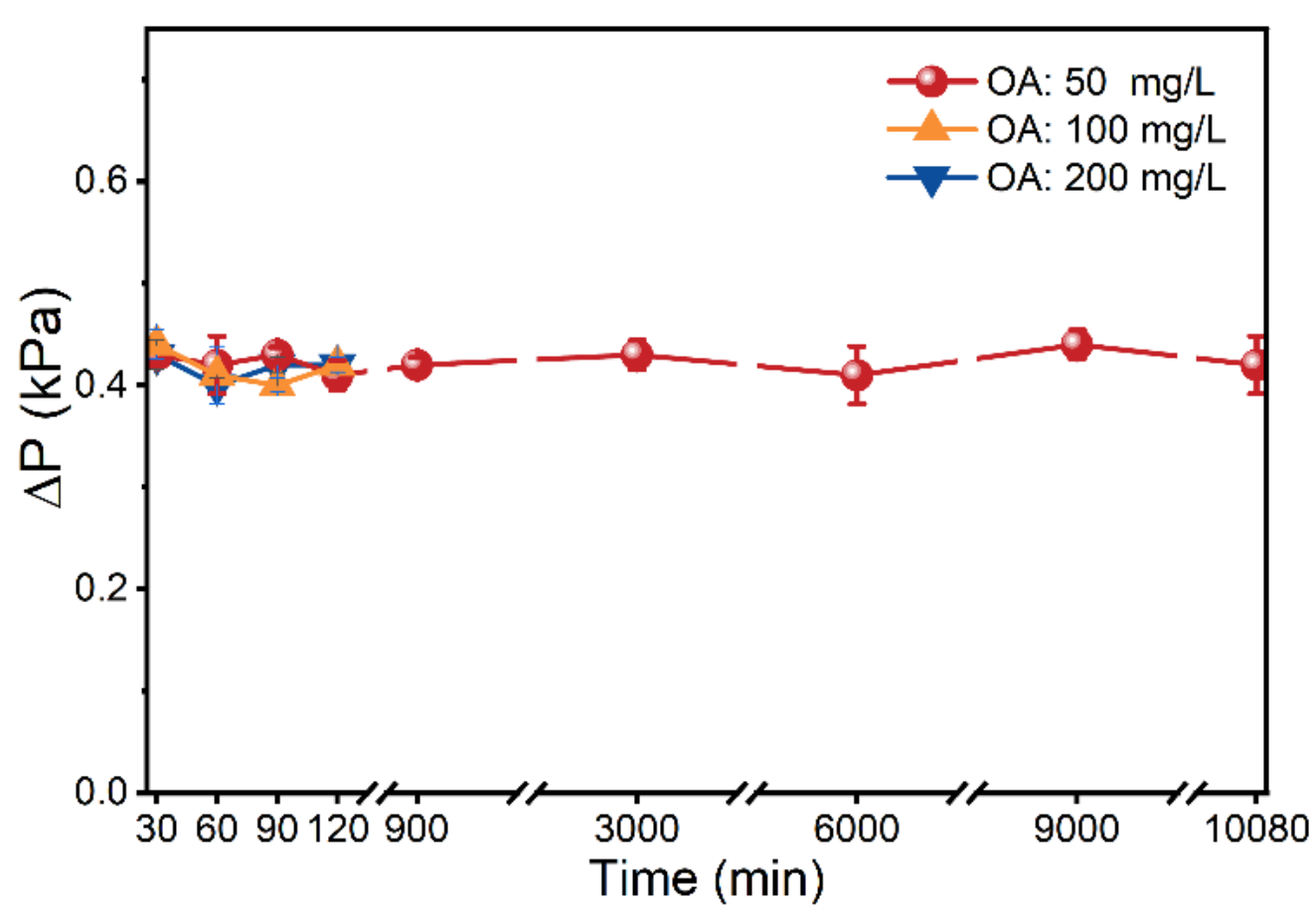

Fig. S37. Pressure-drop measurements of Mn-ISAs@Spinodoid during oxalic acid degradation at different wastewater concentrations and operation durations. The measurements were performed at influent concentrations of 50, 100, and 200 mg $L^{-1}$with operation times of 30, 60, 90, and 120 min. The data point at 900, 3000, 6000, 9000 and 10080 min corresponds to the pressure drop measured using the catalyst at 100 mg L^{-1}. The pressure drop remains nearly constant at approximately 0.42 kPa under all tested conditions, indicating negligible flow resistance increase, good resistance to fouling, and excellent structural stability of the architected catalyst under long-term hydraulic conditions.

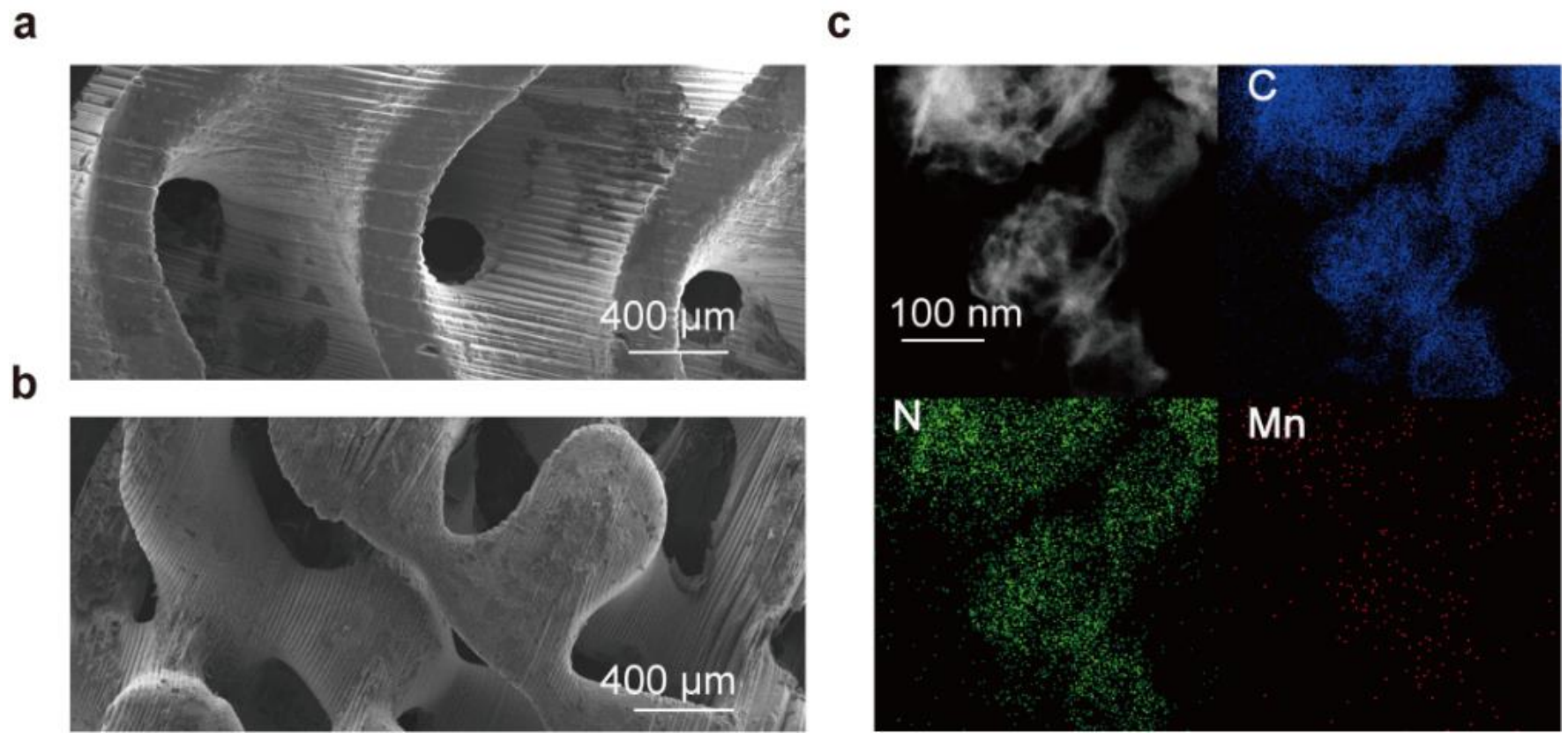

Fig. S38. SEM/TEM images and elemental mapping of used Mn-ISAs@AAF catalysts. a-b, SEM images of the used Mn-ISAs@AAF catalysts. **c,** TEM and element mapping images of used Mn-ISAs@AAF catalysts.

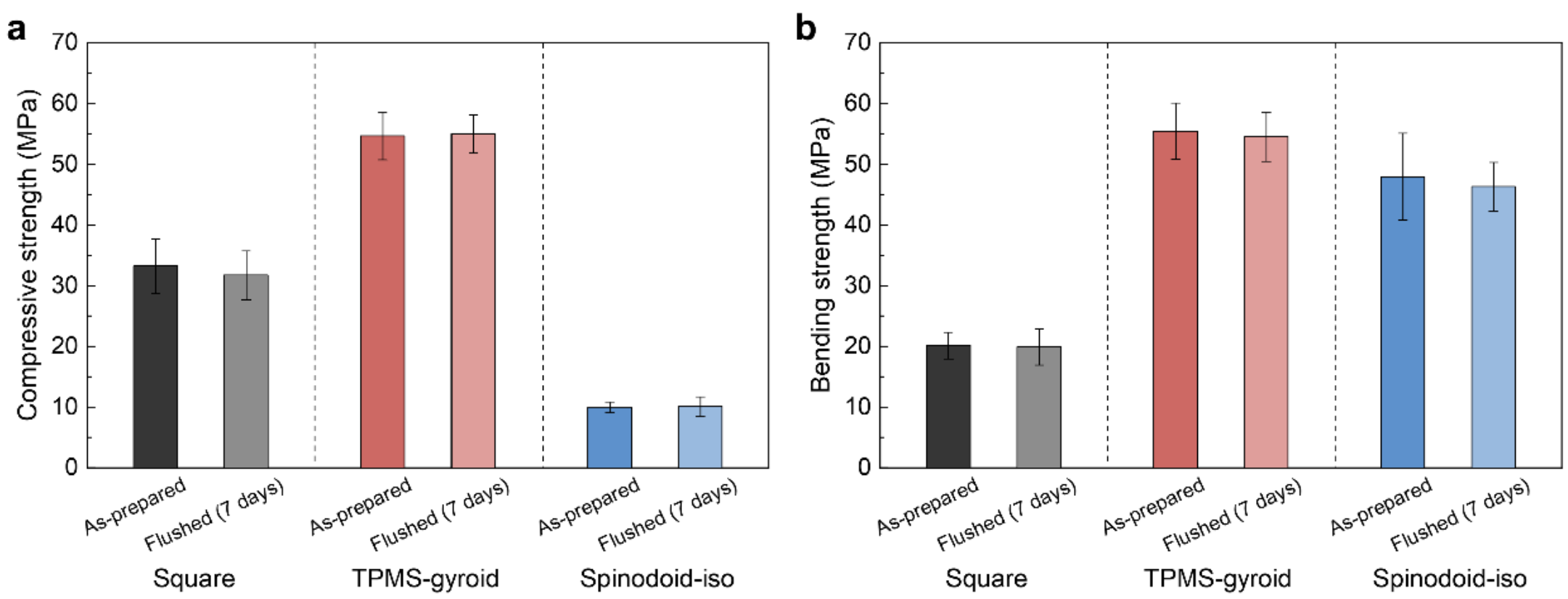

Fig. S39. Comparisons of the mechanical properties of the Square, TPMS-gyroid, and Spinodoid-iso catalyst frameworks before and after flushing. a, Compressive strength. **b**, Bending strength. The results show that after 168 hours of aqueous exposure, the compressive and bending strengths of the three architectures remained comparable to those obtained under dry conditions prior to flushing. This confirms that long-term water flow does not result in discernible mechanical degradation of these catalyst frameworks.

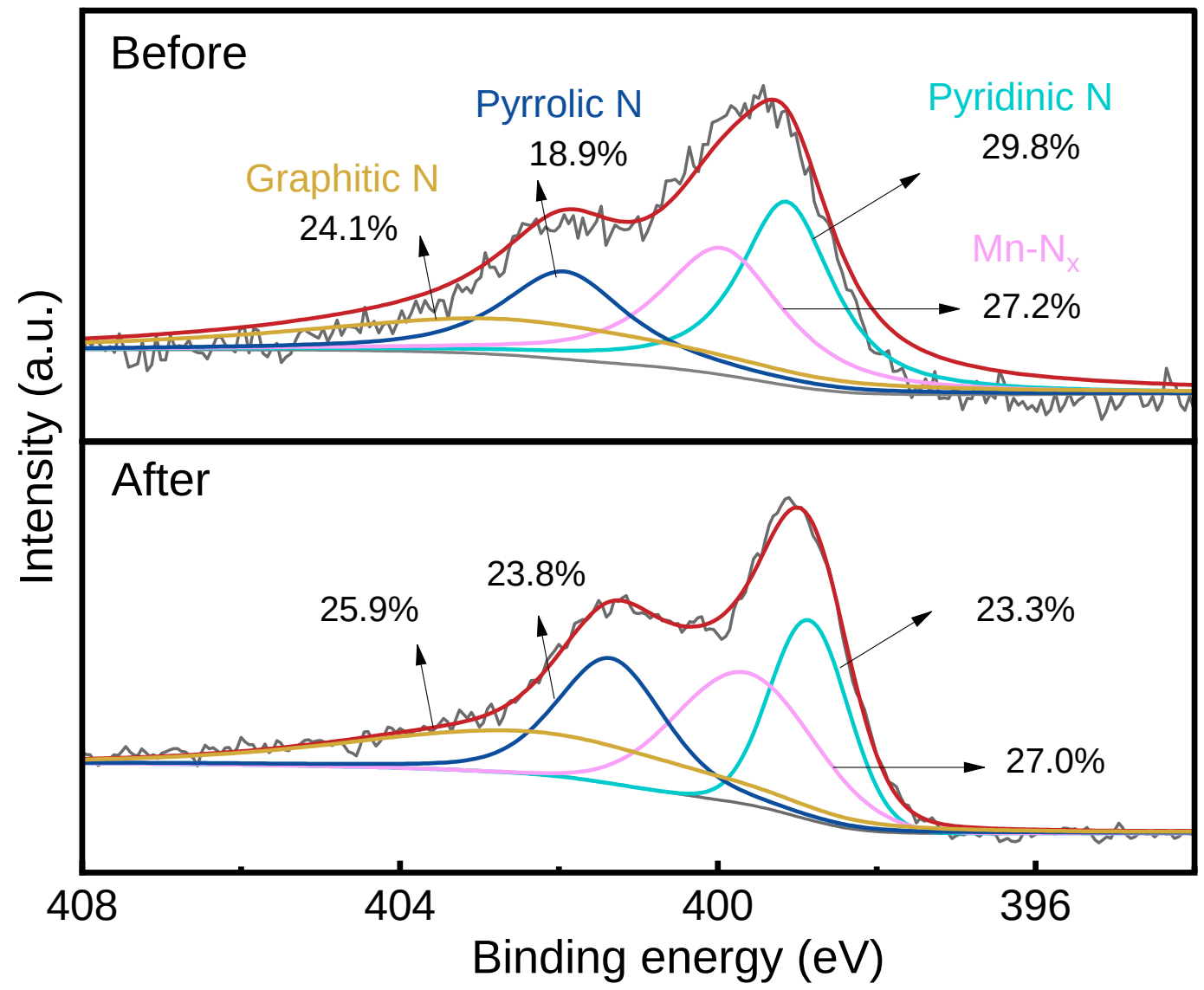

Fig. S40. N 1s XPS spectra of Mn-ISAs@AAF catalysts before and after prolonged use.

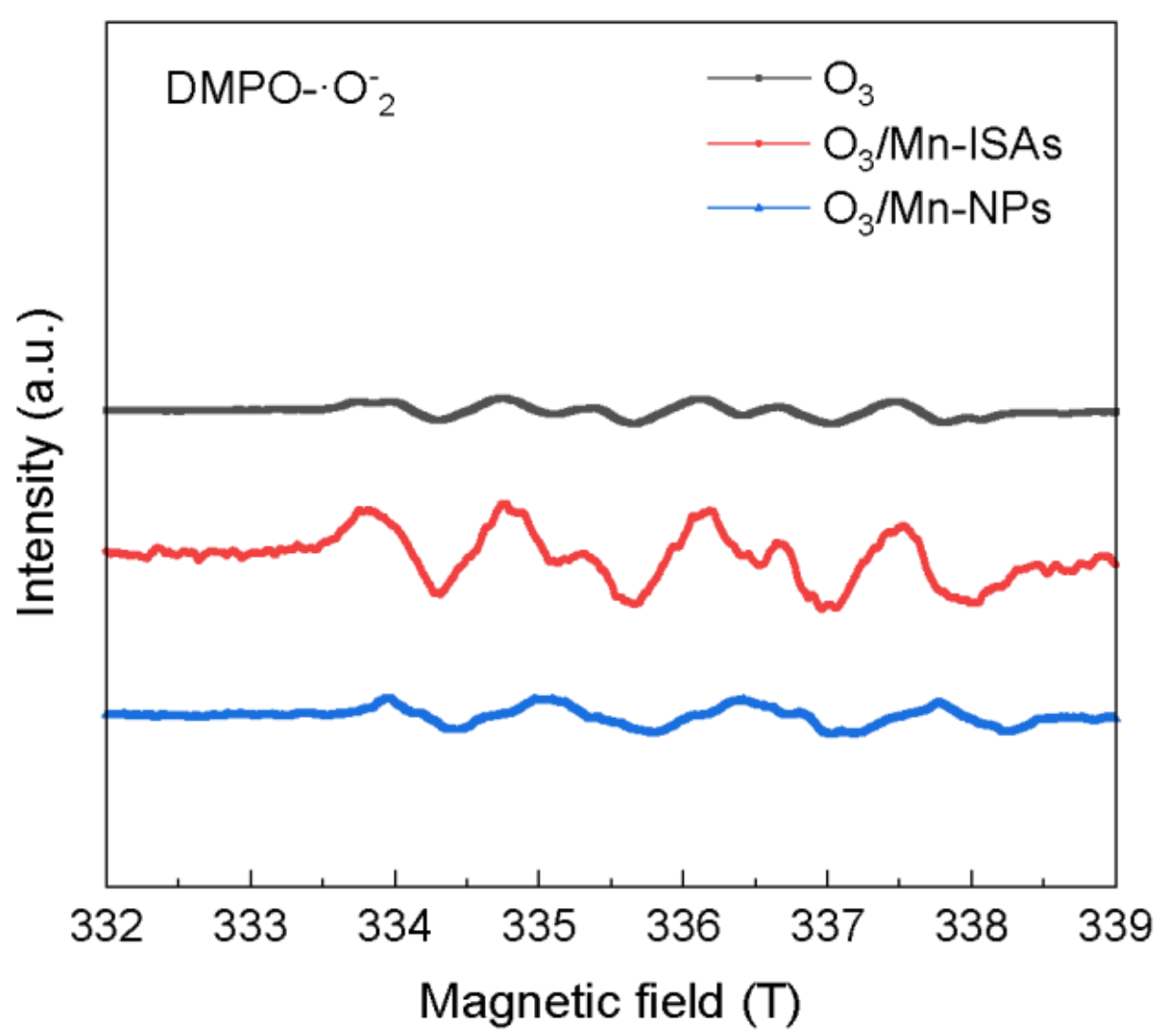

Fig. S41. EPR spectra of • O_2^- in different systems. There were more distinct peak patterns in the O_3/Mn-ISAs system.

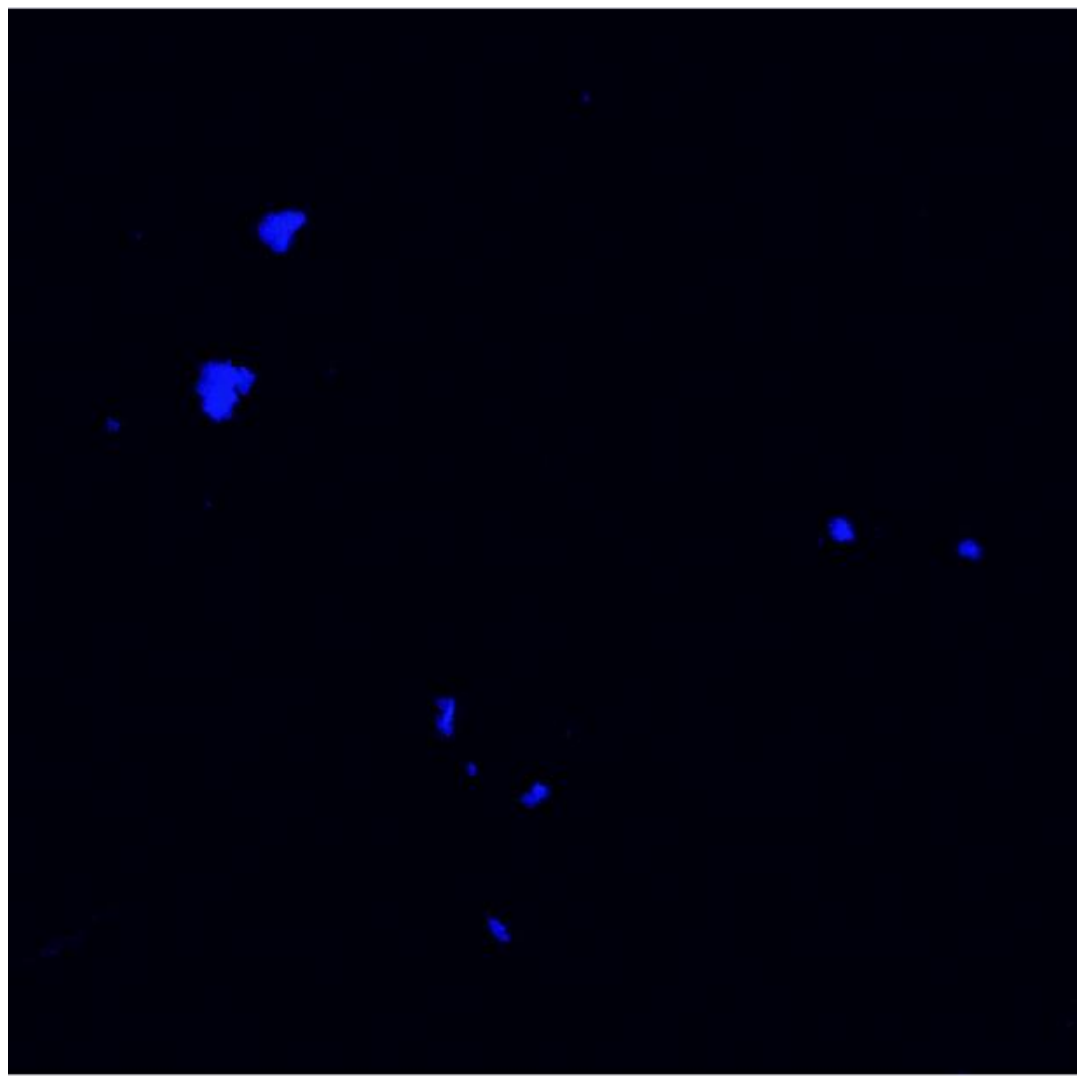

Fig. S42. •OH detection on Mn-ISAs@AAF by laser scanning confocal microscopy. Fluorescence images of Mn-ISAs@AAF catalyst with coumarin added, taken by a laser scanning confocal microscope, further confirmed the generation of surface absorbed •OH. The detected signal of 7-hydroxycoumarin indicated that the coumarin probe successfully bound to •OH on the Mn-ISAs@AAF surface (*67*).

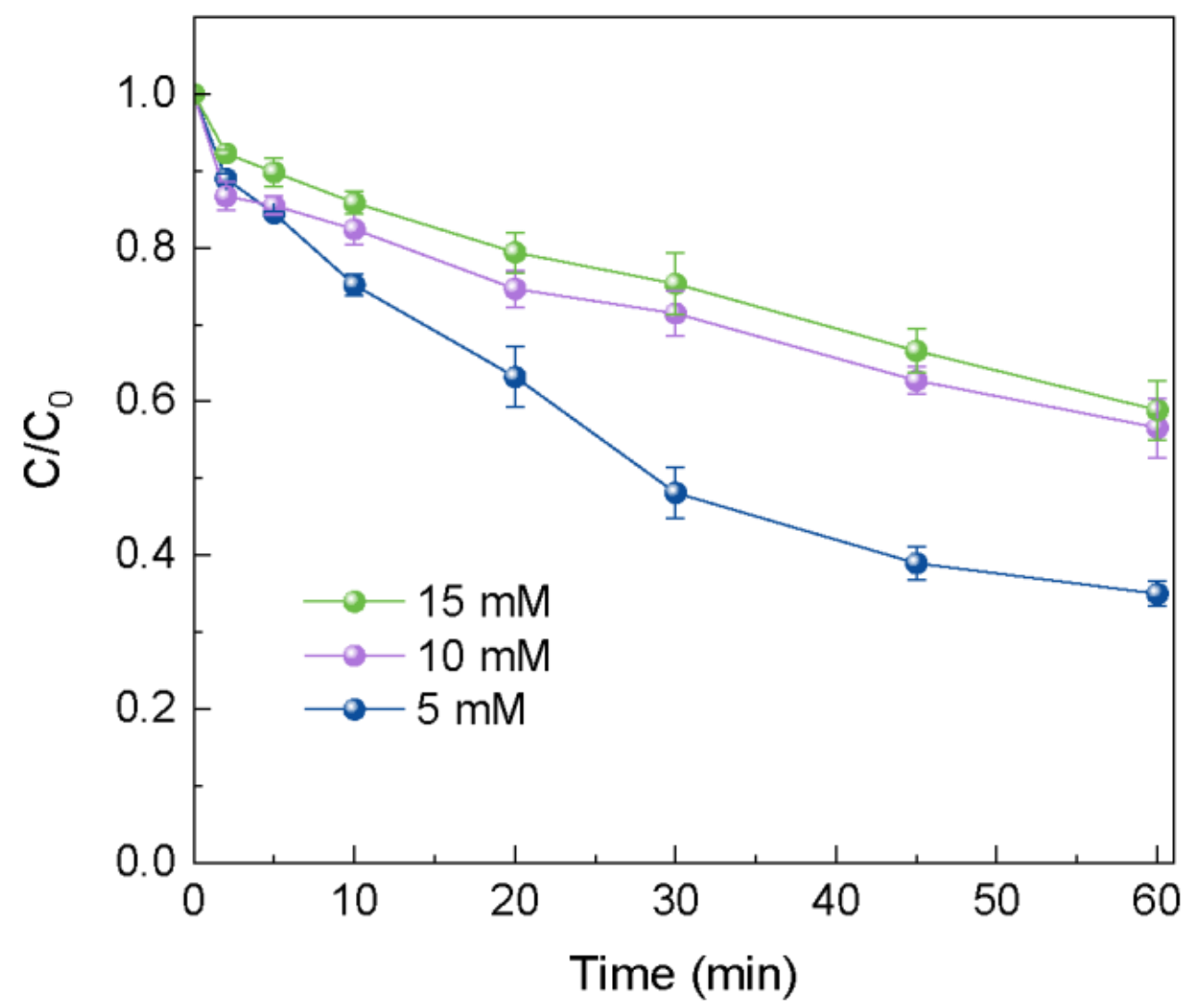

Fig. S43. Effect of different concentration of quenching agent (methanol) on the degradation of OA.

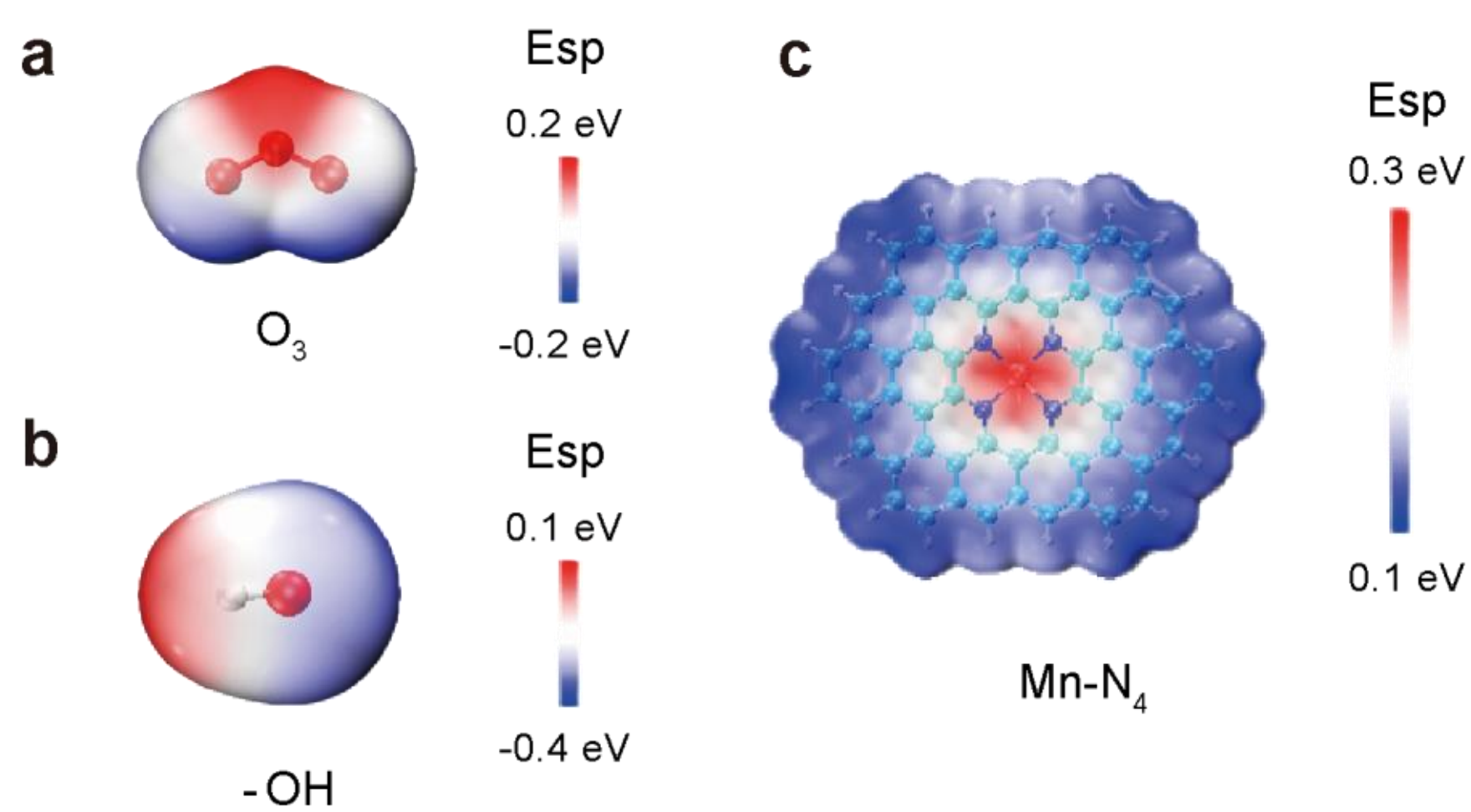

Fig. S44. ESP maps of O_3, OH^- and carbon skeleton containing the Mn-N_4 moiety. a, O_3. **b,** OH^-. **c,** Carbon skeleton containing the Mn-N_4 moiety. According to the Pauling electronegativity scale, N had a higher electronegativity value (3.04) and a stronger attraction for electrons than Mn (1.55), which made manganese atom had the highest charge and the lowest electron density of the carbon skeleton, very susceptible to attack by electron-rich groups. The terminal O atoms of ozone molecular and hydroxide ions were both negatively charged, preferring to bind with Mn single-atom site with low electron density to trigger the chain reactions.

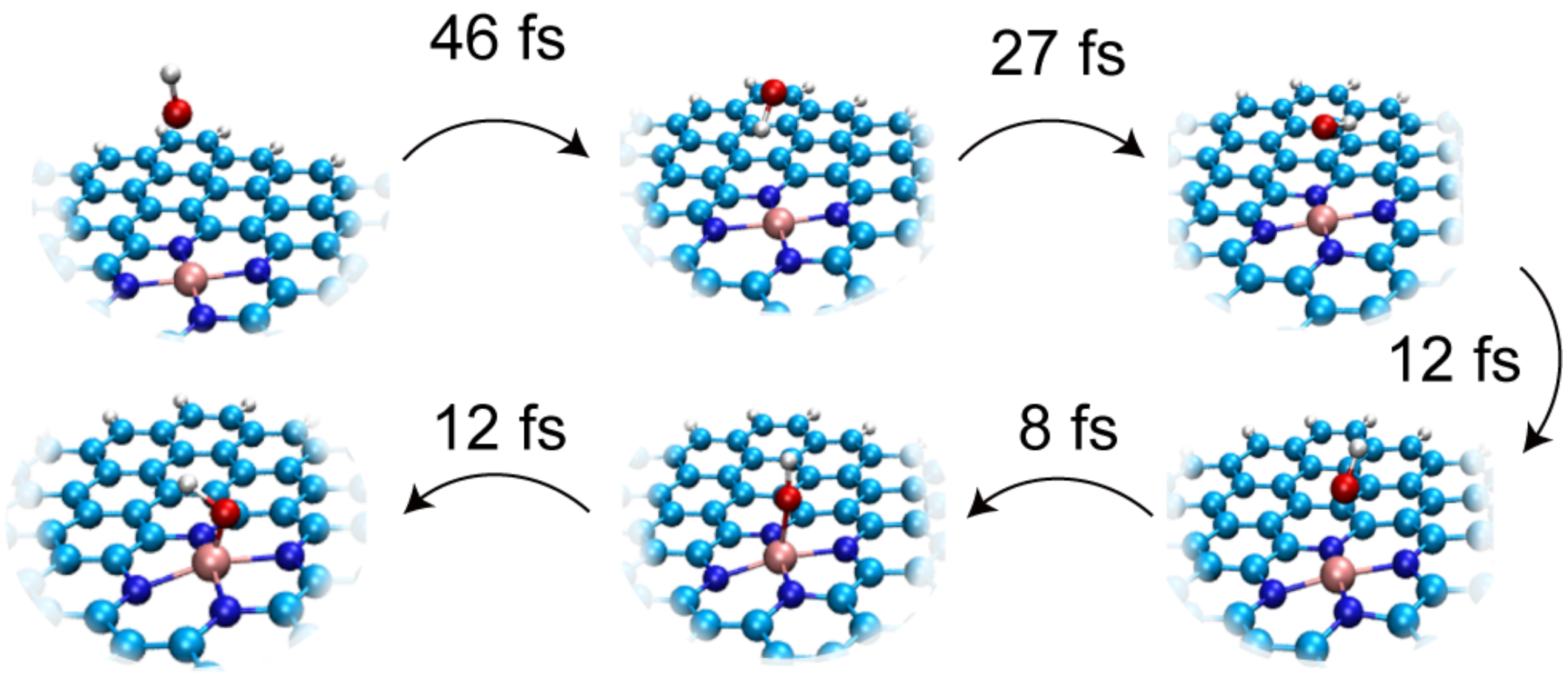

Fig. S45. The entire reaction process between MnN_4 and OH^- simulated by AIMD. The final reaction resulted in the formation of the MnN_4-OH structure.

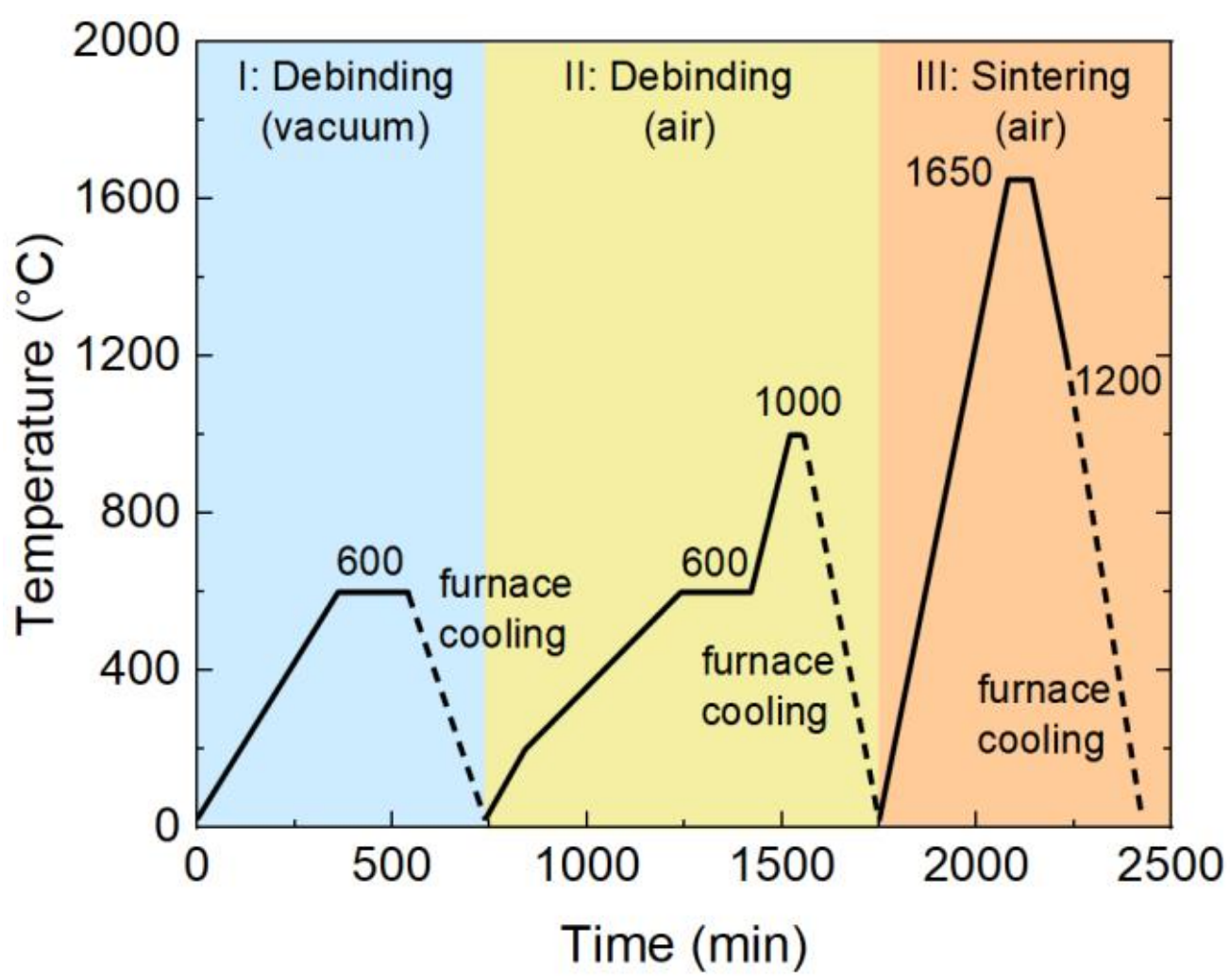

Fig. S46. Heat treatment process for debinding and sintering of AAFs. After three stages of sintering, the AFF structure was finally obtained.

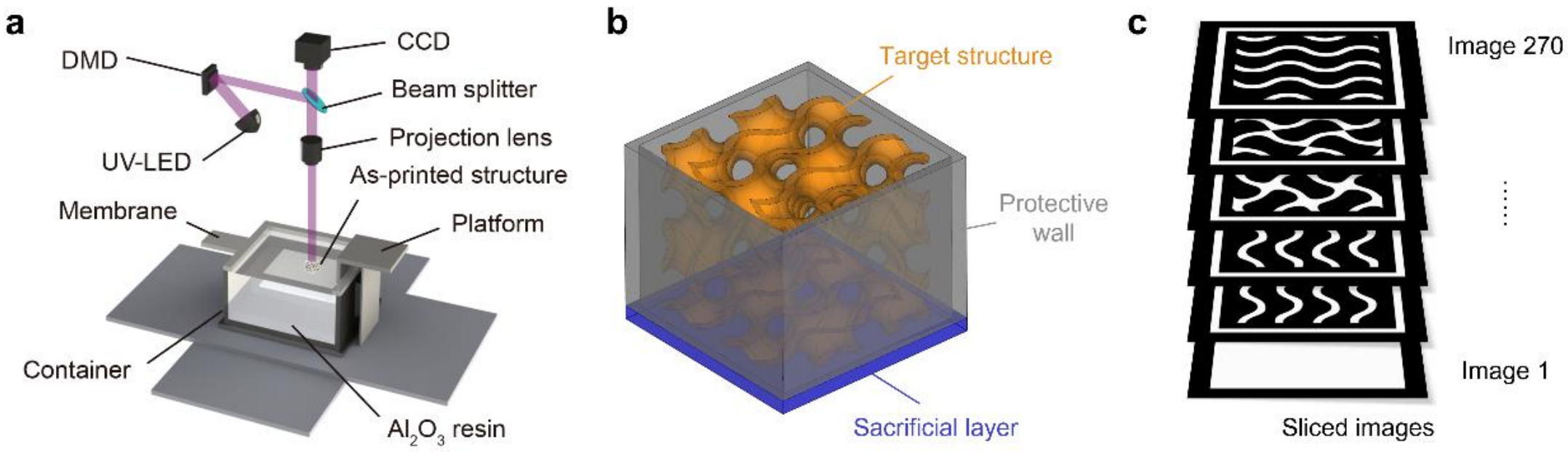

Fig. S47. Schematic illustration of the DLP printing of ceramic structures. a, Principle of bottom-up DLP printing. **b,** Components of the printed construct, including the target structure, sacrificial layer, and protective wall. **c,** Example slicing images used for layer-by-layer DLP printing.

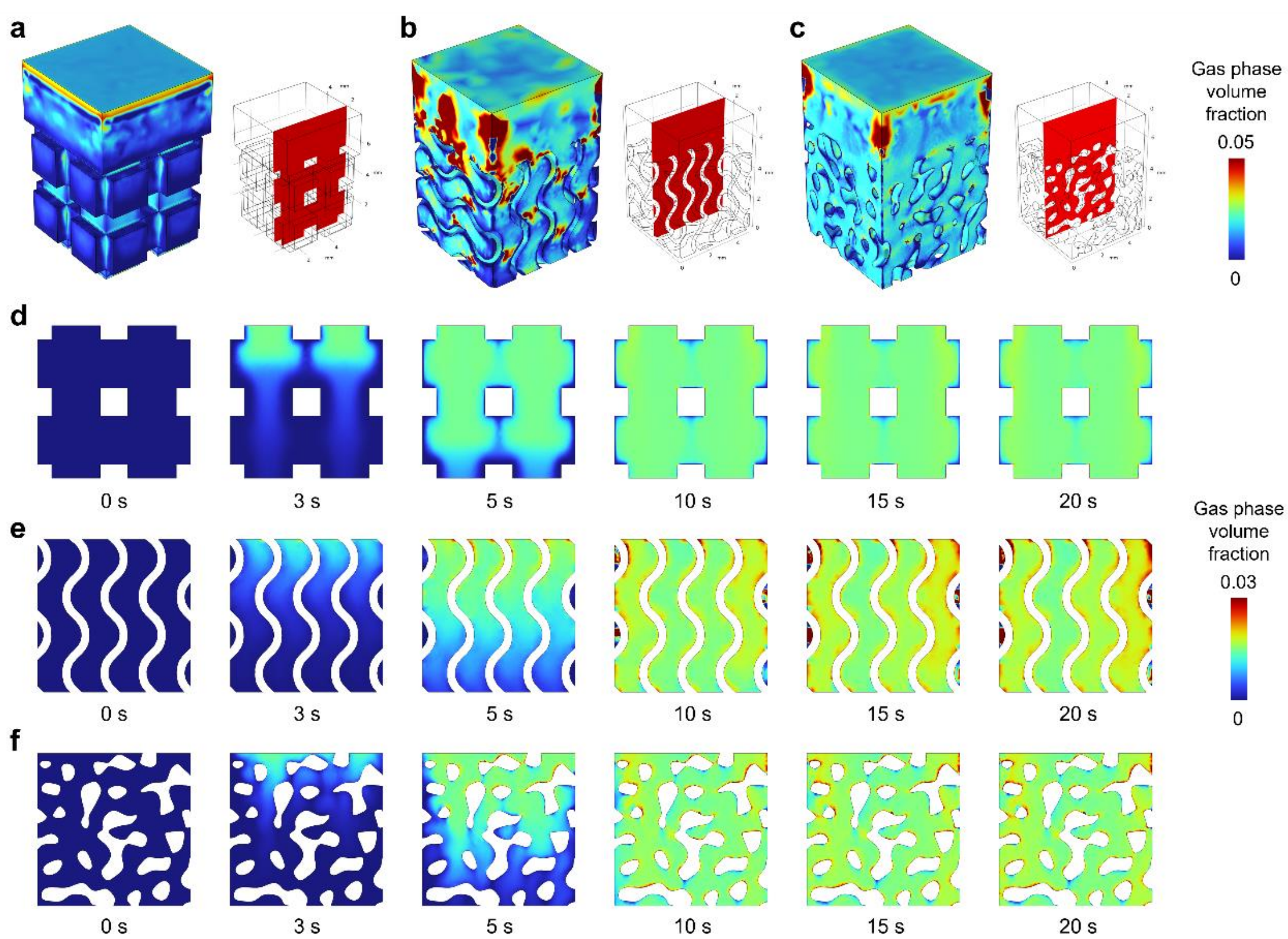

Fig. S48. Simulated time-dependent behavior of the bubble flow (laminar). a–c, Gas phase (ozone) volume fraction and cross-sectional views within the a) Square, b) TPMS-gyroid, and c) Spinodoid-iso catalysts at 20 s. **d–f,** Evolution of the gas-phase volume fraction distribution across the cross sections of d) Square, e) TPMS-gyroid, and f) Spinodoid-iso catalysts.

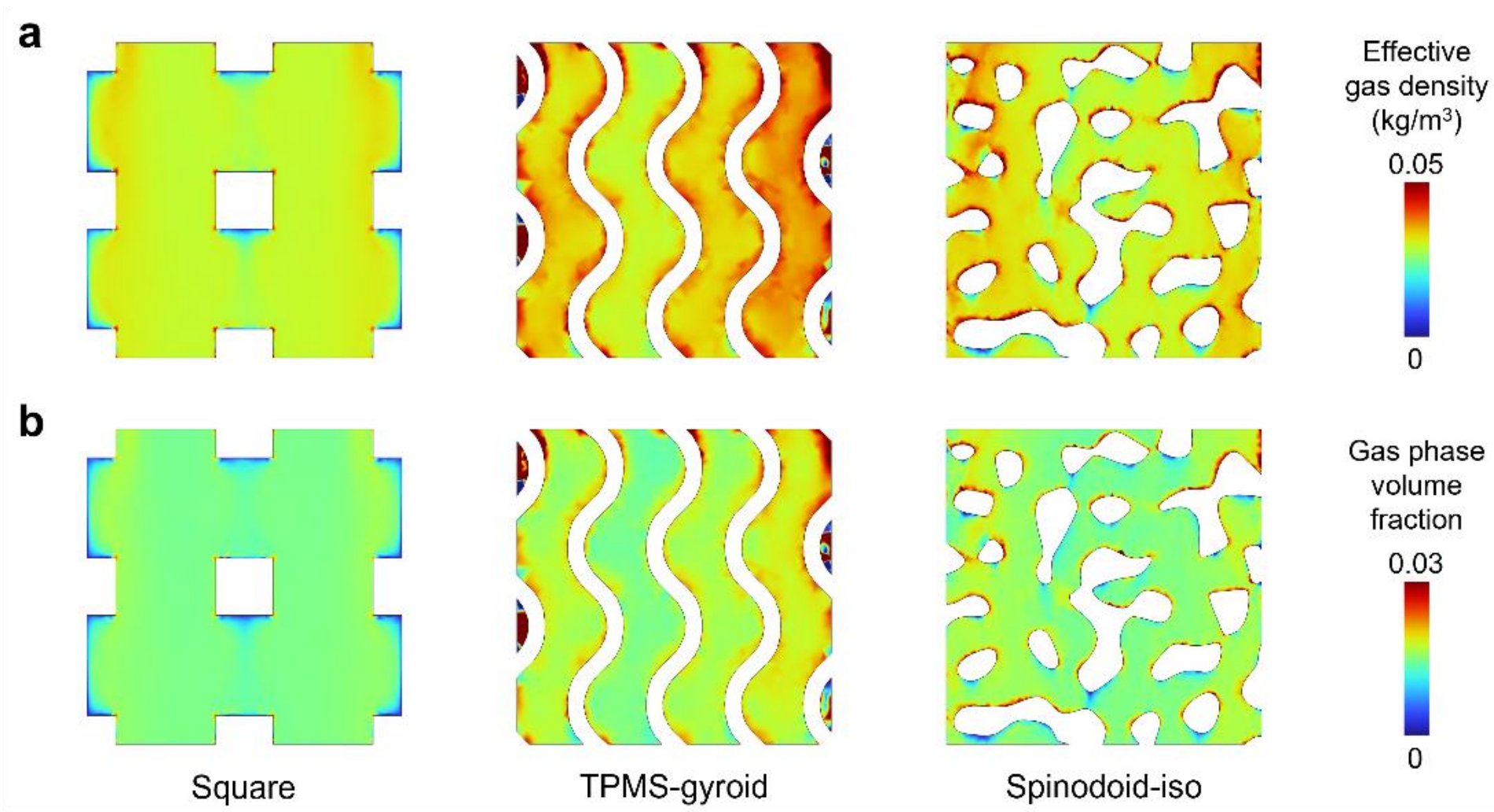

Fig. S49. Simulated gas-phase distribution within the Square, TPMS-gyroid, and Spinodoid-iso catalysts. a, Effective gas density. **b,** Gas-phase volume fraction.

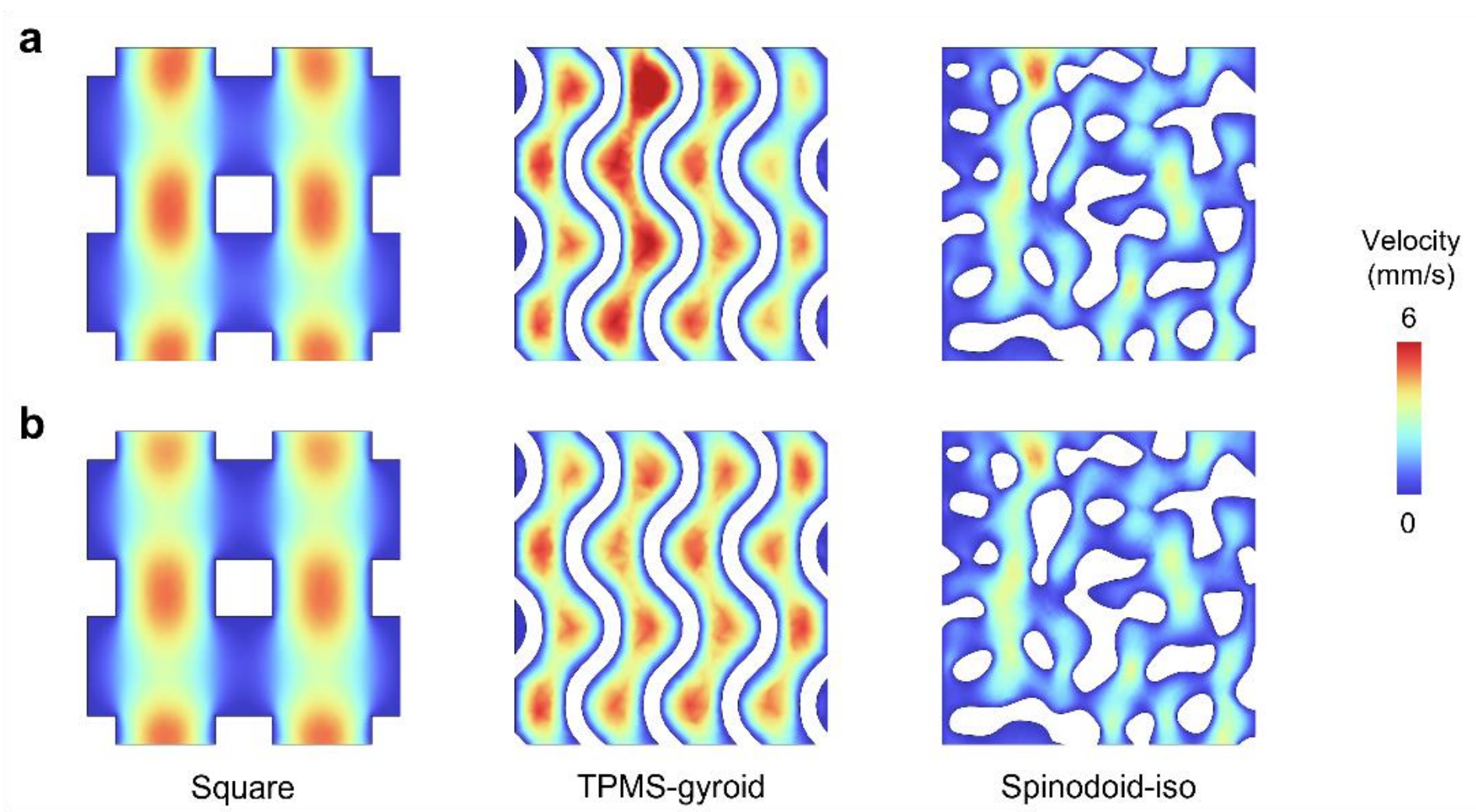

Fig. S50. Simulated fluid velocity distributions within the Square, TPMS-gyroid, and Spinodoid-iso catalysts. a, Bubble flow (laminar). **b,** Laminar flow only.

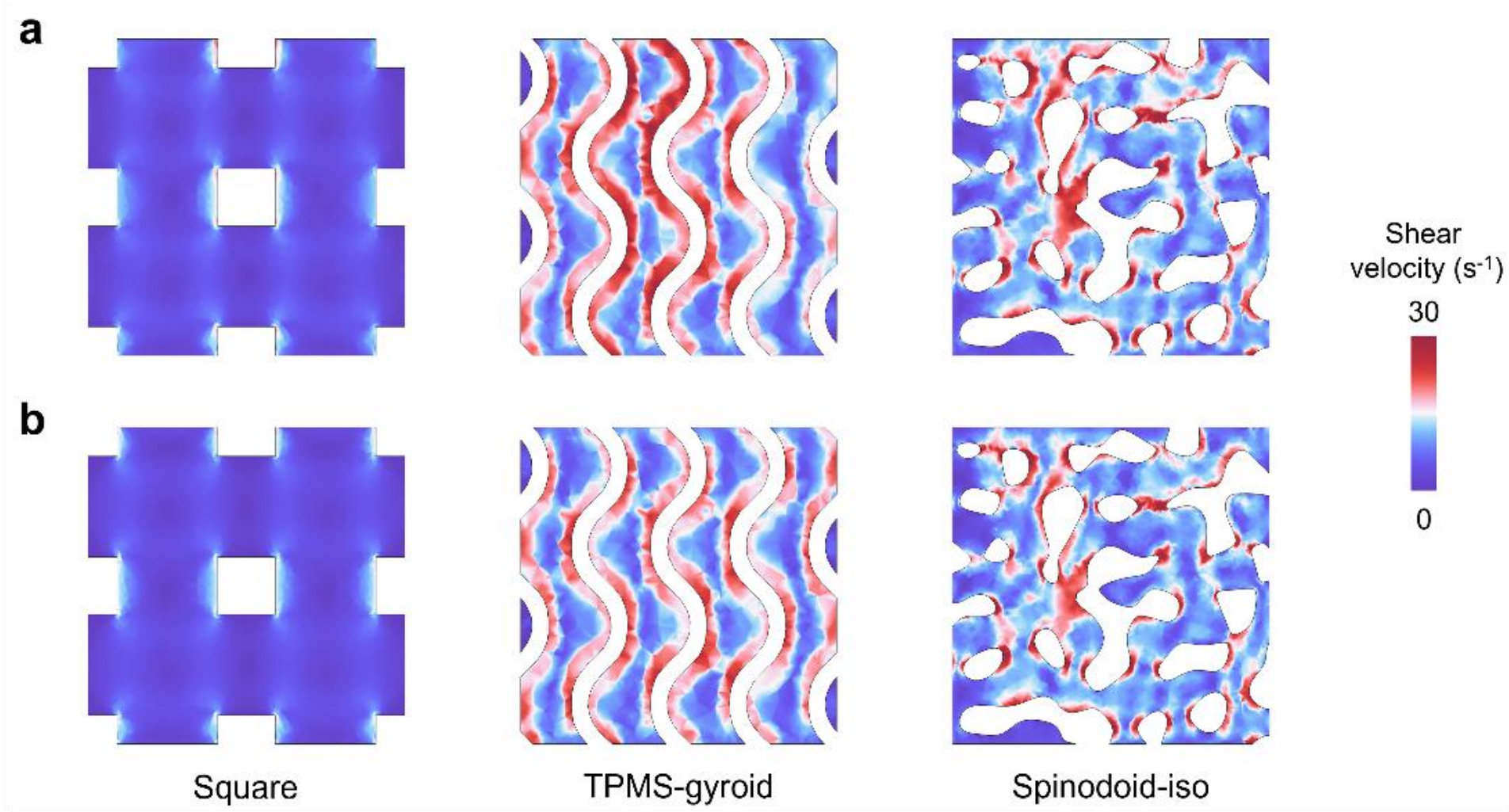

Fig. S51. Simulated shear velocity distributions within the Square, TPMS-gyroid, and Spinodoid-iso catalysts. a, Bubble flow (laminar). **b,** Laminar flow only.

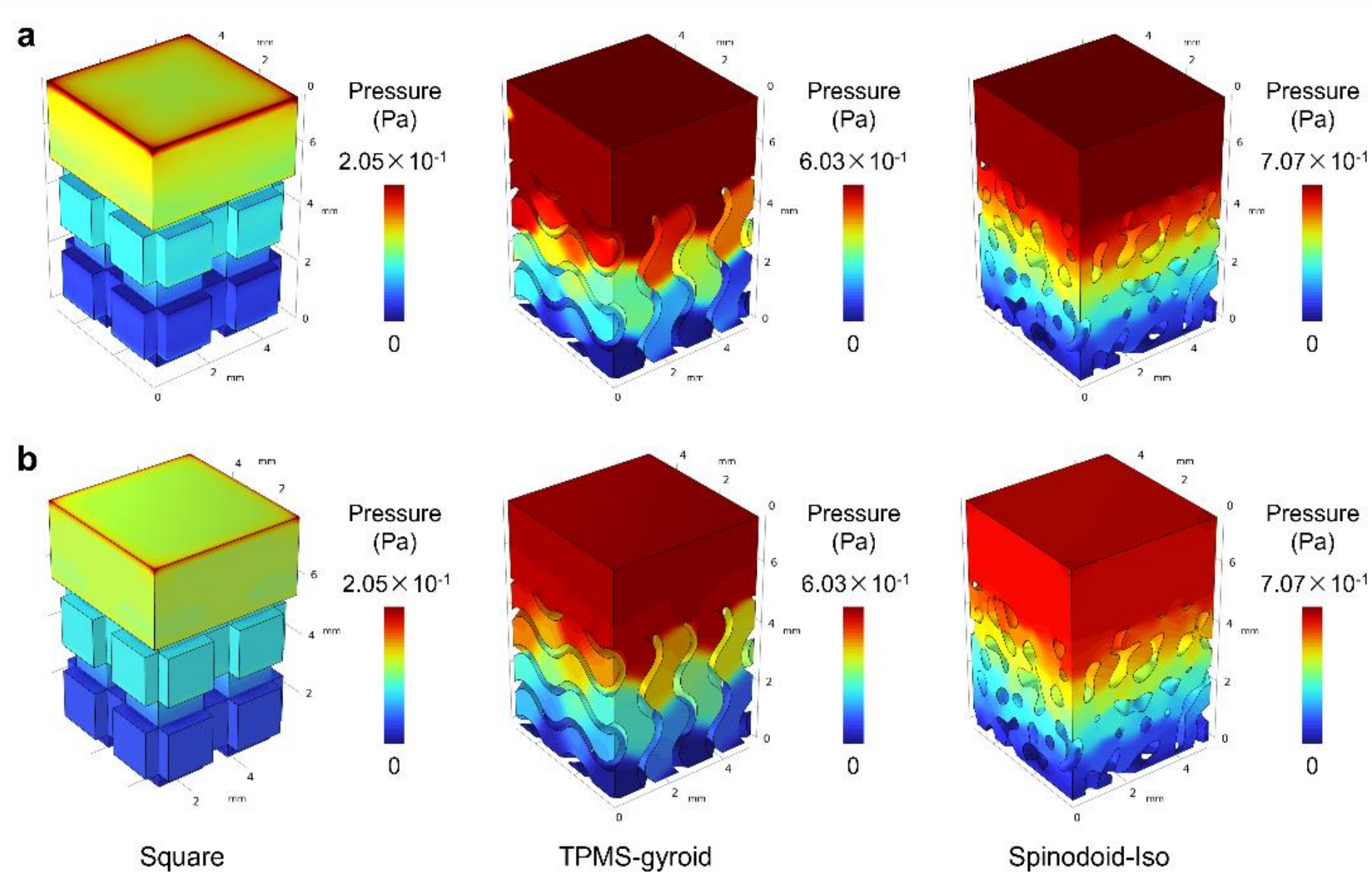

Fig. S52. Simulated pressure distributions within the Square, TPMS-gyroid, and Spinodoid-iso catalysts. a, Bubble flow (laminar), with the hydrostatic contribution removed. **b,** Laminar flow only.

Table S1. Geometry and approximate level set equation of typical TPMS-based lattices.

TPMS	Geometry	Level-set approximation equation
Gyroid	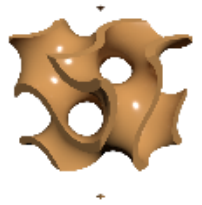	$\sin\left(\frac{\pi x}{a}\right)\cos\left(\frac{\pi y}{a}\right)+\sin\left(\frac{\pi y}{a}\right)\cos\left(\frac{\pi z}{a}\right)+\sin\left(\frac{\pi z}{a}\right)\cos\left(\frac{\pi x}{a}\right)$
I-WP	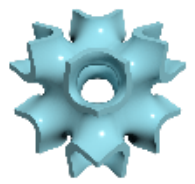	$2\left(\cos\left(\frac{2\pi x}{a}\right)\cos\left(\frac{2\pi y}{a}\right)+\cos\left(\frac{2\pi y}{a}\right)\cos\left(\frac{2\pi z}{a}\right)+\cos\left(\frac{2\pi z}{a}\right)\cos\left(\frac{2\pi x}{a}\right)\right)-\cos\left(\frac{4\pi x}{a}\right)-\cos\left(\frac{4\pi y}{a}\right)-\cos\left(\frac{4\pi z}{a}\right)$
Neovius	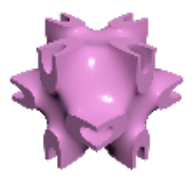	$3\left(\cos\left(\frac{2\pi x}{a}\right)+\cos\left(\frac{2\pi y}{a}\right)+\cos\left(\frac{2\pi z}{a}\right)\right)+4\cos\left(\frac{2\pi x}{a}\right)\cos\left(\frac{2\pi y}{a}\right)\cos\left(\frac{2\pi z}{a}\right)$
Schwarz P	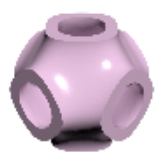	$\cos\left(\frac{2\pi x}{a}\right)+\cos\left(\frac{2\pi y}{a}\right)+\cos\left(\frac{2\pi z}{a}\right)$
Schwarz D	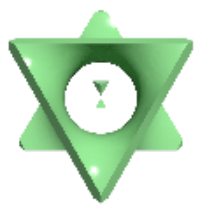	$\sin\left(\frac{\pi x}{a}\right)\sin\left(\frac{\pi y}{a}\right)\sin\left(\frac{\pi z}{a}\right)+\sin\left(\frac{\pi x}{a}\right)\cos\left(\frac{\pi y}{a}\right)\cos\left(\frac{\pi z}{a}\right)+\cos\left(\frac{\pi x}{a}\right)\sin\left(\frac{\pi y}{a}\right)\cos\left(\frac{\pi z}{a}\right)+\cos\left(\frac{\pi x}{a}\right)\cos\left(\frac{\pi y}{a}\right)\sin\left(\frac{\pi z}{a}\right)$

Table S2. Comparison of TPMS and spinodoid structures with other common architectures across key design parameters.

	Honeycomb	Mesh	Foam	Pellet	Square	TPMS	Spinodoid
Connectivity	Connected	Connected	High pore connectivity but uncontrollable distribution	Internally disconnected	Connected	Bicontinuous, periodic, and fully interconnected networks provide unobstructed pass-through channels and minimize dead-end regions	Nonperiodic bicontinuous morphology yields highly diffused flow pathways and suppresses channeling; enhances flow uniformity across the entire volume
Curvature	Zero curvature (flat walls): low interfacial shear and dead zones	High local curvature at intersections causes stress concentration	Continuous curvature but irregular and nonparametric	Spherical external surfaces but severe internal dead zones	Sharp corners generate strong local velocity gradients and stress concentration	Smooth, continuous curvature enables uniform flow and high interfacial shear; zero mean curvature at each point	Smooth, continuous, curvature induces diverse local shear regions; zero mean curvature at each point
Mechanical properties	Strong stress concentration	Mechanically weak and	Low strength and poor reproducibility	Low strength	Strong stress concentration at cell corners	High strength and low stress	Uniform stress distribution and high

	at cell corners	easily deformed				concentration support reusability	energy absorption support reusability
Specific surface area	Limited internal surface area	Moderate	High	Limited internal surface area	Moderate	High and smoothly distributed surface area exposes abundant catalytic sites with uniform accessibility; improves single-atom utilization efficiency	Large, highly tortuous surface area maximizes pollutant–catalyst interaction probability; improves residence time and single-atom utilization efficiency

Table S3. Peak positions and areas of different peaks in high resolution N 1s XPS spectra of CN, Mn-ISAs and Mn-NPs catalysts.

Catalyst	Component	Position (eV)	Area (%)
CN	Pyridinic N	398.5	29.7
	Pyrrolic N	400.7	49.2
	Graphitic N	401.6	21.1
Mn-ISAs	Pyridinic N	398.6	29.8
	Mn-N	399.1	27.2
	Pyrrolic N	400.8	18.9
	Graphitic N	401.8	24.1
Mn-NPs	Pyridinic N	398.5	26.7
	Mn-N	399.2	10.7
	Pyrrolic N	400.6	34.2
	Graphitic N	401.9	28.4

Table S4. EXAFS fitting parameters at the Mn K edge (S_0^2=0.86).

Sample	Path	C.N.	R (Å)	σ^2 (Å^2)	ΔE (eV)	R factor
MnO	Mn-O	6*	2.20±0.01	0.0109	0.7	0.0056
	Mn-Mn	12*	3.14±0.01	0.0099		
Mn-ISAs	Mn-N	4.1±0.2	1.96±0.01	0.0084	-3.8	0.0078

C.N.: coordination numbers; *R*: bond distance; σ^2: Debye-Waller factors; Δ*E*: the inner potential correction. *R* factor: goodness of fit. *: fitting with fixed parameter.

Table S5. Changes in typical components of coal-chemical secondary effluent before and after catalytic ozonation.

Components	Influent	Effluent
Suspended solids	172 mg/L	138 mg/L
Salinity	379 mg/L (as Cl-)	374 mg/L (as Cl-)
Fulvic acid-like humic substances	12.6 %	0.2 %
Humic acid-like humic substances	70.1 %	1.5 %
COD	~110 mg/L	~45 mg/L

Table S6. The catalytic performance comparison of recently reported heterogeneous catalytic ozonation catalysts for contaminants degradation. K value represents the OA degradation rate per unit of active metal component with per unit of reaction time and ozone concentration:

$$\text{K value} = \frac{C_{pollutant} * V_{pollutant} * \eta}{C_{O_3} * Q_{O_3} * t * m_{catalyst} * wt_{metal}}$$

In the formula, $C_{pollutant}$ represents the pollutant concentration, $V_{pollutant}$ represents the wastewater volume, η represents removal efficiency, C_{O_3} represents the ozone concentration, Q_{O_3} represents the ozone flow rate, t represents the reaction time, $m_{catalyst}$ represents catalyst dosage, wt_{metal} represents metal loading.

As summarized from the literature, powder catalysts typically have metal loadings of approximately 1-10 wt% (5-10 wt% for nanoparticle-based and 1-4 wt% for single atom-based), while supported catalysts generally fall within 0.2-2 wt% (1-2 wt% for pellet catalysts and 0.2-1 wt% for fiber-supported catalysts). In comparison, our bioinspired architected catalyst exhibits an ultralow loading of 0.08 wt%, which is significantly below the ranges mentioned above, indicating a remarkably high utilization efficiency of the active metal. Moreover, the bioinspired architected catalyst achieves a much higher catalytic efficiency (K-value = 174.8) than conventional supported catalysts (typically ~ 20), thereby simultaneously realizing high degradation performance and low metal consumption.

Catalyst	Pollutant	Reaction conditions	Reaction Time (min)	Removal efficiency (%)	Metal loading (wt%)	K-value	Ref
Mn-ISAs@Spinodoid	OA	Ozone: 5 mg/L, 0.1 L/min<br>Catalyst: 13 g/L<br>Pollutant: 200 mg/L, 300 mL	60	93	0.08	174.8	This work
Mn-ISAs@Gyroid	OA	Ozone: 5 mg/L, 0.1 L/min<br>Catalyst: 13 g/L<br>Pollutant: 200 mg/L, 300 mL	60	89	0.09	148.7	This work

Ni-C_3N_4<br>Mn-C_3N_4<br>Co-C_3N_4	OA	Ozone: 30 mg/L, 0.1 L/min<br>Catalyst: 0.1 g/L<br>Pollutant: 600 mg/L, 300 mL	60<br>60<br>60	15<br>60<br>95	4.2<br>4<br>3.8	35.7<br>150<br>250	(*68*)
Cu/Co-NiCAF	OA	Ozone: 5 mg/L, 0.5 L/min<br>Catalyst: 25 g/L<br>Pollutant: 100 mg/L, 200 mL	10	85	0.49	5.6	(*69*)
Cu-g-C_3N_4	OA	Ozone: 20 mg/L, 0.1 L/min<br>Catalyst: 0.1 g/L<br>Pollutant: 100 mg/L, 250 mL	60	95	5.2	38.1	(*70*)
Fe-N_4	OA	Ozone: 10 mg/L, 0.2 L/min<br>Catalyst: 0.05 g/L<br>Pollutant: 100 mg/L, 300 mL	60	90	0.65	230.8	(*27*)
Mn-Al_2SiO_5	OA	Ozone: 15 mg/L, 0.4 L/min<br>Catalyst: 10 g/L<br>Pollutant: 100 mg/L, 250 mL	60	95	0.85	0.78	(*14*)
Fe-N-CAF-h	OA	Ozone: 10 mg/L, 1 L/min<br>Catalyst: 4 g/L<br>Pollutant: 100 mg/L, 250 mL	60	75	1.51	0.52	(*71*)
Fe-g-C_3N_4	OA	Ozone: 30 mg/L, 0.1 L/min<br>Catalyst: 0.1 g/L<br>Pollutant: 180 mg/L, 300 mL	45	95	9.1	41.8	(*72*)
Co-C_3N_4	OA	Ozone: 30 mg/L, 0.1 L/min<br>Catalyst: 0.1 g/L	60	95	8.5	33.5	(*73*)

		Pollutant: 180 mg/L, 300 mL					
Mn-N_4	OA	Ozone: 30 mg/L, 0.1 L/min<br>Catalyst: 0.25 g/L<br>Pollutant: 180 mg/L, 250 mL	60	95	5.2	18.3	(74)
Fe-SBA-15	OA	Ozone: 30 mg/L, 0.1 L/min<br>Catalyst: 0.25 g/L<br>Pollutant: 180 mg/L, 250 mL	60	65	1.5	43.6	(43)
Mn-CSM	OA	Ozone: 10 mg/L, 1 L/min<br>Catalyst: 2 g/L<br>Pollutant: 100 mg/L, 200 mL	60	90	0.3	5	(75)
Mn-CSF	OA	Ozone: 10 mg/L, 1 L/min<br>Catalyst: 0.2 g/L<br>Pollutant: 100 mg/L, 250 mL	60	90	2.1	8.9	(76)
NiOOH	OA	Ozone: 12.2 mg/L, 0.5 L/min<br>Catalyst: 0.1 g/L<br>Pollutant: 80 mg/L, 1000 mL	60	92	40	5.1	(77)
MnO_x/g-C_3N_4	OA	Ozone: 8.9 mg/L, 0.5 L/min<br>Catalyst: 0.1 g/L<br>Pollutant: 80 mg/L, 500 mL	60	88	5.8	22.7	(78)
MnO_2	OA	Ozone: 10 mg/L, 0.5 L/min<br>Catalyst: 0.5 g/L<br>Pollutant: 500 mg/L, 500 mL	60	85	30	4.7	(79)
Fe_2O_3/Al_2O_3	OA	Ozone: 30 mg/L, 1 L/min	120	30	1.49	1.1	(80)

		Catalyst: 3.75 g/L<br>Pollutant: 720 mg/L, 1000 mL					
Co/Al_2O_3	OA	Ozone: 30 mg/L, 1 L/min<br>Catalyst: 1.25 g/L<br>Pollutant: 720 mg/L, 800 mL	90	30	1.8	2.84	(*81*)
Co/Fe_3O_4	OA	Ozone: 30 mg/L, 0.2 L/min<br>Catalyst: 1 g/L<br>Pollutant: 4.5 mg/L, 800 mL	20	60	1.3	1.38	(*81*)
Mn-CoOOH-ACF	CA	Ozone: 200 mg/L, 0.1 L/min<br>Catalyst: 0.5 g/L<br>Pollutant: 100 mg/L, 250 mL	30	78	0.39	16.7	(*82*)
MnO_2/SiO_2	Phenol	Ozone: 6 mg/L, 0.8 L/min<br>Catalyst: 4 g/L<br>Pollutant: 90 mg/L, 100 mL	90	86	0.8	0.5	(*83*)
Fe/SiO_2	OA	Ozone: 5 mg/L, 0.8 L/min<br>Catalyst: 1 g/L<br>Pollutant: 10 mg/L, 500 mL	60	73	0.8	1.9	(*84*)
Mn/CF	OA	Ozone: 5 mg/L, 0.8 L/min<br>Catalyst: 1 g/L<br>Pollutant: 20 mg/L, 500 mL	60	93	0.7	5.5	(*85*)

Table S7. DLP printing parameters of Al_2O_3 resin.

Image Number	Exposure time (s)	Intensity (mW/cm^2)	Platform descent distance (mm)	Platform ascent distance (mm)	Operating cycles per layer of roller
1	5.0	15	0.00	0.00	2
2-15	3.0	10	7.00	6.98	2
16-20	8.0	15	7.00	6.98	2
21-50	4.5	12	7.00	6.98	2
51-270	4.0	10	7.00	6.98	1

Table S8. Quantitative comparison of Mn loading, BET surface area, pore diameter, and pore volume for Mn-ISAs and Mn-NPs catalysts.

Sample	Mn (wt%)	BET Surface area ($m^2\ g^{-1}$)	Average Pore Diameter (nm)	Total Pore Volume ($cm^3\ g^{-1}$)
Mn-ISAs	0.08	72.3	3.76	0.068
Mn-NPs	0.5	56.1	6.35	0.089

Movie S1.

The formation of the MnN_4-OH intermediate in the first step

Movie S2.

The formation of the •OH in the second step

REFERENCES

1. R. P. Schwarzenbach, B. I. Escher, K. Fenner, T. B. Hofstetter, C. A. Johnson, U. von Gunten, B. Wehrli, The challenge of micropollutants in aquatic systems. *Science* **313**, 1072–1077 (2006).

2. D. Griggs, M. Stafford-Smith, O. Gaffney, J. Rockström, M. C. Öhman, P. Shyamsundar, W. Steffen, G. Glaser, N. Kanie, I. Noble, Sustainable development goals for people and planet. *Nature* **495**, 305–307 (2013).

3. B. C. Hodges, E. L. Cates, J. H. Kim, Challenges and prospects of advanced oxidation water treatment processes using catalytic nanomaterials. *Nat. Nanotechnol.* **13**, 642–650 (2018).

4. D. B. Miklos, C. Remy, M. Jekel, K. G. Linden, J. E. Drewes, U. Hübner, Evaluation of advanced oxidation processes for water and wastewater treatment – A critical review. *Water Res.* **139**, 118–131 (2018).

5. J. Deng, H. B. Li, S. H. Wang, D. Ding, M. S. Chen, C. Liu, Z. Q. Tian, K. S. Novoselov, C. Ma, D. H. Deng, X. H. Bao, Multiscale structural and electronic control of molybdenum disulfide foam for highly efficient hydrogen production. *Nat. Commun.* **8**, 14430 (2017).

6. K. J. Wei, L. Wang, L. K. Gu, Q. Q. Liu, W. Li, Z. Y. Zhou, W. Q. Han, C. P. Ouyang, R. F. Zhang, X. Huang, X. Y. Zhang, 2D-Like catalyst with a micro-nanolinked functional surface for water purification. *Environ. Sci. Technol.* **58**, 3007–3018 (2024).

7. Z. S. Zhu, S. Zhong, C. Cheng, H. Y. Zhou, H. Q. Sun, X. G. Duan, S. B. Wang, Microenvironment engineering of heterogeneous catalysts for liquid-phase environmental catalysis. *Chem. Rev.* **124**, 11348–11434 (2024).

8. W. Yao, J. M. Chen, Y. J. Wang, R. Q. Fang, Z. Qin, X. F. Yang, L. Y. Chen, Y. W. Li, Nitrogen-doped carbon composites with ordered macropores and hollow walls. *Angew. Chem. Int. Ed.* **60**, 23729–23734 (2021).

9. Y. Meng, Y. Q. Liu, C. Wang, Y. Si, Y. J. Wang, W. Q. Xia, T. Liu, X. Cao, Z. Y. Guo, J. J. Chen, W. W. Li, Nanoconfinement steers nonradical pathway transition in single atom fenton-like catalysis for improving oxidant utilization. *Nat. Commun.* **15**, 5314 (2024).

10. C. H. Gu, S. Wang, A. Y. Zhang, C. Liu, J. Jiang, H. Q. Yu, Tuning electronic structure of metal-free dual-site catalyst enables exclusive singlet oxygen production and in-situ utilization. *Nat. Commun.* **15**, 5771 (2024).

11. Z. Y. Wu, P. Zhu, D. A. Cullen, Y. F. Hu, Q. Q. Yan, S. C. Shen, F. Y. Chen, H. R. Yu, M. Shakouri, J. D. Arregui-Mena, A. Ziabari, A. R. Paterson, H. W. Liang, H. T. Wang, A general synthesis of single atom catalysts with controllable atomic and mesoporous structures. *Nat. Synth.* **1**, 658–667 (2022).

12. Y. Z. Cheng, Z. L. Chen, S. B. Wang, X. G. Duan, Single atom catalysts for heterogeneous catalytic ozonation. *Curr. Opin. Chem. Eng.* **41**, 100945 (2023).

13. Y. Yu, S. Z. Pei, J. N. Zhang, N. Q. Ren, S. J. You, Bio-inspired porous composite electrode for enhanced mass transfer and electrochemical water purification by modifying local flow pattern. *Adv. Funct. Mater.* **33**, 2214725 (2023).

14. T. F. Ren, C. P. Ouyang, Z. Y. Zhou, S. N. Chen, M. X. Yin, X. Huang, X. Y. Zhang, Mn-doped carbon-Al_2SiO_5 fibers enable catalytic ozonation for wastewater treatment: Interface modulation and mass transfer enhancement. *J. Hazard. Mater.* **460**, 132307 (2023).

15. Z. S. Zhu, Y. T. Wang, X. G. Duan, P. T. Wang, S. Zhong, S. Y. Ren, X. Xu, B. Y. Gao, J. Vongsvivut, S. B. Wang, Atomic-level engineered cobalt catalysts for fenton-Like reactions: Synergy of single atom metal sites and nonmetal-bonded functionalities. *Adv. Mater.* **36**, e2401454 (2024).

16. J. Zhu, P. W. Wu, Y. H. Chao, J. T. Yu, W. S. Zhu, Z. C. Liu, C. M. Xu, Recent advances in 3D printing for catalytic applications. *Chem. Eng. J.* **433**, 134341 (2022).

17. J. U. Surjadi, C. M. Portela, Enabling three-dimensional architected materials across length scales and timescales. *Nat. Mater.* **24**, 493–505 (2025).

18. J. Bauer, L. R. Meza, T. A. Schaedler, R. Schwaiger, X. Y. Zheng, L. Valdevit, Nanolattices: An emerging class of mechanical metamaterials. *Adv. Mater.* **29**, 1701850 (2017).

19. J. H. Shin, J. H. Heo, S. Jeon, J. H. Park, S. Kim, H. W. Kang, Bio-inspired hollow PDMS sponge for enhanced oil-water separation. *J. Hazard. Mater.* **365**, 494–501 (2019).

20. F. X. Xie, X. L. Cui, X. Zhi, D. Z. Yao, B. Johannessen, T. Lin, J. N. Tang, T. B. F. Woodfield, L. Gu, S. Z. Qiao, A general approach to 3D-printed single-atom catalysts. *Nat. Synth.* **2**, 129–139 (2023).

21. C. Feng, R. An, H. Xiao, T. Ji, Y. Hu, K. Yan, K. Liao, H. Gao, Z. Xu, J. Tian, Greening fine chemical production via continuous flow technology: An LCA-TEA-SA integrated assessment of *p*-nitrobenzoic acid. *ACS Sustain. Chem. Eng.* **13**, 21927–21937 (2025).

22. R. Xing, R. Huang, R. Su, J. Kong, M. D. Dickey, W. Qi, 3D-printing of hierarchical porous copper-based metal-organic-framework structures for efficient fixed-bed catalysts. *Chem. Bio. Eng.* **1**, 264–273 (2024).

23. W. Yang, G. Y. Dang, M. Fujii, C. Cai, Y. Wei, Y. R. Zhang, Heterogeneous activation of sulfite by a three-dimensional printed hierarchically porous copper catalyst for the degradation of tetracycline hydrochloride in water. *ACS EST Water* **4**, 1730–1740 (2024).

24. Y. N. Shang, X. Xu, B. Y. Gao, S. B. Wang, X. G. Duan, Single-atom catalysis in advanced oxidation processes for environmental remediation. *Chem. Soc. Rev.* **50**, 5281–5322 (2021).

25. Z. L. Weng, Y. F. Lin, S. Y. Guo, X. F. Zhang, Q. Guo, Y. Luo, X. W. Ou, J. X. Ma, Y. Zhou, J. Jiang, B. Han, Site engineering of covalent organic frameworks for regulating peroxymonosulfate activation to generate singlet oxygen with 100% selectivity. *Angew. Chem. Int. Ed.* **62**, e202310934 (2023).

26. Z. W. Yao, Y. D. Chen, X. D. Wang, K. S. Hu, S. Y. Ren, J. Q. Zhang, Z. Song, N. Q. Ren, X. G. Duan, High-entropy alloys catalyzing polymeric transformation of water pollutants with remarkably improved electron utilization efficiency. *Nat. Commun.* **16**, 148 (2025).

27. T. F. Ren, M. X. Yin, S. N. Chen, C. P. Ouyang, X. Huang, X. Y. Zhang, Single-atom Fe-N_4 sites for catalytic ozonation to selectively induce a nonradical pathway toward wastewater purification. *Environ. Sci. Technol.* **57**, 3623–3633 (2023).

28. X. Hai, Y. Zheng, Q. Yu, N. Guo, S. B. Xi, X. X. Zhao, S. Mitchell, X. H. Luo, V. Tulus, M. Wang, X. Y. Sheng, L. B. Ren, X. D. Long, J. Li, P. He, H. H. Lin, Y. G. Cui, X. N. Peng, J. W. Shi, J. Wu, C. Zhang, R. Q. Zou, G. Guillén-Gosálbez, J. Pérez-Ramírez, M. J. Koh, Y. Zhu, J. Li, J. Lu, Geminal-atom catalysis for cross-coupling. *Nature* **622**, 754–760 (2023).

29. T. Yang, H. Chen, Z. Jia, Z. Deng, L. Chen, E. M. Peterman, J. C. Weaver, L. Li, A damage-tolerant, dual-scale, single-crystalline microlattice in the knobby starfish, *Protoreaster nodosus*. *Science* **375**, 647–652 (2022).

30. R. Oftadeh, M. Perez-Viloria, J. C. Villa-Camacho, A. Vaziri, A. Nazarian, Biomechanics and mechanobiology of trabecular bone: A review. *J. Biomech. Eng.* **137**, 010802 (2015).

31. Y. J. Wang, X. Zhang, Z. H. Li, H. J. Gao, X. Y. Li, Achieving the theoretical limit of strength in shell-based carbon nanolattices. *Proc. Natl. Acad. Sci. U.S.A.* **119**, e2119536119 (2022).

32. Z. H. Fan, R. J. Gao, S. T. Liu, A novel battery thermal management system based on P type triply periodic minimal surface. *Int. J. Heat Mass Transf.* **194**, 123090 (2022).

33. H. Montazerian, M. G. A. Mohamed, M. M. Montazeri, S. Kheiri, A. S. Milani, K. Kim, M. Hoorfar, Permeability and mechanical properties of gradient porous PDMS scaffolds fabricated by 3D-printed sacrificial templates designed with minimal surfaces. *Acta Biomater.* **96**, 149–160 (2019).

34. A. G. Izard, J. Bauer, C. Crook, V. Turlo, L. Valdevit, Ultrahigh energy absorption multifunctional spinodal nanoarchitectures. *Small* **15**, 1903834 (2019).

35. Y. F. Zhang, M. T. Hsieh, L. Valdevit, Mechanical performance of 3D printed interpenetrating phase composites with spinodal topologies. *Compos. Struct.* **263**, 113693 (2021).

36. J. Bauer, M. Sala-Casanovas, M. Amiri, L. Valdevit, Nanoarchitected metal/ceramic interpenetrating phase composites. *Sci. Adv.* **8**, eabo3080 (2022).

37. F. V. Senhora, E. D. Sanders, G. H. Paulino, Optimally-tailored spinodal architected materials for multiscale design and manufacturing. *Adv. Mater.* **34**, 2109304 (2022).

38. J. R. Yang, D. Q. Zeng, Q. G. Zhang, R. F. Cui, M. Hassan, L. Q. Dong, J. Li, Y. L. He, Single Mn atom anchored on N-doped porous carbon as highly efficient fenton-like catalyst for the degradation of organic contaminants. *Appl. Catal. Environ.* **279**, 119363 (2020).

39. G. Wang, C. T. He, R. Huang, J. J. Mao, D. S. Wang, Y. D. Li, Photoinduction of Cu single atoms decorated on UiO-66-NH_2 for enhanced photocatalytic reduction of CO_2 to liquid fuels. *J. Am. Chem. Soc.* **142**, 19339–19345 (2020).

40. J. Z. Li, M. J. Chen, D. A. Cullen, S. Hwang, M. Y. Wang, B. Y. Li, K. X. Liu, S. Karakalos, M. Lucero, H. G. Zhang, C. Lei, H. Xu, G. E. Sterbinsky, Z. X. Feng, D. Su, K. L. More, G. F. Wang, Z. B. Wang, G. Wu, Atomically dispersed manganese catalysts for oxygen reduction in proton-exchange membrane fuel cells. *Nat. Catal.* **1**, 935–945 (2018).

41. S. Ma, Q. Tang, Q. X. Feng, J. Song, X. X. Han, F. Y. Guo, Mechanical behaviours and mass transport properties of bone-mimicking scaffolds consisted of gyroid structures manufactured using selective laser melting. *J. Mech. Behav. Biomed. Mater.* **93**, 158–169 (2019).

42. A. J. Beaudoin, W. M. Mihalko, W. R. Krause, Finite-element modeling of polymethylmethacrylate flow through cancellous bone *J. Biomech.* **24**, 127–136 (1991).

43. Z. Jeirani, J. Soltan, Improved formulation of Fe-MCM-41 for catalytic ozonation of aqueous oxalic acid. *Chem. Eng. J.* **307**, 756–765 (2017).

44. Y. Cheng, J. Zhou, Z. Chen, J. Bi, S. Ren, S. Zhong, P. Yan, Y. Li, S. Wang, X. Duan, Single-atom copper@carbon nanospheres for catalytic ozonation: Parallel dual surface oxidation pathways for broad-spectrum water pollutant removal. *Environ. Sci. Technol.* **59**, 21738–21748 (2025).

45. Y. X. Wang, X. G. Duan, Y. B. Xie, H. Q. Sun, S. B. Wang, Nanocarbon-based catalytic ozonation for aqueous oxidation: Engineering defects for active sites and tunable reaction pathways. *ACS Catal.* **10**, 13383–13414 (2020).

46. L. Yuan, J. M. Shen, Z. L. Chen, X. H. Guan, Role of Fe/pumice composition and structure in promoting ozonation reactions. *Appl. Catal. Environ.* **180**, 707–714 (2016).

47. J. H. Cui, Z. Q. Hao, Y. Q. Wang, L. X. Xue, H. Xue, L. H. Tu, L. J. Hao, M. Z. Tian, J. Z. Guo, D. Zhao, G. K. Li, H. Ding, Mechanism of ozone-assisted catalytic oxidation of isopropanol over single-atom platinum catalysts at ambient temperature. *Chem. Eng. J.* **446**, 136989 (2022).

48. T. Lu, A comprehensive electron wavefunction analysis toolbox for chemists, Multiwfn. *J. Chem. Phys.* **161**, 082503 (2024).

49. L. Zhao, Z. Z. Sun, J. Ma, Novel relationship between hydroxyl radical initiation and surface group of ceramic honeycomb supported metals for the catalytic ozonation of nitrobenzene in aqueous solution. *Environ. Sci. Technol.* **43**, 4157–4163 (2009).

50. G. F. Yu, Y. X. Wang, H. B. Cao, H. Zhao, Y. B. Xie, Reactive oxygen species and catalytic active sites in heterogeneous catalytic ozonation for water purification. *Environ. Sci. Technol.* **54**, 5931–5946 (2020).

51. J. S. Bing, C. Hu, Y. L. Nie, M. Yang, J. H. Qu, Mechanism of catalytic ozonation in Fe_2O_3/Al_2O_3@SBA-15 aqueous suspension for destruction of ibuprofen. *Environ. Sci. Technol.* **49**, 1690–1697 (2015).

52. G. F. Yu, Y. Q. Wu, H. B. Cao, Q. F. Ge, Q. Dai, S. H. Sun, Y. B. Xie, Insights into the mechanism of ozone activation and singlet oxygen generation on N-doped defective nanocarbons: A DFT and machine learning study. *Environ. Sci. Technol.* **56**, 7853–7863 (2022).

53. C. He, J. Wang, C. Wang, C. Zhang, P. Hou, X. Xu, Catalytic ozonation of bio-treated coking wastewater in continuous pilot-and full-scale system: Efficiency, catalyst deactivation and in-situ regeneration. *Water Res.* **183**, 116090 (2020).

54. X. Kong, S. Garg, G. Chen, T. D. Waite, Investigation of the deactivation and regeneration of an Fe_2O_3/Al_2O_3• SiO_2 catalyst used in catalytic ozonation of coal chemical industry wastewater. *J. Hazard. Mater.* **451**, 131194 (2023).

55. G. Shen, M. Li, Y. Chen, Z. Xu, X. Wang, B. Dai, Effect of pyrolysis conditions on the MOFs-derived zinc-based catalysts in acetylene acetoxylation. *Catalysts* **13**, 532 (2023).

56. H. Yang, S. Bradley, A. Chan, G. Waterhouse, T. Nann, P. Kruger, S. Telfer, Catalytically active bimetallic aanoparticles supported on porous carbon capsules derived from metal-organic framework composites. *J. Am. Chem. Soc.* **138**, 11872–11881 (2016).

57. E08 Committee, "E1820 standard test method for measurement of fracture toughness" (ASTM International, 2017).

58. F. S. L. Bobbert, K. Lietaert, A. A. Eftekhari, B. Pouran, S. M. Ahmadi, H. Weinans, A. A. Zadpoor, Additively manufactured metallic porous biomaterials based on minimal surfaces: A unique combination of topological, mechanical, and mass transport properties. *Acta Biomater.* **53**, 572–584 (2017).

59. O. Al-Ketan, R. K. Abu Al-Rub, Multifunctional mechanical metamaterials based on triply periodic minimal surface lattices. *Adv. Eng. Mater.* **21**, 1900524 (2019).

60. S. Kumar, S. H. Tan, L. Zheng, D. M. Kochmann, Inverse-designed spinodoid metamaterials. *npj Comput. Mater.* **6**, 73 (2020).

61. COMSOL AB, *The CFD Module User's Guide* (COMSOL Multiphysics v. 6.2, COMSOL AB, 2024).

62. H. Hikita, S. Asai, K. Tanigawa, K. Segawa, M. Kitao, Gas hold-up in bubble columns. *Chem. Eng. J.* **20**, 59–67 (1980).

63. S. Kattel, P. Atanassov, B. Kiefer, Catalytic activity of Co-N_x/C electrocatalysts for oxygen reduction reaction: A density functional theory study. *Phys. Chem. Chem. Phys.* **15**, 148–153 (2013).

64. W. Chen, P. Westerhoff, J. A. Leenheer, K. Booksh, Fluorescence excitation–emission matrix regional integration to quantify spectra for dissolved organic matter. *Environ. Sci. Technol.* **37**, 5701–5710 (2003).

65. J. Peuravuori, K. Pihlaja, Molecular size distribution and spectroscopic properties of aquatic humic substances. *Anal. Chim. Acta* **337**, 133–149 (1997).

66. P. E. Kolic, E. D. Roy, J. R. White, R. L. Cook, Spectroscopic measurements of estuarine dissolved organic matter dynamics during a large-scale mississippi river flood diversion. *Sci. Total Environ.* **485**, 518–527 (2014).

67. K. C. Lu, T. F. Ren, N. Yan, X. Huang, X. Y. Zhang, Revisit the role of salinity in heterogeneous catalytic ozonation: The trade-off between reaction inhibition and mass transfer enhancement. *Environ. Sci. Technol.* **57**, 18888–18897 (2023).

68. J. Wang, Y. B. Xie, G. F. Yu, L. C. Yin, J. D. Xiao, Y. X. Wang, W. G. Lv, Z. Sun, J. H. Kim, H. B. Cao, Manipulating selectivity of hydroxyl radical generation by single- atom catalysts in catalytic ozonation: Surface or solution. *Environ. Sci. Technol.* **56**, 17753–17762 (2022).

69. K. J. Wei, X. X. Cao, W. C. Gu, P. Liang, X. Huang, X. Y. Zhang, Ni-induced C-Al_2O_3-framework (Ni_{CAF}) supported core-multishell catalysts for efficient catalytic ozonation: A structure-to-performance study. *Environ. Sci. Technol.* **53**, 6917–6926 (2019).

70. Z. Guo, J. Wei, Z. K. Wu, Y. F. Guo, Y. H. Song, Stabilized N coordinated Cu site in catalytic ozonation: The efficient generation of OH induced by surface hydroxyl groups based on the Lewis acid site. *Sep. Purif. Technol.* **304**, 122215 (2023).

71. C. P. Ouyang, K. J. Wei, X. Huang, M. G. El-Din, X. Y. Zhang, Bifunctional Fe for induced graphitization and catalytic ozonation based on a Fe/N-doped carbon-Al_2O_3 framework: Theoretical calculations guided catalyst design and optimization. *Environ. Sci. Technol.* **55**, 11236–11244 (2021).

72. H. B. Cao, J. Wang, J. H. Kim, Z. Guo, J. D. Xiao, J. Yang, J. J. Chang, Y. C. Shi, Y. B. Xie, Different roles of Fe atoms and nanoparticles on g-C_3N_4 in regulating the reductive activation of ozone under visible light. *Appl. Catal. Environ.* **296**, 120362 (2021).

73. J. Wang, G. F. Yu, Y. C. Wang, S. N. Liu, J. K. Qiu, Y. J. Xu, Z. Wang, Y. X. Wang, Y. B. Xie, H. B. Cao, Surface density of cobalt single atoms manipulating hydroxyl radical generation via dual pathways: Electrons supply and active sites. *Adv. Funct. Mater.* **33**, 2215245 (2023).

74. Z. Guo, Y. B. Xie, J. D. Xiao, Z. J. Zhao, Y. X. Wang, Z. M. Xu, Y. Zhang, L. C. Yin, H. B. Cao, J. L. Gong, Single-atom Mn-N_4 site-catalyzed peroxone reaction for the efficient production of hydroxyl radicals in an acidic solution. *J. Am. Chem. Soc.* **141**, 12005–12010 (2019).

75. S. N. Chen, T. F. Ren, Z. Y. Zhou, K. C. Lu, X. Huang, X. Y. Zhang, Insights into Mn loaded carbon-silica-membrane based catalytic ozonation process for efficient wastewater treatment: Performance and mechanism. *Chem. Eng. J.* **475**, 145874 (2023).

76. S. N. Chen, T. F. Ren, X. Y. Zhang, Z. Y. Zhou, X. Huang, X. Y. Zhang, Efficient catalytic ozonation via Mn-loaded C-SiO_2 framework for advanced wastewater treatment: Reactive oxygen species evolution and catalytic mechanism. *Sci. Total Environ.* **858**, 159447 (2023).

77. M. Y. Luo, Y. Huang, J. W. Jiang, Catalytic ozonation of oxalic acid in water with nickel oxyhydroxide (NiOOH). *Water Purif. Technol.* **37**, 71–76 (2018).

78. Y. F. Yang, Y. X. Huang, J. Yao, Y. Mu, Degradation of oxalic acid in water by catalytic ozonation with manganese nitride carbon catalyst. *J. Water Resour. Water Eng.* **28**, 45–49 (2017).

79. H. M. Zhang, Q. Z. Jia, J. T. Lyu, F. Y. Yan, Q. Wang, Catalytic ozonation of simulated oxalic acid wastewater using MnO_2. *J. Tianjin Univ. Sci. Technol.* **35**, 20–24 (2020).

80. F. J. Beltrán, F. J. Rivas, R. Montero-de-Espinosa, Iron type catalysts for the ozonation of oxalic acid in water. *Water Res.* **39**, 3553–3564 (2005).

81. F. J. Beltrán, F. J. Rivas, R. Montero-De-Espinosa, Ozone-enhanced oxidation of oxalic acid in water with cobalt catalysts. 1. Homogeneous catalytic ozonation. *Ind. Eng. Chem. Res.* **42**, 3210–3217 (2003).

82. J. Y. Ren, Z. Z. Yao, Q. Wei, R. T. Wang, Y. Liu, L. Wang, K. B. Zheng, S. Wang, H. Guo, Z. R. Niu, J. Wang, J. Han, L. Lü, Y. Z. Zhen, J. Li, Degradation of ferulic acid and caffeic acid by dielectric barrier discharge plasma combined with Mn/CoOOH/activated carbon fiber. *Sep. Purif. Technol.* **306**, 122691 (2023).

83. J. Shi, Y. T. Liu, Preparation of flexible ceramic nanofiber composite catalytic material and its application in environmental wastewater. *Proc. SPIE* **12309**, 143–146 (2022).

84. G. Gao, W. Chu, Z. Chen, J. Shen, Catalytic ozonation of diclofenac with iron silicate-loaded pumice in aqueous solution. *Water Sci. Technol. Water Supply* **17**, 1458–1467 (2017).

85. W. Li, “Preparation and properties of nano-catalysts for heterogeneous catalytic ozonation,” thesis, Hebei University of Science and Technology, Shijiazhuang (2017).
